# Pattern of blast injuries. A Systematic Review: Part 1–Improvised, multiple and unspecified explosive devices

**DOI:** 10.1007/s00068-026-03261-x

**Published:** 2026-08-24

**Authors:** Anne Neubert, Max Seidelmann, Catharina Gaeth, Dan Bieler

**Affiliations:** 1 Department of Orthopedics and Traumatology, University Hospital and Medical Faculty, Heinrich-Heine-University Duesseldorf, Moorenstraße 5, 40225 Duesseldorf, Germany; 2 TraumaEvidence@ German Trauma Society, Berlin, Germany; 3 Department of Trauma Surgery and Orthopedics, Hand-, Reconstructive Surgery and Plastic Surgery, German Armed Forces Central Hospital, Medical Campus Koblenz of University Medicine Mainz, Ruebenacher Str. 170, 56072 Koblenz, Germany

**Keywords:** Blast injuries, IED, Military, Multiple explosive devices, War

## Abstract

**Purpose:**

This systematic review aims to comprehensively identify and categorize the specific injury patterns associated with IEDs and other explosive devices across military and civilian populations to improve medical preparedness, resource planning strategies, and treatment approaches.

**Methods:**

A search on PubMed und Scopus was conducted on 26. March 2025 for observational studies reporting blast injuries in civilian and military populations of all ages. Studies were clustered by explosion type – Part 1 IED and multiple/unspecified explosives. A narrative data synthesis was performed. Reporting was assessed with the JBI tool for case series. PROSPERO: CRD42023391736.

**Results:**

33 studies with 4,373 victims (mainly military personnel) from 2002 - 2022 reported injuries caused by IEDs. The average age was 25.1 years. 97.2% were male. Injuries to the upper and lower extremities were predominant in all study populations followed by head injuries among military personnel, and thorax and abdomen injuries among civilians. 37 studies with 24,600 casualties (mean age 25.7, 85.8% males) were included in multiple/unspecified explosives. Extremities were predominant followed by facial injuries among military personnel, and thorax injuries among civilians.

**Conclusion:**

The first part of this systematic review on the pattern of blast injuries shows that injuries to the extremities, including amputations, are the leading injury pattern caused by explosions. The differences in the injured body regions between military personnel and civilians can probably be attributed to the availability or lack of protective equipment.

**Supplementary Information:**

The online version contains supplementary material available at 10.1007/s00068-026-03261-x.

## Introduction

Blunt and penetrating injuries, as well as burns, are among the numerous types of injuries resulting from explosions [1, 2]. In past and ongoing conflicts, such as those in Afghanistan, Iraq, Ukraine, and Israel, explosive weapons—including artillery bombs, rockets, grenades, and improvised explosive devices (IEDs)—continue to be common mechanisms of injury, with blasts being the predominant cause of injuries and fatalities in these conflicts [3, 4]. IEDs, in particular, vary as much as the injuries they cause and play an important role in asymmetric warfare settings.

In recent conflicts in Afghanistan and Iraq, IEDs were the adversary’s preferred weapon, accounting for over half of the combat injuries sustained [1, 5, 6]. For instance, Ursano and colleagues showed that from 2004 to 2009, an average of 1,913 ± 880.29 IED incidents occurred per month, resulting in an average of 36.6 ± 172.49 fatalities or injuries monthly [7]. The objective of this asymmetric warfare by local groups was to inflict casualties among international and local armed forces through the use of IEDs [8].

IEDs are versatile weapons that can be customized in various forms and sizes, making them adaptable to diverse situations. These devices are frequently enhanced with additional materials, such as nails, steel balls, and metal fragments, which result in more extensive damage due to shrapnel effect. Consequently, IEDs can be used against mounted and dismounted soldiers and civilians, for instance, as roadside bombs or vehicle mines. They can be detonated using a timer, pressure, or remotely via a signal, contributing to their unpredictability and significant threat [7].

The medical consequences of injuries caused by IEDs are equally diverse. Generally, blast injuries are classified as primary, secondary, tertiary, quaternary, or quinary based on the effects of the blast wave [9]. Primary blast injuries result from a blast wave or shock front due to a high-pressure wave predominantly affecting air-filled organs, resulting in injuries such as blast lung, bowel contusions, and ruptured tympanic membranes. Secondary blast injuries arise from dynamic pressure, causing injuries from accelerated fragments in the environment. Tertiary blast injuries are caused by blast winds, leading to blunt force trauma or fractures from impact or falls. Individuals in proximity to an explosion may suffer crush injuries or traumatic amputations. Quaternary blast injuries are burns caused by the heat of the explosion, and quinary blast injuries result from contaminants or substances from the bomb itself, including chemical, biological, or radiological substances [2, 10].

The complexity of blast injuries presents major challenges in trauma care and requires diverse yet specific treatment strategies [1]. Many studies have addressed isolated aspects of blast injuries, lacking to outline the holistic and specific injury patterns resulting from various types of explosive devices, especially IEDs.

This systematic review aims to comprehensively identify and categorize the specific injury patterns associated with IEDs and other explosive devices across military and civilian populations to improve medical preparedness, resource planning strategies, and treatment approaches.

## Methods

This systematic review reports according to “Preferred Reporting Items for Systematic reviews and Meta-Analyses” (PRISMA) guidelines (checklist provided in Additional File 1) [11]. The protocol was registered with PROSPERO (registration number: CRD42023391736). Any changes in comparison to the protocol are described in the relevant sections.

### Inclusion criteria

All studies that classified injuries as blast injuries encompassing primary, secondary, tertiary, quaternary and quinary injuries in civilians and military personnel of all age groups were included. Exposure was defined as an explosion. This includes explosions that occurred in relation to combat, suicide bombing, or terror attacks, but also due to specific devices, such as IEDs or landmines. In part 1, studies on IEDs and multiple or unspecified explosions are reported.

The United Nations defines IEDs as “devices placed or fabricated in an improvised manner incorporating destructive, lethal, noxious, pyrotechnic or incendiary chemicals and designed to destroy, incapacitate, harass, or distract. It may incorporate military stores but is normally devised from non-military components” (page 19, IMAS 04.10, Second Edition, 2003). Multiple explosive devices are defined as all explosive ordnances used in conflicts, terror attacks or wars. This may range from rockets to mines, for example. However, the definitions are not static, for example, an IED can “meet the definition of a mine, booby trap, and/or other type of explosive ordnance depending on its construction” (according to page 19, IMAS 04.10, Second Edition, 2003). Unspecified explosives were those explosions not described in detail. These studies referred to the cause of injury as an explosion, blast, or a synonym.

Individuals injured in household explosions or other small explosions such as fireworks, were excluded. In addition, other large unintentional explosions such as the Beirut explosion in 2020 [12], were excluded. Observational studies of retrospective and prospective nature with and without control groups, such as register studies, case series, hospital records, and autopsy reports, were included. Animal, biomechanical and cadaver studies were excluded. Only studies published after 1970 were included.

### Selection

A search of MEDLINE via PubMed and Scopus was performed. The databases were searched from inception to the present. The search terms involved “blast injuries” and “pattern” in combination with synonyms. Where possible, MeSH terms were used. Only publications in English and German were included (Additional File 2 – search strategy). Two authors (AN & MS) independently selected studies based on the predefined inclusion criteria by 1) title/abstract and 2) full text. All disputes were resolved through discussion. The reasons for the exclusion of full texts were noted. The selection was performed using the Covidence Software [13].

### Data extraction

A data extraction sheet was developed and tested for suitability with three studies. Subsequently, the extraction sheet was revised, especially regarding the injury categories [14]. Injuries were recorded according to the AIS regions (see also Table 1 below) [15]. Injury severity was extracted as AIS ≥ 3, AIS 0–2 or as unknown severity if information was missing. Furthermore, we extracted data on population characteristics such as age and sex; details on the explosive device and the explosion itself, if possible (e.g., mounted/dismounted), location, year under investigation, and information on study design and authors details. One author extracted the data from the studies using Microsoft Excel. Another author performed a quality check of all extracted data to identify extraction mistakes, typos, etc. All identified mistakes, issues, and disputes during extraction were discussed within the research team.

**Table 1:** Categorization of injuries

General categories	Included injuries/regions
Head/neck	• Brain and head excluding facial region
Face	• Eyes, ears, facial region (e.g. jaw)
Abdomen	• Excluding spine (if detail is available)
Thorax	• Including bone and internal organ damage • Excluding spine (if detail is available)
Extremities	• Including fractures and amputations • Upper extremities include clavicular and scapular, if reported separately. • Soft tissues injuries excluded, if reported
External incl. burns	• Burns should be extracted separate from other external injuries, if reported
If details are available	
Pelvis	If detailed information is available • Includes all reported fractures
Spine	If detailed information is available • Includes all reported fractures
Urogenital	If detailed information is available • All reported injuries that involve the urogenital tracts

### Critical appraisal of reporting quality

A different risk of bias tool was used than specified in the protocol. Owing to the inclusion of predominantly cross-sectional studies, the MINORS tool by Slim et al. 2003 [16] could not be used as it is most suitable for cohort or case-control studies. We used the JBI tool for case series to assess the reporting quality of studies included, as the heterogeneity of included studies precluded the use of another tool [17]. The tool was used to investigate the reporting quality of the included studies. The assessment was performed independently by two authors (MS & CG). Disputes were resolved through discussion. The categories were judged as reported or not reported. All categories judged as reported were summed. We considered a sum score of less than 5 points as an insufficient reporting quality.

### Synthesis

All included studies were clustered according to one of four types of explosions: improvised explosive device (IED)/multiple or not specified explosive devices/landmines or unexploded ordnance (UXO)/terror attacks and suicide bombers (the last two are comprehensively defined and described in Part 2 [18]). All four categories were analyzed separately. The mechanisms of injury are distinctly different. Hence, separating the categories from each other improves the identification of injury patterns per injury mechanism. If possible, the injuries described in the studies were assigned to one of the body areas, as described in Table 1. Additionally, most studies reported major amputations as proximal to the ankle or wrist, while others reported more distal amputations (e.g., fingers). Due to this heterogeneity, no difference could be observed between minor and major amputations in the current study.

Owing to the high degree of heterogeneity of different circumstances (e.g., civilian versus military casualties, children versus adults), no meta-analyses were conducted. Descriptive statistics were used to calculate patterns for subsets of the data but mostly graphs and tables were used to illustrate the identified patterns of injury.

Owing to the overlap in study populations and missing reporting for many body regions, only studies with large populations and detailed reporting were used for graphs or calculations. Studies from the same register and time frame were disregarded as they investigated the same population or subgroups of the same population. This procedure is reported in the graphs and sections, if used.

## Results

**Fig. 1:**
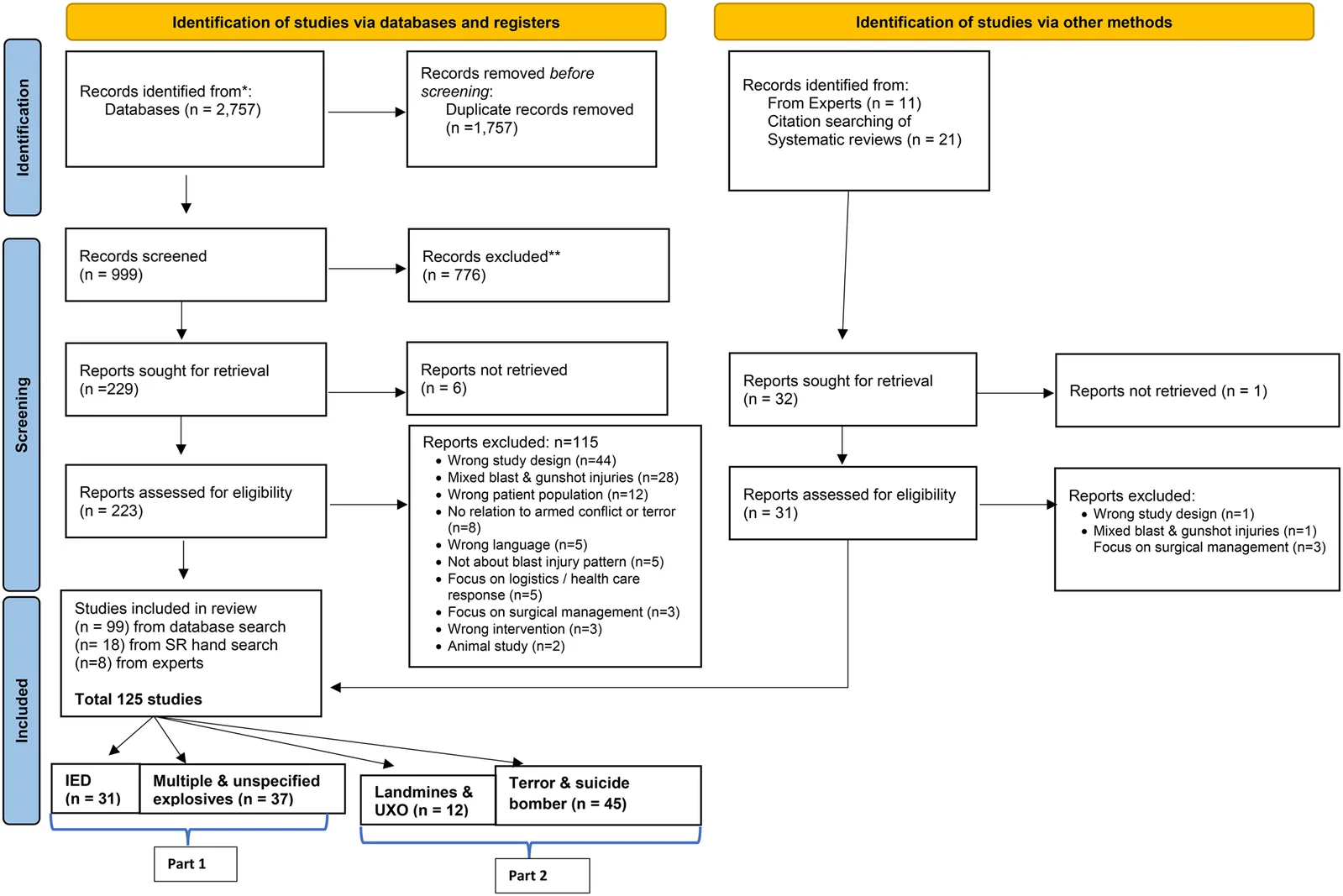
PRISMA flowchart

Two database searches were performed on 6. January 2023, and an update on 26. March 2025 and resulted in 2,756 hits of which 1,757 were identified as duplicates. After title and abstract screening, 223 publications underwent full-text screening, of which 99 fulfilled the inclusion criteria of this systematic review. Manual searches yielded 18 additional studies, and eight more studies were provided by experts. Thus, 125 studies were analyzed in this systematic review. More details are presented in the PRISMA flowchart (Fig. 1). Additional file 3 provides an overview of all excluded full texts including the reasons for exclusion.

Despite some general information on the 125 studies, this part of the systematic review focuses on the 31 studies on IEDs and the 37 studies investigating multiple and unspecified explosive devices. Whereas the remaining 57 studies − 12 studies on landmines and UXO, and 45 studies on terror and suicide bombings - are discussed in “Pattern of Blast injuries - Part 2” (Neubert et al. in prep [18]).

### Characteristics of included studies

All 125 included studies with 126 publications focused on demonstrating the pattern of injuries sustained from explosions. However, the reported explosive devices vary widely from landmines to IEDs, rockets, car bombs, and missiles used in conflict regions or terror attacks around the globe. In each section and in the additional files, extensive information on the populations, explosive devices, and injury patterns is provided.

**Fig. 2:**
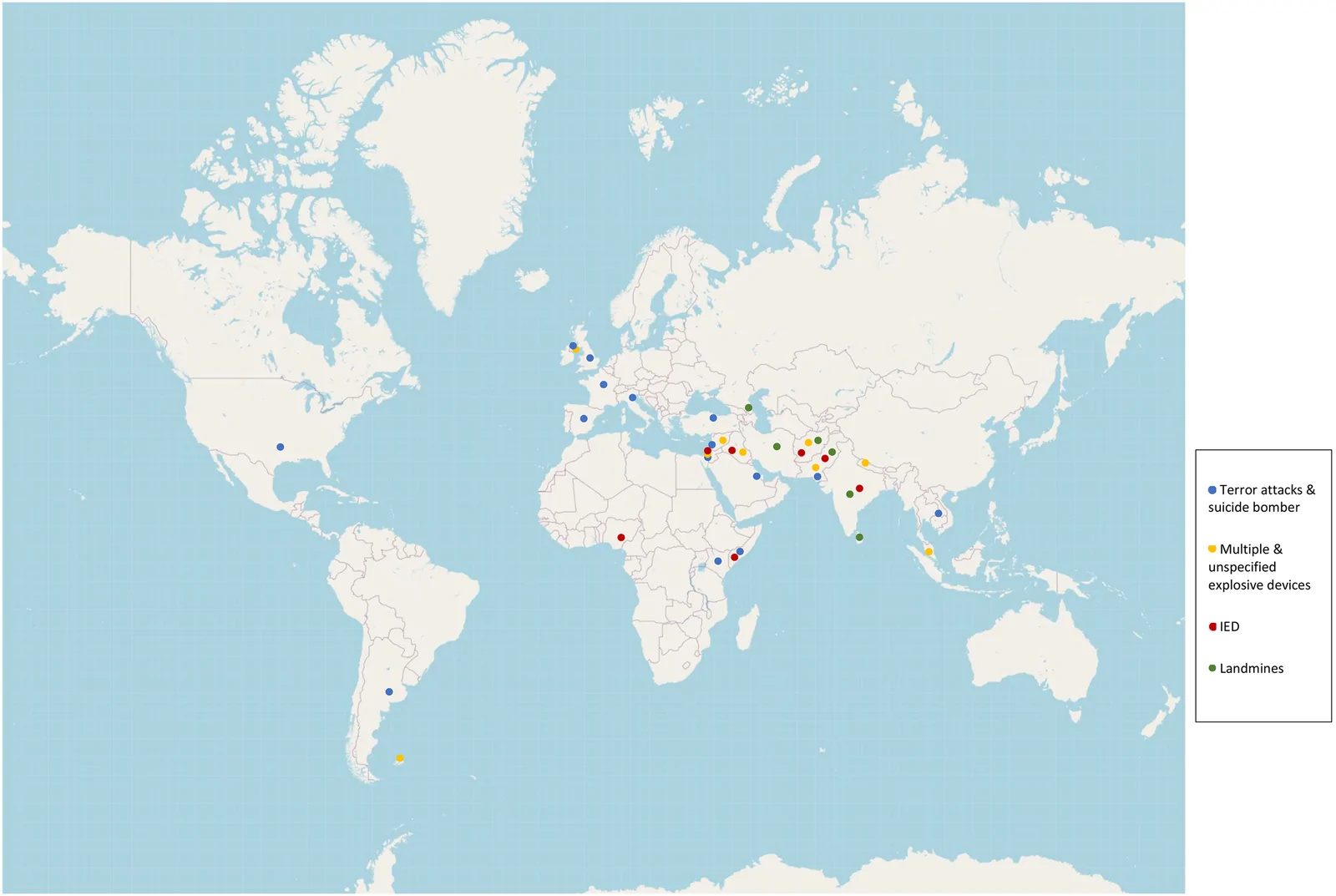
World map by type of explosion

Figure 2 shows a world map depicting the countries reported in the included studies. It demonstrates the distribution across the globe with an accumulation of all categories in the Middle and Far East and an accumulation of studies concerning terror and suicide bombing in Western countries, including amongst others Europe, the USA, and Argentina. The absence of dots in other countries can be due to non-reporting of terror events, the absence of registration organs for landmines, studies in languages other than German or English, and non-inclusion in the search strategy, which focused on blast injuries and not on specific explosive devices. For example, no studies from Cambodia were identified for inclusion, even though this country is highly contaminated with landmines [19]. Further, all identified studies reporting on the conflict in the Ukraine and Gaza did not meet the inclusion criteria.

### Critical appraisal

The median was 9 out of 10 points (range 5–10) indicating good reporting quality (Additional File 4). Only one study was evaluated to have low reporting quality, with 5 out of 10 points. The most frequently missing information was demographic information. In 43 of the 126 included studies, the demographic information of the investigated population was either incomplete or missing (e.g., sex, age, ISS). This was followed by the reporting of the presenting site, such as the exact place of the incident and first clinical admission, which was missing in 13 studies. These studies included multiple patients from different countries without providing information about the location of the injuries. In 14 included studies, the statistical analysis was not complete; for example, the standard deviation of the age or the median was missing. In all studies, injuries as a result of the blast were reported. One issue was the risk of confounding in the included studies. The studies did not report any confounding factors, nor did they plan to investigate confounding factors. Most cross-sectional studies did not include a control group or perform matching. However, some studies included patients with gunshot injuries in the control group. The statistical analyses were mostly descriptive.

## IED

### Study characteristics

31 studies (32 publications) with a total of 4,373 casualties investigated injuries caused by IEDs between 2003 and 2024 (most between 2008 –2010, for two studies, information was missing [20, 21]) [20–50]. 24 studies report incidents from either Afghanistan or Iraq or from Afghanistan and Iraq [21, 22, 25–36, 39–49]. The remaining studies investigated blast events in Somalia, Pakistan, Turkey, and India, as shown in Fig. 2. The mean age across 13 studies, which reported a mean age, is 26.6 years [20, 21, 24, 25, 27, 29, 32, 35, 39, 46, 47, 50]. 21 studies report the sex distribution, with 97.2% of these populations across studies being male (see Table 2). Most studies analyzed registers such as the JTTR [21, 22, 26, 28–30, 32, 34–36, 39, 40, 42, 46–49]. Seven studies used retrospective hospital data [20, 23–25, 31, 38, 45] and three others are case series [33, 41, 44]. The focus is mainly on military personnel, but three studies investigated civilians exclusively [20, 24, 38]. Therefore, most studies included adults, whereas three others included adults and children [27, 37, 50] and one study did not provide any information but likely only included adults [33]. Detailed information is provided in Additional File 4.

**Table 2:** Study characteristics of IED-studies

Study ID	N	Age In years (SD)	Sex (male)	Country	Time frame	Context	Focus of the study	JBI
Abdullahi 2024 [50]	14	Ø 29.5 (±8.8)	78.6%	Nigeria	2016–2022	C&M	Maxillo-facial injuries	10
Ashworth 2023 [48]	46	-	-	Afghanistan & Iraq	2007–2013	M	Fatalities due to head injuries	8
Ashworth 2024 [49]	71	-	-	Afghanistan & Iraq	2007 – 2013	M	Fatalities of dismounted casualties	9
Commandeur 2012 [44]	8	-	100%	Afghanistan	November 2008 – January 2009	M	General Injury pattern	6
Cross 2014 [45]	77	-	100%	Afghanistan	September 2009 – April 2010	C&M	Lower extremity amputations	9
Dupaix 2018 [46]	499	Ø 23.4 (±3.8)	100%	Afghanistan	January 2009 –February 2012	M	Lower extremity amputations	10
Freedman 2014 [47]	65	Ø 29.7 (±8.2)	96.9%	Afghanistan & Iraq	August 2007 –August 2010	M	Thoracolumbar spinal injury	10
Jacobs 2014 [43]	103	-	-	Afghanistan	November 2010 -February 2011	C&M	Lower extremity injuries	9
Mehta 2007 [20]	16	Ø 35.3 (range: 22 - 52)	100%	India	Ø	C	Eye injuries	10
Miller 2022 [37]	74	Mdn 32 (IQR 24, 42)	96%	Israel	January 2006 – July 2020	C	General Injury Pattern	10
Minhas 2014 [38]	70	20–32	100%	Pakistan	February 2014	C	General injury pattern	9
Morrison 2012 [39]	169	Ø 24.0 (± 7)	99.4%	Afghanistan	January 2007 –December 2010	M	Lower limb amputations	10
Mossadegh 2012 [40]	118	Mdn 25 (IQR 21–29)	-	Afghanistan	January 2003 –December 2010	M	Pelvi-perineal trauma	8
Nelson 2007 [41]	18	-	-	Iraq	August 2004 –September 2004	M	General injury pattern	9
Oh 2016 [42]	457	Mdn 23 (IQR 21- 26)	99.8%	Afghanistan	January 2009 –February 2012	M	Extremity amputations	10
Pearce 2017 [35]	426	Ø 24 [22–30]	98.4%	Afghanistan & Iraq	2003 – 2014	M	Torso hemorrhage	9
Penn-Barwell 2014 [36]	43	Mdn 25 (IQR 22–28)	100%	Afghanistan	March 2004 -March 2010	M	Bilateral lower limb amputations	9
Ragel 2009 [31]	19	Mdn 25 (IQR 22–28)	100%	Afghanistan	January 2008 –May 2008	M	Thoraco-lumbar spinal injury	10
Ramasamy 2008 [33]	53	-	-	Iraq	January 2006 –October 2006	C&M	General injury pattern	9
Ramasamy 2012 [32]	89	Ø 25.5 [19–33] (survivors)	100%	Afghanistan & Iraq	August 2008 –August 2010	M	Open pelvic fractures	10
Rankin 2020 [34]	159	Mdn 24 (range 18–48)	100%	Afghanistan & Iraq	2003–2014	C&M	Vascular injuries	9
Salinas 2011 [25]	104	Ø 26.8	-	Iraq	October 2004 –September 2007	M	Facial trauma	9
Singleton 2013 [26]	121	Mdn 25 (IQR 21–29)	99.2%	Afghanistan & Iraq	November 2007- August 2010	M	Fatalities	10
Smith 2017 [27]	100	Ø 25 (range 6–44)	100%	Afghanistan	January 2010 –July 2011	C&M	Lower extremity injuries	10
Spurrier 2015 [21, 51]	78	Ø 26.8 (range 18–55	-	Afghanistan &Iraq	February 2008 –April 2013	M	Spinal fractures	8
Staruch 2017 [28]	380	-	-	Afghanistan	September 2006 – April 2013	M	Hand injuries	9
Stewart 2018 [52]	94	Ø 25	97.9%	Afghanistan & Iraq	2003–2014	M	Head & neck injuries	9
Taddeo 2015 [30]	326	-	-	Afghanistan	March 2008 –May 2011	M	Cervical spine injuries	9
Tahtabasi 2021b [24]	101	Ø 32.2 (±12.7)	75.3%	Somalia	January 2019 –December 2019	C	General injury pattern	10
Ünlü 2015 [23]	133	-	100%	Iraqi border of Turkey	July 2005 – June 2007	C&M	General injury pattern	10
Webster 2018 [22]	342	Mdn 29 (range 18–51)	98.8%	Afghanistan & Iraq	2003–2014	C&M	Pelvic injuries	9

Legend: context: C = civilian, m = military, C&M = mixed population of civilians and military personnel; UK = United Kingdom; US = United States of America; Ø=mean, Mdn = median, IQR = interquartile range; SD = standard deviation

Issues with reporting & risk of bias:

Risk of bias assessment showed a negligible risk of bias, with a median risk of bias score of 9 out of 10 points (Range 6–10, lower risk- higher score).

Amputations were not unequivocally reported in the included studies. It is not always explicit whether the amputation was traumatic or secondary to severe extremity trauma. Furthermore, the number of amputations per person is not reported comprehensively in every study. Some studies reported only the number of persons with amputations but not the total number of amputations per person. It is uncertain whether most studies only report major amputations, hence, excluding finger and toe amputations.

Eighteen studies were from registries such as the JTTR UK or US [21, 22, 26, 28–30, 32, 34–36, 39, 40, 42, 46–49]. These studies mostly investigate overlapping timeframes with military personnel deployed in Iraq and Afghanistan. This hindered a more profound analysis of the injury patterns. Furthermore, many studies only investigated specific body parts or injuries (e.g., head injuries), which hindered a comprehensive picture of the overall pattern of injuries sustained. Information on the circumstances or surrounding factors is often not provided. However, if information regarding mounted and dismounted is not provided, an analysis of the injury pattern is severely hampered.

The sex is reported only rarely especially in military literature, hindering the understanding of some pattern of injuries but also the research of surgical techniques e.g. in relation to high amputation injuries with urogenital involvement and pelvic injuries.

### General injury pattern

**Fig. 3:**
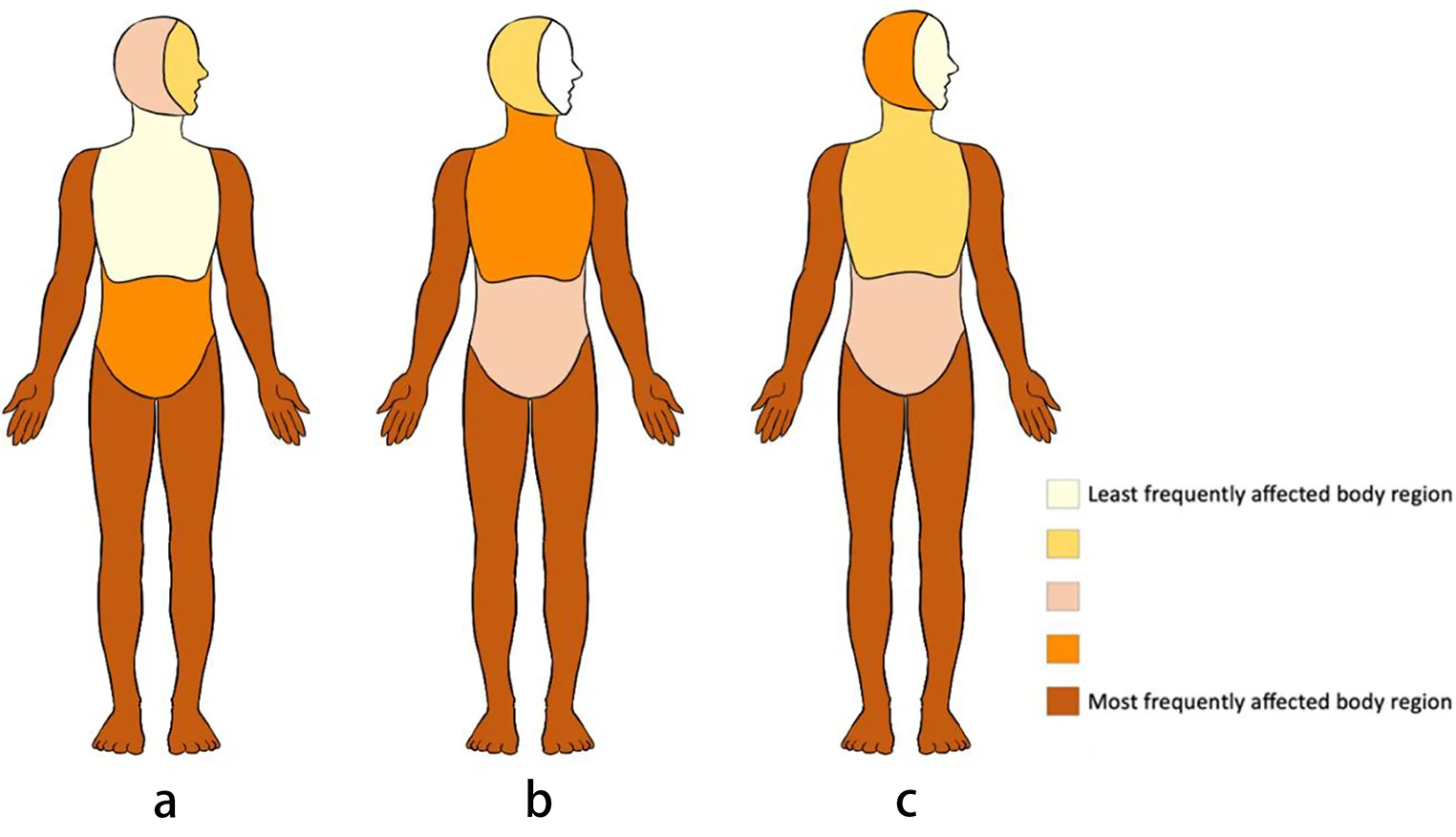
**a**) Civil and military, **b**) Civil population, **c**) Military population

Figure 3 a, b, c displays a ranking of the frequency by which different body regions are affected in the three population groups analyzed – civil, military and a mixed population of civil and military. The figures are based on the study population of 13 studies reporting injuries to at least 4 of 5 AIS body regions (excluding external injuries) with a minimum of 6 patients [22–24, 27, 31, 32, 34, 37, 38, 41–43, 45]. Studies reporting civil populations and studies reporting mixed population of civilians and military personnel show more variability in injury pattern. All populations are suffering predominantly from extremity injuries. Information on external injuries and other details regarding the pattern of injuries in included studies are provided in Additional File 4.

Figure 3 a is based on seven studies reporting on mixed populations [22, 23, 27, 33, 34, 43, 45]. Overall, facial injuries were more frequent in the mixed population than in the separate populations. For example, Ünlü (2015) reported that 17% of people in their cohort had facial injuries. They investigated blast injuries in a Role 2 hospital at the Iraqi border of Turkey [46].

Figure 3 b is based on three studies investigating civilians [24, 37, 38]. In comparison thoracic injuries are more common. Within this cluster, Minhas 2014 reported fewer extremity injuries than other studies. All casualties in this study were injured in a bus. A vehicle packed with explosives detonated next to the bus. This may be due to the mounted injury mechanism, which resulted in fewer extremity injuries [38].

Figure 3c is based on three military studies [31, 41, 42]. Head injuries were more common than those in other population groups. Among the JTTR studies in this cluster, only the study by Oh 2016 was used. This study has the largest population with the broadest pattern of injury reported among the JTTR studies [42].

In comparison, the distribution of injuries shown in the heatmap in Table 3 also demonstrates that extremity injuries are most frequently reported. A high percentage of extremity injuries have been reported in studies on military populations. Lower extremity injuries are more frequent than upper extremity injuries. In addition, amputations appear in several studies at notable rates. Multiple amputations have been reported insufficiently across studies (Additional File 5). Head and thoracic injuries were less consistently reported and varied more widely between studies. Ashworth 2023, Ashworth 2024 and Rankin 2020 show more head injuries (100, 57 and 47% of injured persons, respectively) than other studies [34, 48, 49]. The high number of head injuries in Ashworth 2023 can possibly be explained by the focus on head injuries in underbody blast incidents [48]. Similarly, Ashworth 2024 analyzed head and spinal fractures in dismounted casualties [49]. Studies on military personnel tend to focus more on specific injury patterns (e.g., more consistent extremity injuries), whereas studies on civilians display a higher variability. Facial injuries vary for 0.5% to 35% of all injuries. The highest frequency was found by Miller 2022 who investigated under-vehicle explosions in civilians. They also found more trunk injuries than other studies, probably due to the absence of protective clothes in the civilian population compared to the military population [37].

**Table 3:**
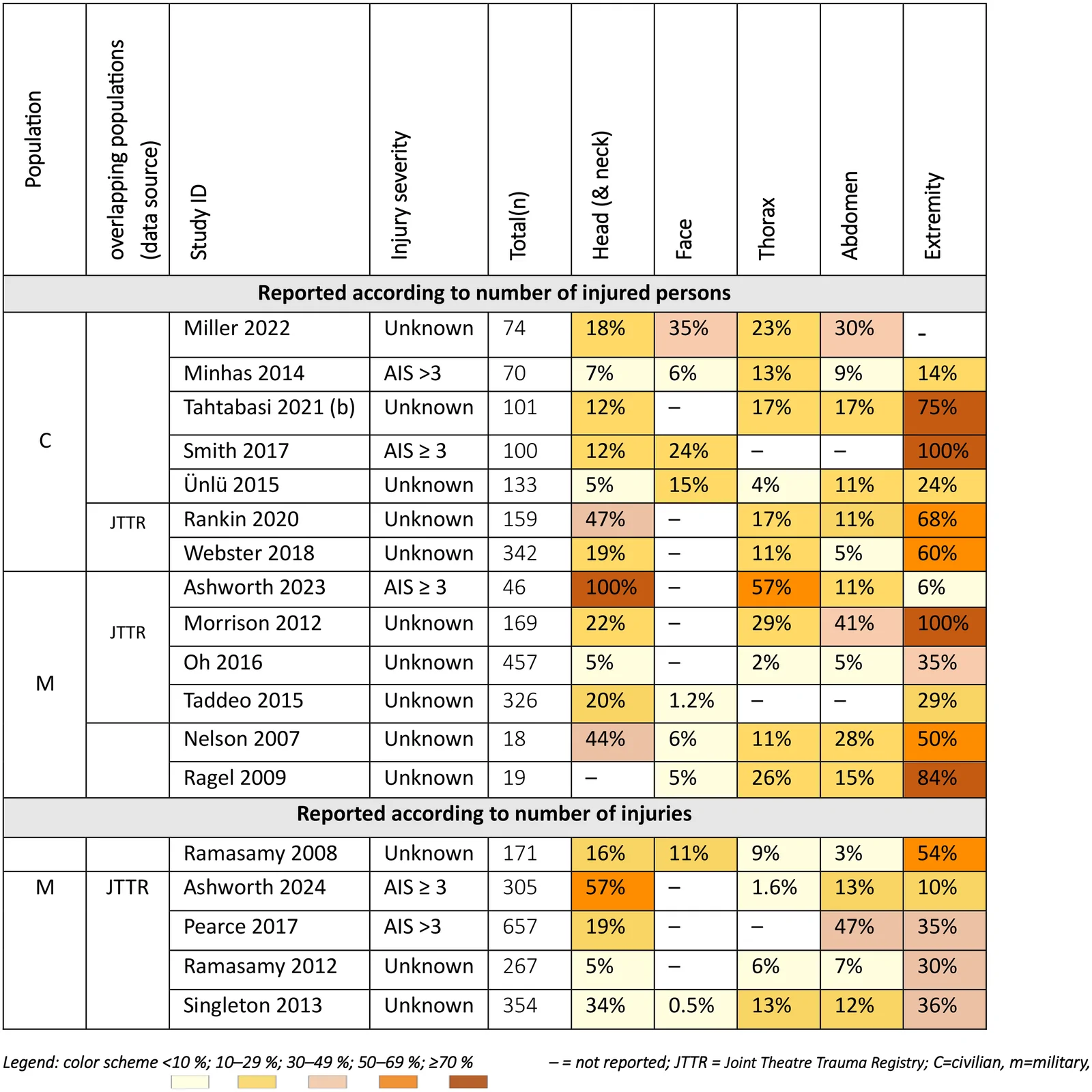
Heatmap distribution of injuries – IED

#### Injury pattern – focus on specific injuries

In the IED studies there was a dominate focus on extremity, pelvic, spinal injuries and amputations. Some results of studies with a focus on specific injuries or rather body regions are highlighted here for a broadened understanding of the injury patterns reported in the studies. For more details consult Additional File 4.

### Amputations

Cross 2014 found a high incidence of concurrent pelvic fractures in traumatic amputees, with an even higher incidence in those with bilateral amputations above the knee. Casualties with pelvic injuries mostly suffered a combined mechanism of antero-posterior compression and vertical shear injuries [45]. Dupaix 2018, on the other hand, reported an association of multiple extremity injuries with penetrating pelvic and abdominal trauma, urogenital injuries and upper extremity injuries in dismounted military patrols in Afghanistan. They generally noted a high number of multiple extremity injuries and discussed that this could be related to the population of mainly marine patrols who are mainly injured while dismounted. They demonstrated an association between single lower leg amputation and upper extremity injuries [46]. Penn-Barrell 2014 reported exclusively on bilateral and triple amputations and showed that in their cohort of 43 UK servicemen, nine also sustained open pelvic fractures. Additionally, 19 patients had urogenital tract injuries. Nine patients had a triple amputation of two lower extremities and one upper extremity (trans-radial or trans-humeral). No patient with four-limb amputations survived in their cohort [36].

### Pelvic injuries

The three studies on pelvic fractures demonstrate a wide range of different pelvic fractures (antero-posterior compression, lateral compression, vertical shear as well as other fractures) – also combinations were reported [22, 32, 40]. Mossadegh 2012 supports the studies on extremity injuries that in Iraq and Afghanistan, high numbers of combined injuries of high transfemoral amputations, pelvic injuries, and (maiming) perineal (urogenital) injuries occurred [40]. Webster 2019 shows that dismounted casualties are most often suffering unstable pelvic fractures in combination with traumatic amputations of which they often die due to exsanguination. They proposed three possible mechanisms: (a) axial load via the femoral head from directly standing on the IED, (b) flail of the lower limb, and (c) blast wind and fragmentation [22].

### Spinal injuries

Four studies investigated spinal injuries - three with mounted casualties and one study with dismounted military personnel [21, 30, 31, 47]. Spurrier 2015 reported on several different types of spinal fractures of the entire spine, including burst and Chance fractures (flexion distraction fractures). They showed that thoracic fractures often appear at T4–T6, whereas lumbar fractures were more common at L1. In general, lumbar fractures are very common in their cohort (47% of all spinal fractures) [21]. Freedman 2014 and Ragel 2009 analyzed thoracolumbar fractures [31, 47]. Freedman 2014 reported an increase in thoracolumbar burst fractures with/without spinal cord injuries, especially in relation to IED incidents in up-armored military vehicles. The authors stated that these injuries often occur in combination with other combat injuries [47]. Ragel 2009 focused on thoracolumbar Chance fractures due to hyperflexion of the thoracolumbar spine of vehicle occupants during an IED explosion. Two different injury mechanisms have been proposed: (a) sudden acceleration from below (similar to fighter jet ejection) and (b) sudden deceleration (similar to a helicopter crash) [31]. Spurrier 2015 added that cervical fractures are possible due to a head striking the inside of the vehicle or due to personal equipment being pushed upwards and strikes the head. They added that this is in line with the increasing risk of head injuries in those with C1 to C7 fractures [21]. Additionally, Taddeo 2015 showed that cervical fractures are rare in dismounted casualties [30].

### Head & neck injuries

Two studies exclusively investigated head and neck/spinal injuries due to underbody blast in fatalities of military personnel deployed in Afghanistan and Iraq [29, 48]. Stewart 2018 found that skull, neck and spine are vulnerable to tertiary blast forces which are related to injuries caused by flexion/extension, rotation, and shear [29]. Ashworth 2023 further found eggshell skull fracture related to high energy direct impact to the skull and Stewart 2018 add that high energy head injuries are most common in mounted fatalities, but these fatal head injuries do not seem to be mechanically related to the observed cervical spine injuries [29, 48].

## Multiple & unspecified explosive devices

### Characteristics of included studies

A total of 37 studies with 24,600 casualties were included in the category of multiple and unspecified explosive devices [1, 3, 5, 53–86]. It encompasses studies that included more than one specified explosive device such as mines, rockets, and IEDs as well as studies that did not define the explosive device that caused the explosion. These studies typically discussed blast injuries or explosions. More details regarding the explosive devices investigated in these studies are provided in Additional File 6. The data sources of these studies were mostly registries or medical data (including autopsy reports). Four studies reported on incidents that occurred prior to the 2000s in Northern Ireland and on the Falkland Islands [63, 64, 74]. All other studies investigated incidents after 2000. Most report on incidents in Afghanistan and/or Iraq (*n* = 17) [1, 3, 53, 54, 57–62, 67, 76, 77, 79, 80, 83, 86]. The mean age across 16 studies, reporting mean age, was 25.7 years [5, 55, 60, 62, 66, 68–70, 72, 73, 77, 79, 82, 86]. 85.8% of casualties were men (of 23 studies with a total of 16,062 casualties that reported sex). Two studies reported injuries in children [71, 83]. Sixteen studies investigated civilians exclusively, five had mixed populations and the rest had military populations. A special focus was on extremity and musculoskeletal injuries in four studies [53, 54, 73, 77], amputations in four more studies [62–65], abdominal injuries in three studies [60, 68, 84], head injuries in three studies [55], and burn injuries in one study [67]. Further details are provided in Table 4.

**Table 4:** Study characteristics multiple and unspecified explosive devices

Study ID	N	Age in years	**Sex**^**b**^	Country	Time frame	**Context**^**a**^	Focus of the study	JBI
Bashir 2013 [55]	16	Ø 29 (range 4–58)	87.5%	Pakistan	-	C	Head injuries	10
Belmont 2011 [54]	142	-	-	Iraq	2007 – 2008	M	Musculoskeletal trauma	9
Belmont 2013 [53]	4,563	-	-	Afghanistan & Iraq	January 1, 2006 – December 31, 2009	M	Musculoskeletal injuries	10
Bilukha 2011 [56]	307	55% <18	69.4%	Nepal	July 2006 - June 2010	C	Injury pattern of fatalities	10
Breeze 2011 (a) [58]	60	-	-	Afghanistan & Iraq	January 1, 2004 – September 30, 2009	M	Mandibular fractures	8
Breeze 2011(b) [57]	652	-	-	Afghanistan & Iraq	January 1, 2006– December 31, 2009	M	Ear injuries	8
Brewster 2021 [59]	99	-	-	Afghanistan	2012	M	General Injury pattern	9
Cardi 2019 [60]	267	Ø 19.2 (range 10–28)	41.2%	Afghanistan	January 2006 - December 2016	C	Abdominal injuries	10
Edwards 2012 [61]	4913	63% >21 y	87.7%	Afghanistan & Iraq	2002 – 2010	C	General injury pattern	9
Fleming 2012 [62]	109	Ø 23.2 (range 18–39)	100%	Afghanistan & Iraq	December 2007 - September 2010	M	Extremity amputation	10
Hull 1992 [9]	29	-	100%	Falkland Islands	1979 –1990	M	Extremity amputations	9
Hull 1994 [64]	34	-	-	Northern Ireland	December 1987 - January 1992	C & M	Extremity amputations	8
Hull 1996 [65]	100	-	-	Northern Ireland	December 1987 - January 1992	C & M	Pelvic injuries	7
Kamauzaman 2007 [66]	4	Ø 32 (range30-34	100%	Malaysia	2000	M	General injury pattern	8
Kauvar 2006 [67]	142	Ø 26 (±7)	-	Afghanistan & Iraq	April 2003 - April 2005	M	Burns	9
Khan 2015 [69]	262	Ø 31 (±14)	84%	Pakistan	January 2008 - December 2012	C	Soft tissue & skeletal injuries	10
Khan 2017 [68]	56	Ø 28 (±8.3)	91.1%	Pakistan	April 2012 - June 2015	C	Abdominal injuries	10
Korkmaz 2022 [70]	74	Ø 9.19 (±4.5)	69%	Syria	2015 – 2020	C	General injury pattern	10
Korkmaz 2023a [71]	36	range 1–15	69.4%	Syria	2011–2020	C	Blast lung injuries	9
Korkmaz 2023b [72]	65	Ø 9.6 (±4.3)	70.8%	Syria	2011–2022	C	Secondary blast injuries	10
Luria 2013 [73]	72	Ø 27.3 (±12.3)	43%	Israel	October 2000 - February 2004	C	Upper extremity	10
Mellor 1992 [74]	828	-	-	North Ireland	1970 – 1984	M	General injury pattern	9
Owens 2008 [3]	1,146	-	-	Afghanistan & Iraq	October 2001 - January 2005	M	General injury pattern	10
Peleg 2004 [75]	623	15–29: 297 (49%)	62.9%	Israel	October 1, 2000 – June 30, 2002	C & M	General injury pattern	10
Popivanov 2014 [1]	55	Ø 23.6 (range 5–70)	92.7%	Afghanistan	September - December 2010 & January - July 2010	C	General injury pattern	10
Ramasamy 2009 [76]	62	-	-	Iraq	January 1, 2006 – October 1 2006	C & M	General injury pattern	10
Ramasamy 2011 [77]	30	Ø 26.2 (±5.7)	96.7%	Afghanistan & Iraq	January 2006 - December 2008	M	Calcaneus fractures – deck flat injuries	9
Ritenour 2010 [5]	4,765	Ø 26 (±7)	97%	Worldwide	2003 – 2006	M	Primary blast injuries	8
Rozenfeld 2023 [78]	2,533	-	78.8%	Israel	January 1997 - December 2018	C&M	General injury pattern	9
Sargent 2024 [79]	100	Ø 10 (range 6–12)	-	Afghanistan	2011	C	General injury pattern	10
Sayer 2008 [80]	106	-	-	Afghanistan & Iraq	October 2001 - January 2006	M	General injury pattern	9
Shakargy 2022 [81]	222	Mdn 22 (IQR 20.26)	97.7%	Israel	1982 & 2021	M	Injury pattern among fatalities	9
Surani 2015 [82]	481	Ø 31.4 (±12.3)	96.3%	Pakistan	January 2000 - October 2007	C	External injuries	8
Thompson 2017 [83]	295	Mdn M 12 Mdn F 8	76.3%	Afghanistan	June 2006 - March 2013	C	General injury pattern	8
Wani 2009 [84]	154	-	80.5%	Pakistan	1998 – 2008	C	Abdominal injuries	7
Weil 2011 [85]	694	53% 15–29	65.9%	Israel	October 2000 - December 2005	C	Penetrating & orthopedic trauma	8
Wordsworth 2017 [86]	504	Ø 26	-	Afghanistan	April 1, 2006 - March 1, 2013	M	Facial trauma	8

***Legend:*** ^a^context: C = civilian, *M* = military, C&*M* = mixed population of civilians and military personnel; ^**b**^ Sex: percentage of male victims

JBI = Joanne Briggs Institute; Ø = mean; study ID = study identification; - = not reported

### Issues with reporting & risk of bias

Risk of bias assessment showed a low risk of bias, with a median of 9 out of 10 (Range 7–10, lower risk- higher score). Some of the included studies did not report explosives but rather stated that they investigated blast injuries or injuries due to an explosion. Therefore, there was considerable heterogeneity between the studies and variations in the injury patterns shown in the included studies. Some of the included studies did not provide details on the injuries suffered, but instead just reported the body regions affected; for example, Bilukha 2011 reported injured body regions by dividing the body into upper and lower body [56]. Age and sex were reported inconsistently. Only 23 studies provided age or sex distribution. When children were included in the overall population, the study did not report differences in injury patterns by age groups.

## General injury pattern

**Fig. 4:**
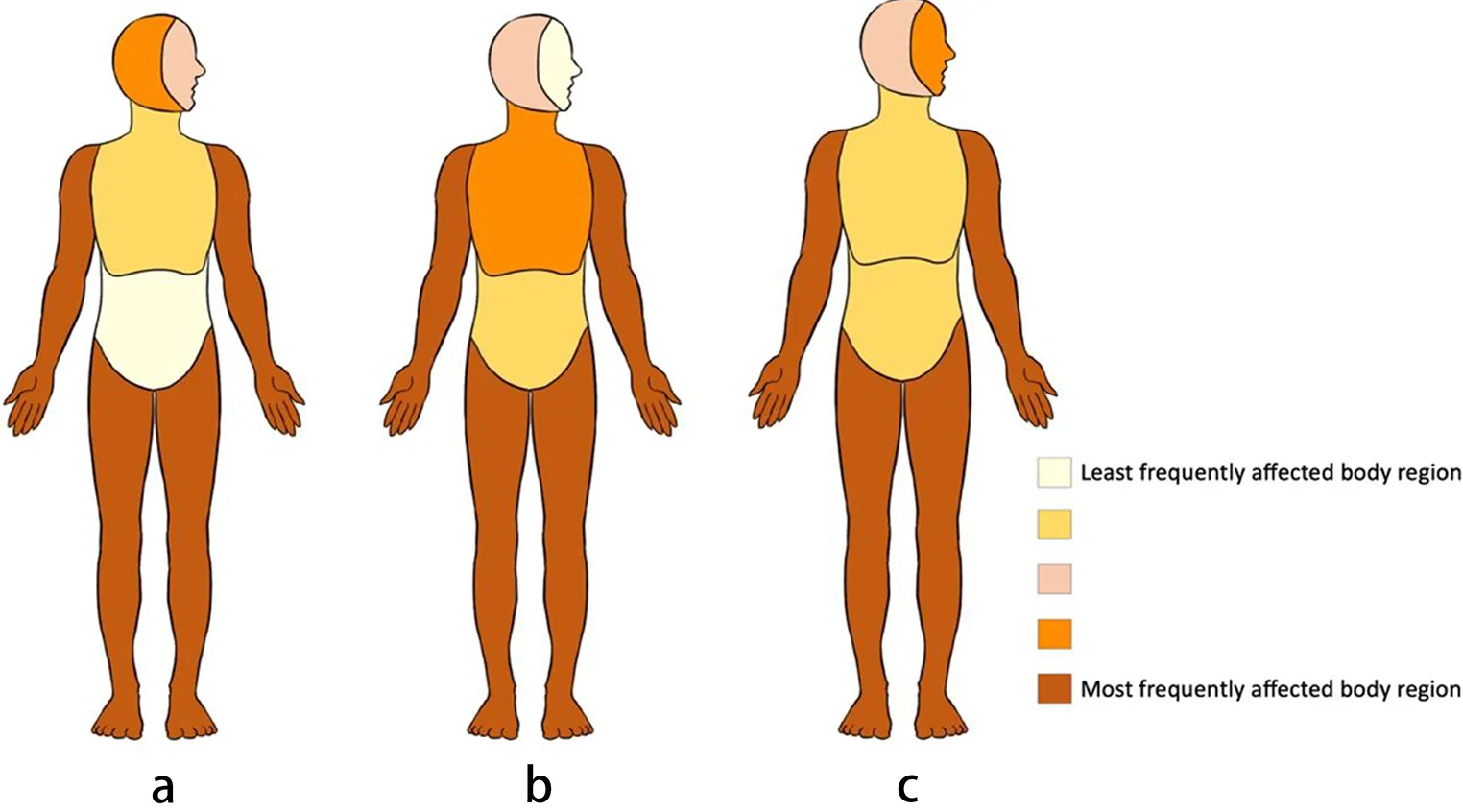
**a**) Civil and military, **b**) Civil population, **c**) Military population

Sixteen of the 33 studies reported all or nearly all AIS regions (head, face, thorax, abdomen, and extremities). Thirteen of these were included in the analysis of general injury patterns [1, 9, 59, 62, 64, 66, 69, 73–77, 80]. The remaining three were not included due to differential reporting of AIS severity level (AIS < 3 & >3), whereas the analyzed studies did not state the severity of the reported injuries [61, 81, 83]. The ranking of injury regions shows that in the total population (civil & military) extremities were most frequently injured, followed by head and abdominal injuries, which were the least frequent (Fig. 4a). For military populations (Fig. 4c), the face was the second most common injury region, followed by the head [9, 59, 62, 66, 74, 77, 80], whereas in civilians (Fig. 4b), the second most common injury region was the thoracic region, followed by the head. The face was the least commonly injured body region [1, 69, 73]. Compared with the three studies that reported injuries with AIS ≥ 3. Extremities injuries were the most common, but followed by head and thorax (both ranked 2), and abdomen and face (least common).

The heatmap in Table 5 shows that head injuries are common in military populations (Owens 2008:88%, Wordsworth 2017:43%, Mellor 1992:32%). Owens 2008 summed a range of different explosive devices ranging from IED, landmine, mortar or shrapnel, bomb, and grenade into one category and reported head and facial injuries together, which may account for the high proportion of head injuries in comparison to other studies [3]. In civilians, proportions vary greatly among studies (4–40%). Higher rates are reported by Popivanov 2013 who described causalities treated by Bulgarian surgeons in July 2010 in Role 2 Kandahar Regional Military Hospital Afghanistan [1].

**Table 5:**
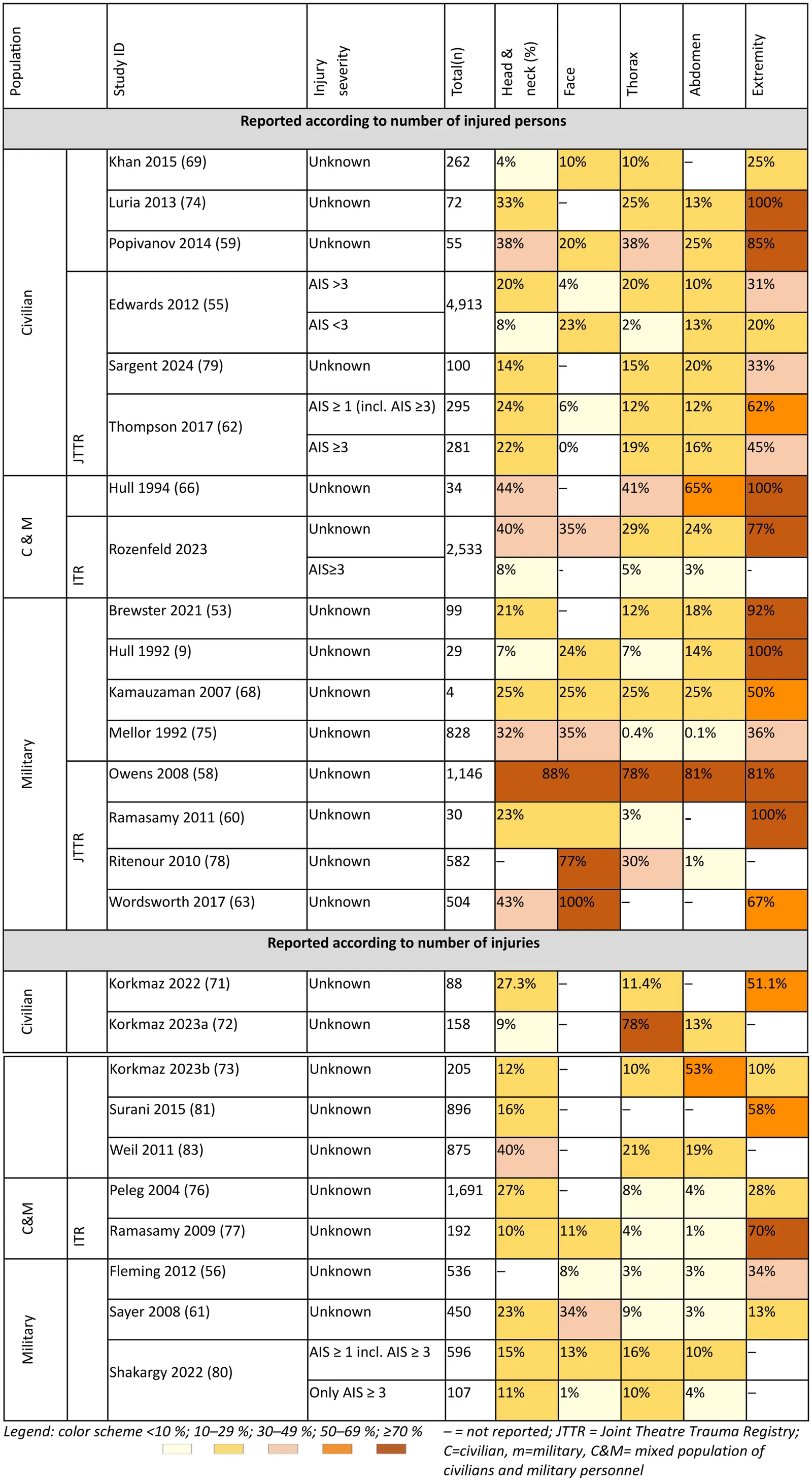
Heatmap multiple & unspecific explosive devices

Facial injuries are highly heterogeneous (e.g., Wordsworth 2017:100%, Ritenour 2010:77%, Mellor 1992:35%). Several studies, such as Wordsworth 2017, focused on facial injuries; hence, they included only facial injuries and recorded other injuries, whereas Ritenour 2010 investigated primary blast injuries, which probably has an increased proportion of facial (especially tympanic injuries) and thoracic injuries in this cohort [5, 86]. Further, also some military studies show a tendency to report higher proportions of thorax injuries (e.g., Owens 2008: 81%, Kamauzaman 2007: 25%, Korkmaz 2023a: 78%), whereas abdominal injuries are less frequently reported, generally below 25% [3, 66, 71]. However, some exceptions were found, such as in the study by Hull 1994, who reported 65% abdominal injuries in their study cohort. The authors focused on amputation injuries in fatalities and included injuries due to several explosives, such as a car bomb under the driver’s seat. This, along with the focus on fatalities, may have resulted in a higher proportion of abdominal wounds compared to other studies [64].

Extremity injuries as shown in Figs. 4 are the most frequently (>50%) reported injury region with some studies reporting very high rates e.g., Popivanov 2014 (85%), Brewster 2021 (92%), Hull 1992 (100%), Ramasamy 2009 (70%) [1, 59, 63, 76]. In military populations, the rates are higher, possibly due to protective equipment that secures the torso but leaves the extremities mostly exposed. Most studies which included military personnel mainly reported injuries to the lower extremity. Only Bemont 2011 showed more injuries to the upper extremities caused by explosions. One reason could be the different positioning of explosive devices (e.g., underground, in walls of buildings etc.), which was not further investigated by this study [54].

In three studies, only injured children were reported (Korkmaz 2022, Korkmaz 2023a, Thompson 2017). The distribution of injuries also shows that extremity injuries are the most common. The second most injured body region varied across studies. While Korkmaz 2022 reports more facial injuries, Korkmaz 2023a reported mainly on blast lung injuries and Thompson 2017 showed more abdominal and thoracal injuries [70, 71, 83].

### Injury pattern – focus on specific body parts

Sixteen included studies reported injuries only in relation to a specific body part [53–55, 73, 77, 86] or specific injuries [9, 57, 58, 60, 62, 64, 65, 67, 68, 84] as shown in Additional File 5.

#### Extremity injuries & amputations

Most often the focus was on extremity injuries [53, 54, 73, 77] and amputations [9, 62, 64, 65]. For example, Ramasamy 2011 exclusively investigated deck slap injuries in calcaneus fractures in military personnel due to under-vehicle mines. They found that extensive lower extremity injuries appeared frequently in combination with spinal injuries. Hence, these severe lower extremity injuries are part of complex multiple injuries across the body [77]. These findings are consistent with those of the IED category. Fleming 2012 found similar findings in their dismounted population as in the IED category – most of their casualties were harmed by IEDs. They observed that more than 18% of their cohort suffered from more than one extremity amputation with devastating injuries to the pelvis and urogenital organs [62]. Nonetheless, prior to the conflicts in Afghanistan and Iraq, studies of such injury combinations were observed, such as by Hull in 1994 in fatalities due to lorry or van bomb explosions in Northern Ireland [64]. Luria 2012 compared upper extremity injuries in civil casualties harmed by an explosion compared to gunshot victims in Israel. They showed that blast casualties suffer more often from bilateral upper-extremity injuries, burns, and penetrating injuries than those harmed by gunshots. Luria 2012 is also one of the rare studies who included more female than male casualties [73].

#### Facial injuries

Additionally, some of the studies investigated facial injuries – from primary blast injuries in Breeze 2011b to more general facial injuries in Wordsworth 2017 [57, 86]. The latter discuss that facial injuries are very common in modern conflicts, often combined with extremity injuries in mounted and dismounted populations. Most injuries occur in the midface or lower third of the face. They pointed out that in fatalities, the mandibular is most frequently fractured, whereas survivors frequently suffered from mid-facial fractures [86].

#### Abdominal injuries

Furthermore, three studies investigate abdominal injuries ranging from general overview of abdominal injuries in Khan 2017, to primary blast in Wani 2009 to penetrating abdominal injuries in Cardi 2019 [60, 68, 84]. Khan 2017 showed how complex and diverse abdominal injuries are due to explosions in their civil populations. Serious injuries may be present despite the absence of obvious abdominal wall injury [68]. This is in line with the findings of Wani 2017, who demonstrated frequent small bowel injuries but also injuries to solid organs. Mortality and morbidity associated with the proximity to the explosion and the number and severity of organ damage [84].

#### Burn injuries

In addition, Kauvar 2006 focused on burn injuries. They observed that the severity and frequency of burn injuries increased along with the proportion of combat explosions. They added that burns mostly appear in mounted populations and that two patterns are distinct [1]: small isolated burns to the hands and face in which the casualties sustained these burns while outside the vehicle and [2] larger body surface area burns to the trunk and lower extremity in casualties harmed in a smoldering fire of vehicles [67].

## Discussion

### IED

IEDs are primitive, often homemade explosive devices assembled from locally available resources or made of modified conventional ammunition and additional fillings, such as nails, which leads to unpredictable and exacerbated damage [87]. The term IED is often used in military literature since the Afghanistan and Iraq conflicts [36]; however, a clear distinction between terrorist attacks and IED is not possible (see studies from Nigeria in which IEDs are used in suicide bombing attacks [88, 89]). IEDs harm both military personnel civilians. Its use is so widespread that it has become a global threat [87].

The most commonly injured body region in the present study was the extremities in all investigated populations. Followed by head and abdominal injuries in military populations and thoracal and abdominal injuries in civilian populations. The study populations were predominantly young males under 40 years of age. In particular, studies on military populations describe very young casualties with a mean age of <30 years. IEDs often used in a military context, although not precisely defined, are characteristic of their injury pattern, especially when compared to antipersonnel landmines. This systematic review is in line with other studies demonstrating and confirming that IEDs show a far more extensive injury pattern, especially in dismounted casualties injury mechanisms [27, 36].

### Injury pattern in dismounted casualties

Injury pattern in dismounted casualties show that IEDs lead to amputations, often including several extremities (lower and upper), high femur amputations (e.g., hindquarter) and pelvic fractures, often with urogenital involvement. For example, Penn-Barrel 2014 shows results on bilateral amputation and discusses that these kind of injuries are more common in asymmetric wars in which typically less equipped perpetrators opt for weapons like IEDs instead of directly attacking the superiorly equipped forces [36], as also seen in other conflicts, for example the Turkey-Kurdish conflict [90]. Furthermore, Dupaix 2018 confirmed that the number of amputations, especially multiple amputations, increased dramatically during the so-called *Global War on Terror,* in which IEDs were a commonly used as weapons [46, 87]. Singleton 2013 pointed out that death often occurs as a consequence of exsanguination from junctional and extremity hemorrhage [26, 91]. Other studies on IEDs show consistently high mortality rates (not included in the current study), but as Nelson 2007 points out, due to improved protective equipment, increased air rescue, and closer proximity of Shock Trauma Platoon and Forward Resuscitative Surgery System, more soldiers are present in role 2 and receive advanced medical health care and hence, a higher probability of survival [41].

Several papers document the vulnerability of extremities due to lack of protective amor in military populations e.g., with lower-than-expected rate of abdominal, thoracic and eye injuries in causalities in Afghanistan [27, 92, 93]. Penn-Barrel 2014 adds that the left arm is more often amputated due to its exposed extended position in front of the body holding the personal weapon when patrolling on foot [36]. However, this cannot be confirmed or ruled out based on the present data. Furthermore, the present findings on civilian populations confirm the impact of protective armor as civilians demonstrate far more thoracic and abdominal injuries than military populations. Additionally, Ostfeld 2024 added that the impact of proximity to the explosive device influences the injury pattern [94]. Several of the included papers describe pelvic fractures, especially in dismounted casualties [32, 36, 40, 42]. Casualties, especially military personnel, often present with severe open pelvic fractures after (bilateral) lower extremity amputations, often combined with uncontrolled hemorrhage from large vessels and disastrous urogenital and pelvic floor injuries [42].

Males were predominantly included in the primary studies. However, the sex distribution was missing in several of the included papers, hindering the investigation of exact injuries, especially in relation to urogenital injuries. Nonetheless, most soldiers are still of male sex. Hence, injuries sustained by female soldiers are often not registered yet. Females may suffer different injuries; for example, urogenital injuries are common in IEDs, as described above, but female urogenital injuries are vastly unknown. Furthermore, it could be that their injuries are different due to differences in body structure, weight, and height. As shown by Reed 2018, renal injuries are predominant in female soldiers and other injuries to the pelvic region are infrequent in females, but this is also related to the fact that in their cohort, females were not involved in direct combat action. In their cohort, the vast majority of genital injuries were observed in male soldiers. However, with the increase of women serving in the military globally, the number of female soldiers harmed during current and future conflicts is likely to rise. Hence, it is of increasing importance to consider female anatomy and function in research and prevention strategies, such as the design of protective equipment, as well as in the training of surgeons [95].

Similarly, injuries in children are vastly unreported but they probably suffer different and more severe injuries than adults due to height, weight, and differences in body structure. For example, Smith 2017 included children and showed that 1 in 10 casualties in their cohort were children and that children suffered the severest injuries with nearly 90% of cases suffering multiple amputations. However, they did not report injury patterns separately for pediatric casualties, which would have substantially added to the existing evidence on pediatric injury patterns [26].

In military personnel, pelvic injuries are often described as anterior to posterior compression fractures, in which the upward force of the blast distracts the hemi-pelvis from the sacrum [91]. This assumes that the IED is buried underground, but the devices can also be placed in walls, which would change the direction of the explosive charge [45]. However, the two studies on mixed study populations (military and civilian causalities) do not describe a difference in pelvic injuries between civilians and military personnel [43, 45]. Cross 2014 described that the classification of these pelvic fractures is complex because of the combined injury mechanism. Hence, most studies describe Tile A to C injuries and soft tissue injuries to at least one Faringer zone but often casualties suffer injuries to all three Faringer zones [32, 40, 42]. Regarding soft tissue injuries, Ramasamy 2012 pointed out that pelvic injuries sustained in a military setting often suffer massive soft tissue injuries and that wounds are often highly contaminated with soil, blast fragments, and clothing [32]. Hence, early mortality is often due to hemorrhage, and inability to control the source of bleeding, whereas late mortality is highly associated with infection and sepsis due to wounds contamination [40, 42]. However, pelvic injuries due to IED blasts are mostly penetrating and not blunt, as is common in civilian settings not related to explosions.

### Injury pattern in mounted casualties

Furthermore, head and spinal injuries are common in mounted military populations. Ashworth 2023 and Singleton 2013 showed that death in mounted casualtiesis often related to head injuries (e.g., eggshell fractures related to the direct impact of the car cabin ceiling to the head) [26, 48], whereas Spurrier 2015 and Freedman 2014 discuss spinal injuries in survivors [21, 47]. Freedman 2014 discussed that lumbar burst fractures are common in mounted casualties. The blast energy from under-vehicle detonations moves vertically into the vehicle cabin and causes in first instance fractures at impact contact points such as the calcaneus or pelvis. The thoracolumbar spine absorbs the vertical load, causing spinal burst fractures. In some instances, this is followed by the soldier hitting his head on the vehicle ceiling or rapid acceleration/deceleration associated with head and spinal fractures [47]. In addition, Ashworth 2023 showed the association between spinal and head injuries in mounted casualties. The authors described four different types of injury constellations: “[1] multiple spinal injuries in addition to skull fractures and brainstem injuries [2], peri-mesencephalic hemorrhage [3], spinal and brainstem injuries, and [4] contusions with injuries to C0–C1” [48].

Several studies have pointed out that head and spinal injuries can be prevented or at least reduced in severity by vehicle design and protective equipment such as helmets (Stewart 2018 & Ashworth 2023). Additionally, Freedman 2014 reported that improved vehicle technology has already increased the survival in mounted casualties, but resulted in an increase in thoracolumbar burst fractures [47]. However, Stewart 2018 showed that blast injuries in mounted casualties are commonly complex and unpredictable in nature, owing to the position of individuals within the vehicle, size of the explosive device, and open or closed deck vehicles. This complicates strategies for mitigating injuries in military personnel [29].

However, injuries to the head and thorax vary in frequency more widely than extremity injuries. Civilians generally show a broader variability in injury patterns, probably also due to the absence of protective clothing and proximity to the explosive device.

### Multiple und unspecified explosive devices

The injury patterns from multiple and unspecified explosive devices are highly heterogeneous. In civilians, the most commonly injured body regions are the extremities, thorax, and head, but several studies have reported higher proportions of thoracic injuries (e.g., Korkmaz 2023a, who focused on blast lung injuries [71]) and seldom higher frequencies of abdominal injuries (see Hull 1994 [64]). In military populations, the distribution of injuries is mostly restricted to injuries to the extremities, face, and head, probably due to protective equipment, as also explained in the section on injuries due to IEDs. The diffuse pattern of injuries is partly caused by the multitude of explosive devices included in the primary studies, such as rockets, mines, and car bombs, which explode in different proximations. A precise description of the blast event is commonly missing, which makes an analysis of the factors influencing the injury pattern impossible. However, differences in pattern could, for example, be caused by variances in the positioning of the casualties in relation to the explosive device during the event (e.g., sitting, standing) or the location, whether an explosive device detonated in an open versus a confined space.

Several studies have focused on primary blast injuries, which may have influenced the frequency of facial injuries in this category, as tympanic injuries are very common primary blast injuries (see e.g., Ritenour 2010 [5]). Furthermore, few studies have explored exclusively injuries in fatalities. These studies often show deviating injury patterns (e.g., Hull 1994 with higher frequencies of abdominal injuries compared to studies on survivors [64]). This indicates that the injury patterns in survivors may be vastly different from those KIA/DOS. In all other included studies, injuries were mostly reported in survivors, and rarely in those who died of wounds. Descriptions of those KIA/DOS were in most cases only available for studies on terror attacks or suicide bombings, but often incomplete.)., Studies on blast injuries in military personnel (IEDs, multiple explosive devices) rarely reported KIA/DOS because not all registers collect data on KIA/DOS, which hindered an analysis of differences in injury patterns between fatalities and survivors.

Furthermore, age is inconsistently reported. In studies with multiple age groups, differences in injuries among these groups are not highlighted. Only some of the included studies focused exclusively on children. Thompson 2017 showed children frequently suffer extremity injuries, but head injuries are more common in fatalities. They described more severe head and neck injuries in young children than in older children. The authors hypothesized that children suffer more frequently from head injuries than adults [83].

The data suggest that the type of injury depends heavily on the context (civilian vs. military), severity (AIS), and protective equipment. Overall, extremity and head injuries were the most common, which suggesting the need for targeted medical preparation and care, especially for military personnel, and in regard to orthopedic and neurosurgical specialties.

### Comparison to current conflicts

Currently, only a few studies have investigated injuries from current conflicts, such as Gaza-Israel and Ukraine [96–100]. While specific details about the explosion mechanisms, as well as the location and position of casualties at the time of explosion, are not (yet) extensively reported, the injury patterns of blast injuries described in these studies are consistent with our review findings. Recent studies have indicated that mixed populations with a higher proportion of civilians are affected [99, 100]. The injury pattern shown in the current study is similar to the injuries described by Lawry 2025. The authors describe the injury patterns in Ukrainian military personnel and state that the injuries to extremities, face, neck, and head are the most frequent. Similarly, Fogel 2025 showed with data from the Gaza-Israel conflict that extremities are the most frequently injured body region with a lower frequency of thoracic and head injuries in line with the use of protective equipment. Hence, Fogel 2025 argued that there is a need for reallocation of resources towards trauma and orthopedic specialties to improve training and increase the workforce to address the predominance of extremity injuries due to modern conflicts [98]. However, Lawry 2025 showed that the types of wounds were more extensive than those described in studies investigating the conflicts in Afghanistan and Iraq. Additionally, newer weapons cause an increase in casualties in shorter time periods and diverting injury patterns. For example, the use of thermobaric explosives is related to an increase in burn, traumatic brain, and lung injuries [97].

## Limitations

Table 6 provides an overview of terminological and definitional issues by body region. These issues influenced the analysis and hindered more profound statistical analysis. However, many of the included studies did not consistently report injuries across the body. Hence, it was often left unclear whether the casualties did not have injuries in a specific body region or whether they did not report every single body region/injury. Additionally, the severity of the injuries was mostly unknown. It is unclear whether only severe injuries (e.g., AIS > 2) were reported or whether some studies reported all injuries including lacerations and abrasions. Hence, for example, in extremity injuries, it is therefore often unclear whether the reported injuries describe serious injuries such as fractures or simple scratches. Hence, there is a need for more standardized reporting of blast injuries. However, this standardized and complete documentation is probably only possible by digitalization of record keeping – supportive and intuitive systems that support surgeons in recording injuries, especially in roles 2 and 3, without a high level of bureaucratic documentation issues. Dealing with blast injuries (survival and outcome) also depends on the timely availability of prehospital care, established and possible evacuation pathways, protection gear, and (delay) time to (definitive) care.

**Table 6:** Overview of terminologies and definition issues by body region

Head/neck	In several studies, it is unclear whether neck injuries were included in cervical spine injuries. Additionally, it is left unclear which injuries are reported as cervical spine injuries and which as neck injuries.
Pelvis	In several publications pelvis could have been reported as part of extremity or abdominal injuries.
Spine	In several publications spine could have been reported as part of head/neck, thorax and abdominal injuries.
Urogenital	In several publications urogenital injuries could have been reported as part of abdominal injuries.
External injuries	In several studies the definition of external injuries was uncertain. External injuries were frequently left out in reporting. Often external injuries were only reported in burn injured patients, or external injuries were used as an umbrella term for abrasions, lacerations, burn and other injuries. Hence, it is in most studies uncertain whether amputations are included in extremity injuries or are counted separately as an own entity.
Mortality	• Total numbers mostly unclear. • Difference between killed in action/died on scene and died of wounds not described. • Often only those died of wounds reported →total mortality of one blast event unknown. • Only few results on those killed in action/died on scene.

## Conclusion

The results of this systematic review show the multidimensional characteristics of blast injuries. Two categories were explored in more detail in this part 1 to show the difference in injury patterns by explosive device: IEDs and multiple/unspecific explosive devices. Whereas IED injuries in dismounted casualties are often characterized by (high) amputation injuries with pelvic and urogenital injuries combined with drastic soft tissue injuries in dismounted casualties and head, spine, and foot injuries in mounted military casualties, the blast injury pattern due to multiple or unspecified explosives is much more diffuse. This is partly caused by the multitude of explosive devices in the included studies, such as rockets, mines and car bombs which explode in different proximations, and by the position of the casualties in relation to the explosive device (e.g., sitting, standing) and location (open versus closed space) in this category. Nonetheless, especially regarding the most common injury regions in civilians, similar injury patterns are reported in the IED category (most common extremities, thorax & abdomen) and in the multiple/unspecific explosive device category (most common extremities, thorax and head).

However, blast injuries are often a unique combination of injuries caused by blast-waves, fragments and explosion by-products, resulting in penetrating and blunt as well as burn injuries (thermal and chemical) commonly seen in a single patient. Furthermore, the resulting wounds are frequently highly contaminated with soils, fragments and explosive by-products. Therefore, these patients suffer from severe and complex traumata, demanding detailed specialized knowledge and highly sophisticated coordination of resources [101, 102].

## Electronic supplementary material

Below is the link to the electronic supplementary material.

Supplementary Material 1

Supplementary Material 2

Supplementary Material 3

Supplementary Material 4

Supplementary Material 5

## Data Availability

All data generated or analyzed during this study are included in this published article and its supplemental information files.

## Abbreviations

AIS: Abbreviated Injury scale
C: Civil
C & M: Civil & Military
IED: Improvised explosive devices
ICRC: International Committee of the Red Cross
ISS: Injury Severity Score
ITR: Israel National Trauma Register
JBI: Joanna Briggs Institute
JTTR: Joint Theater Trauma Registry
M: Military
Mdn: Median
UXO: Unexploded Ordnance
