# Supplementary material for: Pattern of blast injuries. A Systematic Review: Part 1–Improvised, multiple and unspecified explosive devices
Source: Eur J Trauma Emerg Surg. 2026 Aug 24;52(1):266. doi: 10.1007/s00068-026-03261-x (PMC13503492; doi:10.1007/s00068-026-03261-x)
Supplement: Supplementary file 1 [file 68_2026_3261_MOESM1_ESM.docx]

# **Additional file 2 – search strategy**

Medline via Pubmed search

1. (blast[Title/Abstract]) OR (agents, explosive[MeSH Terms])
2. injur*[Title/Abstract]
3. #1 AND #2
4. blast injuries[MeSH Terms]
5. #3 OR #4
6. pattern*[Title/Abstract]
7. #5 AND #6

🡪 Search on 5.1. 2023 – 585 HITS

-> Search Update on 26.03.2025 653 hits

Scopus Search

1. TITLE-ABS-KEY  “b*last*  injury”
2. TITLE-ABS-KEY “Blast inuries”
3. TITLE-ABS-KEY *"explosive agents"*  OR  *blast*
4. #1 OR #2 OR #3
5. TITLE-ABS-KEY ( *pattern* )
6. #4 AND #5

🡪 Search on 5.1. 2023 - 749 Hits

Plus 21 hits from Secondary Documents

--> Search Update on 26.03.2025 - 749 Hits
