# Supplementary material for: Pattern of blast injuries. A Systematic Review: Part 1–Improvised, multiple and unspecified explosive devices
Source: Eur J Trauma Emerg Surg. 2026 Aug 24;52(1):266. doi: 10.1007/s00068-026-03261-x (PMC13503492; doi:10.1007/s00068-026-03261-x)
Supplement: Supplementary file 2 [file 68_2026_3261_MOESM2_ESM.docx]

# **Appendix 2 – excluded full text**

| Study ID | Reason for exclusion |
| --- | --- |
| Adhikari 2013 (1) | Not in relation to armed conflict or terror attack |
| Aharonson-Daniel 2006 (2) | No injury pattern |
| Akhavan-Sigari 2018 (3) | Wrong patient population |
| Al Issa 2008 (4) | Untraceable publication |
| Al-Azzawi 2004 (5) | Wrong population |
| Appenzeller 2004 (6) | Mix of Blast and gunshot |
| Arora 2009 (7) | Wrong patient population |
| Arul 2012 (8) | Mix of Blast and gunshot |
| Barzilai 2015 (9) | Focus on surgical management |
| Belmont 2012 (10) | Not about blast injury pattern |
| Bhandarwar 2012 (11) | Mix of Blast and gunshot |
| Boffard 1993 (12) | Wrong study design |
| Born 2007 (13) | Wrong study design |
| Boutonnet 2011 (14) | Wrong study design |
| Bowley 2013 (15) | Wrong study design |
| Breeze 2015 (16) | Wrong intervention |
| Carley 1997 (17) | Focus on surgical management |
| Caro 1973 (18) | Focus on surgical management |
| Centers for Disease Control 2015 (19) | Wrong study design |
| Chaiphrom 2009 (20) | Untraceable publication |
| Chan 2012 (21) | Mix of Blast and gunshot |
| Chianakwana 2017 (22) | Wrong patient population |
| Christensen 2012 (23) | Animal study |
| Cooper 1983 (24) | Wrong study design |
| Covey 2010 (25) | Wrong study design |
| Daw 2019 (26) | Wrong study design |
| Delannoy 2019 (27) | Wrong patient population |
| Delannoy 2020 (28) | Not about blast injury pattern |
| Dootz 2021(29) | Mix of Blast and gunshot |
| Doucet 2011 (30) | Mix of Blast and gunshot |
| Dunn 2021 (31) | Mix of Blast and gunshot |
| Dunn 2016 (32) | Mix of Blast and gunshot |
| Dussault 2016 (33) | Wrong study design |
| Dussault 2014 (34) | Wrong study design |
| Eardley 2012 (35) | Mix of Blast and gunshot |
| Eastridge 2017 (36) | Wrong study design |
| Edirisinghe 2015 (37) | Wrong study design |
| Farhat 2024 (38) | Mix of Blast and gunshot |
| Feenstra 1986 (39) | Not in relation to armed conflict or terror attack |
| Fogel 2025 (40) | Mix of Blast and gunshot |
| Fox 2006 (41) | Wrong patient population |
| Gataa 2011 (42) | Mix of Blast and gunshot |
| Giessler 2006 (43) | Not in relation to armed conflict or terror attack |
| Goksel 2005 (44) | Wrong study design |
| Gordon 2018 (45) | Wrong study design |
| Gurney 2020 (46) | Wrong study design |
| Hahn 1996 (47) | Not in relation to armed conflict or terror attack |
| Hazani 2009 (48) | Not in relation to armed conflict or terror attack |
| Helling 2004 (49) | Wrong intervention |
| Helou 2024 (50) | Focus on logistic /health care response |
| Hesling 2022 (51) | Mix of Blast and gunshot |
| Hodge 2008 (52) | Untraceable publication |
| Hoencamp 2014 (53) | Mix of Blast and gunshot |
| Hoencamp 2014 (54) | Mix of Blast and gunshot |
| Hoencamp 2015 (55) | Mix of Blast and gunshot |
| Hudak 2005 (56) | Wrong study design |
| Hull 1992 (57) | Wrong study design |
| Ianov 2001 (58) | Wrong language |
| Islinger 2000 (59) | Mix of Blast and gunshot |
| Jacobs 2012 (60) | Focus on logistic /health care response |
| Jahangiri 2018 (61) | Not in relation to armed conflict or terror attack |
| Karmy-Jones 1994 (62) | Wrong study design |
| Khosravi 2015 (63) | Wrong patient population |
| Khurana 2011 (64) | Wrong study design |
| Khurana 2012 (65) | Wrong study design |
| Kittle 2012 (66) | Mix of Blast and gunshot |
| Kluger 2004 (67) | Focus on logistic /health care response |
| Kluger 1997 (68) | Wrong language |
| Kosashvili 2009 (69) | Focus on logistic /health care response |
| Krishtafor 2019 (70) | Wrong language |
| Kuckelman 2018 (71) | Wrong study design |
| Lafta 2015 (72) | Mix of Blast and gunshot |
| Langworthy 2002 (73) | Untraceable publication |
| Lawry 2025 (74) | Focus on logistic /health care response |
| Lechner 2010 (75) | Wrong study design |
| Machaku 2023 (76) | Not in relation to armed conflict or terror attack |
| Malie 2012 (77) | Wrong study design |
| Mamczak 2013 (78) | Focus on surgical management |
| Maqsood 2015 (79) | Mix of Blast and gunshot |
| Master 2015 (80) | Untraceable publication |
| Mayo 2006 (81) | Wrong study design |
| McLean 2006 (82) | Wrong study design |
| McMenemy 2021 (83) | Wrong patient population |
| Mohammad 2018 (84) | Wrong patient population |
| Molaie 2020 (85) | Wrong study design |
| Morley 2007 (86) | Wrong study design |
| Mühlmeier 2023 (87) | Wrong study design |
| Nor 2023 (88) | Mix of Blast and gunshot |
| Nunziato 2021 (89) | Not in relation to armed conflict or terror attack |
| Ostfeld 2024 (90) | Not about blast injury pattern |
| Parker 2023 (91) | Mix of Blast and gunshot |
| Paul 2008 (92) | Wrong study design |
| Paydar 2012 (93) | Focus on surgical management |
| Peleg 2003 (94) | Mix of Blast and gunshot |
| Peramaki 2011 (95) | Wrong study design |
| Pillgram-Larsen 1992 (96) | Wrong language |
| Poon 2013 (97) | Focus on surgical management |
| Powers 2019 (98) | Wrong study design |
| Powers 2013 (99) | Wrong study design |
| Pronk 2016 (100) | Wrong study design |
| Rafaels 2013 (101) | Wrong study design |
| Razaq 2015 (102) | Mix of Blast and gunshot |
| Rice 2000 (103) | Untraceable publication |
| Ruwanpura 2008 (104) | Wrong patient population |
| Saravanapavananthan 1978 (105) | Wrong patient population |
| Schmidbauer 2010 (106) | Wrong study design |
| Schoenfeld 2013 (107) | Wrong patient population |
| Schwartz 2014 (108) | Mix of Blast and gunshot |
| Shankar 2016 (109) | Wrong study design |
| Singh 2016 (110) | Wrong study design |
| Sirko 2025 (111) | Mix of Blast and gunshot |
| Sorkin 2004 (112) | Wrong intervention |
| Stemper 2011 (113) | Wrong study design |
| Stevenson 2009 (114) | Wrong study design |
| Tekade 2017 (115) | Mixed blast and gunshot |
| Tin 2021 (116) | Not about blast injury pattern |
| Tintle 2010 (117) | Wrong study design |
| Tovar 2021 (118) | Wrong study design |
| Turégano-Fuentes 2014 (119) | Wrong study design |
| Van der Linden 2017 (120) | wrong language |
| Webster 2015 (121) | Wrong study design |
| Wightman 2001 (122) | Wrong study design |
| Willits 2015 (123) | Wrong study design |
| Wold 2021 (124) | Mix of Blast and gunshot |
| Yunusa 2018 (125) | Mix of Blast and gunshot |
| Zhang 1996 (126) | Animal study |

References:

1.	Adhikari S, Bandyopadhyay T, Sarkar T, Saha JK. Blast injuries to the hand: Pathomechanics, patterns and treatment. J Emerg Trauma Shock [Internet]. 2013;6(1):29–36. Available from: https://www.scopus.com/inward/record.uri?eid=2-s2.0-84873572612&doi=10.4103%2f0974-2700.106322&partnerID=40&md5=a7db10dd3833953bd6b2efa338829fff

2.	Aharonson-Daniel L, Klein Y, Peleg K. Suicide Bombers Form a New Injury Profile: Ann Surg [Internet]. 2006 Dec [cited 2024 Sep 4];244(6):1018–23. Available from: http://journals.lww.com/00000658-200612000-00023

3.	Akhavan-Sigari R, Mielke D, Farhadi A, Rohde V. Study of radial nerve injury caused by gunshot wounds and explosive injuries among Iraqi soldiers. Open Access Maced J Med Sci [Internet]. 2018;6(9):1622–6. Available from: https://www.scopus.com/inward/record.uri?eid=2-s2.0-85055944310&doi=10.3889%2foamjms.2018.346&partnerID=40&md5=813e9051f546562bf44e6c46867fef83

4.	Al Issa A. Epidemiology of trauma injuries in the Districts of Amman, Jordan: Problems and suggestions. J Emerg Med Trauma Acute Care [Internet]. 2008;8(1):35–7. Available from: https://www.scopus.com/inward/record.uri?eid=2-s2.0-56049110862&partnerID=40&md5=8cf940ae545caf4d19096b1895013770

5.	Al-Azzawi IS, Koraitim MM. Lower genitourinary trauma in modern warfare: The experience from civil violence in Iraq. Injury [Internet]. 2014 May [cited 2024 Sep 4];45(5):885–9. Available from: https://linkinghub.elsevier.com/retrieve/pii/S0020138314000187

6.	Appenzeller GN. Injury Patterns in Peacekeeping Missions: The Kosovo Experience. Mil Med [Internet]. 2004;169(3):187–91. Available from: https://www.scopus.com/inward/record.uri?eid=2-s2.0-1542513849&doi=10.7205%2fMILMED.169.3.187&partnerID=40&md5=88afb84ab9ad4ab16c7ddbc1b9c4d6ea

7.	Arora MM, Bhatia JK, Rana KVS. Pattern of fatal injuries in counter terrorist operations: An innovative analysis through embalming services. Med J Armed Forces India [Internet]. 2009;65(2):103–7. Available from: https://www.scopus.com/inward/record.uri?eid=2-s2.0-70349215495&doi=10.1016%2fS0377-1237%2809%2980119-6&partnerID=40&md5=afaae4d678efc883980d2371282a105d

8.	Arul GS, Reynolds J, DiRusso S, Scott A, Bree S, Templeton P, et al. Paediatric admissions to the British military hospital at Camp Bastion, Afghanistan. Ann R Coll Surg Engl [Internet]. 2012;94(1):52–7. Available from: https://www.scopus.com/inward/record.uri?eid=2-s2.0-84855929307&doi=10.1308%2f003588412X13171221499027&partnerID=40&md5=32680c8cfb548a16f8ce36118fc56391

9.	Barzilai L, Harats M, Wiser I, Weissman O, Domniz N, Glassberg E, et al. Characteristics of improvised explosive device trauma casualties in the Gaza strip and other combat regions: The Israeli experience. Wounds [Internet]. 2015;27(8):209–14. Available from: https://www.scopus.com/inward/record.uri?eid=2-s2.0-84939816019&partnerID=40&md5=644bd06fa370358d081eea59e91aa9c6

10.	Belmont PJ, McCriskin BJ, Sieg RN, Burks R, Schoenfeld AJ. Combat wounds in Iraq and Afghanistan from 2005 to 2009. J Trauma Acute Care Surg [Internet]. 2012;73(1):3–12. Available from: https://www.scopus.com/inward/record.uri?eid=2-s2.0-84864186066&doi=10.1097%2fTA.0b013e318250bfb4&partnerID=40&md5=1c1096709a25291bdd1e1a9564e16505

11.	Bhandarwar AH, Bakhshi GD, Tayade MB, Chavan GS, Shenoy SS, Nair AS. Mortality pattern of the 26/11 Mumbai terror attacks. J Trauma Acute Care Surg [Internet]. 2012;72(5):1329–34. Available from: https://www.scopus.com/inward/record.uri?eid=2-s2.0-84864543120&doi=10.1097%2fTA.0b013e31824da04f&partnerID=40&md5=fbfc002a7c46e1358308aac70e588950

12.	Boffard KD, MacFarlane C. Urban bomb blast injuries: patterns of injury and treatment. Surg Annu [Internet]. 1993;25 Pt 1:29–47. Available from: https://www.scopus.com/inward/record.uri?eid=2-s2.0-18244417970&partnerID=40&md5=5e22d9856cb28fc612160cc320232cf0

13.	Born CT, Briggs SM, Ciraulo DL, Frykberg ER, Hammond JS, Hirshberg A, et al. Disasters and mass casualties: II. Explosive, biologic, chemical, and nuclear agents. J Am Acad Orthop Surg [Internet]. 2007;15(8):461–73. Available from: https://www.scopus.com/inward/record.uri?eid=2-s2.0-34547952273&doi=10.5435%2f00124635-200708000-00003&partnerID=40&md5=8d24f507cbea61c1fde2147d6a8e4b46

14.	Boutonnet M, Pasquier P, Jault P, Pierret C, Tourtier JP. Specific patterns around the mangled extremity severity score in blast-related limb injuries. J Trauma - Inj Infect Crit Care [Internet]. 2011;71(3):785. Available from: https://www.scopus.com/inward/record.uri?eid=2-s2.0-80052728427&doi=10.1097%2fTA.0b013e31822ad95f&partnerID=40&md5=9cefa1cb726893db1797201d3b656bdd

15.	Bowley DM, Gillingham S, Mercer S, Schrager JJ, West A. Pneumoperitoneum without visceral trauma: An under-recognised phenomenon after blast injury? J R Army Med Corps [Internet]. 2013;159(4):312–3. Available from: https://www.scopus.com/inward/record.uri?eid=2-s2.0-84888268708&doi=10.1136%2fjramc-2013-000066&partnerID=40&md5=091bfaeb85d7af1a7034e1746cd52ad7

16.	Breeze J, Allanson-Bailey LS, Hepper AE, Midwinter MJ. Demonstrating the effectiveness of body armour: A pilot prospective computerised surface wound mapping trial performed at the role 3 hospital in Afghanistan. J R Army Med Corps [Internet]. 2015;161(1):36–41. Available from: https://www.scopus.com/inward/record.uri?eid=2-s2.0-84988583491&doi=10.1136%2fjramc-2014-000249&partnerID=40&md5=11abdb8fb4b3602dcffb0063f5c14f82

17.	Carley SD, Mackway-Jones K. The casualty profile from the Manchester bombing 1996: a proposal for the construction and dissemination of casualty profiles from major incidents. Emerg Med J [Internet]. 1997 Mar 1 [cited 2025 Apr 4];14(2):76–80. Available from: https://emj.bmj.com/lookup/doi/10.1136/emj.14.2.76

18.	Caro D, Irving M. THE OLD BAILEY BOMB EXPLOSION. The Lancet [Internet]. 1973 Jun [cited 2025 Apr 4];301(7817):1433–5. Available from: https://linkinghub.elsevier.com/retrieve/pii/S0140673673917534

19.	Centers for Disease Control. Blast injuries: Fact sheets for professionals. ACEP Clin Pract Manag Bombings Inj Patterns Care. 2015;

20.	Chaiphrom N, Kanchanaroek K, Khumwattana M. Characteristics and distribution of blast and firearm injuries in Thai military personnel during conflict in southern Thailand. J Med Assoc Thail Chotmaihet Thangphaet [Internet]. 2009;92 Suppl 1:S47-52. Available from: https://www.scopus.com/inward/record.uri?eid=2-s2.0-79952273646&partnerID=40&md5=8840c5ce68e462bc710a9aa21ebb0626

21.	Chan RK, Siller-Jackson A, Verrett AJ, Wu J, Hale RG. Ten years of war: A characterization of craniomaxillofacial injuries incurred during operations Enduring Freedom and Iraqi Freedom. J Trauma Acute Care Surg [Internet]. 2012;73(6 SUPPL. 5):S453–8. Available from: https://www.scopus.com/inward/record.uri?eid=2-s2.0-84871215832&doi=10.1097%2fTA.0b013e3182754868&partnerID=40&md5=62d6b94734b634423c46791a149e82cd

22.	Chianakwana GU, Mbonu OO, Egwuonwu AO, Azike J, Eleweke N, Ekwunife C, et al. MISSILE AND BLAST INJURIES IN NIGERIA - THE SOUTHEAST EXPERIENCE. J West Afr Coll Surg. 2017;7(4):18–33.

23.	Christensen MC, Banner C, Lefering R, Vallejo-Torres L, Morris S. Quality of Life After Severe Trauma: Results From the Global Trauma Trial With Recombinant Factor VII. J Trauma Inj Infect Crit Care [Internet]. 2011 Jun [cited 2024 Aug 24];70(6):1524–31. Available from: https://journals.lww.com/00005373-201106000-00033

24.	Cooper GJ, Maynard RL, Cross NL, Hill JF. Casualties from terrorist bombings. J Trauma - Inj Infect Crit Care [Internet]. 1983;23(11):955–67. Available from: https://www.scopus.com/inward/record.uri?eid=2-s2.0-0021047668&doi=10.1097%2f00005373-198311000-00001&partnerID=40&md5=c9893f816064396230257b5c85614ddf

25.	Covey DC, Born CT. Blast injuries: mechanics and wounding patterns. J Surg Orthop Adv [Internet]. 2010;19(1):8–12. Available from: https://www.scopus.com/inward/record.uri?eid=2-s2.0-77951882120&partnerID=40&md5=9c960a0dbedccbeb1eae60b2d7deff40

26.	Daw MA, El-Bouzedi AH, Dau AA. Trends and patterns of deaths, injuries and intentional disabilities within the Libyan armed conflict: 2012-2017. PLoS ONE [Internet]. 2019;14(5). Available from: https://www.scopus.com/inward/record.uri?eid=2-s2.0-85065791049&doi=10.1371%2fjournal.pone.0216061&partnerID=40&md5=880cacd1e7fa9cd06408538b31ea83e0

27.	Delannoy Y, Delabarde T, Plu I, Legrand L, Taccoen M, Tracqui A, et al. Terrorist explosive belt attacks: specific patterns of bone traumas. Int J Legal Med [Internet]. 2019;133(2):565–9. Available from: https://www.scopus.com/inward/record.uri?eid=2-s2.0-85057583973&doi=10.1007%2fs00414-018-1977-3&partnerID=40&md5=d06be0edb775c164dca5a9c94d58b75a

28.	Delannoy Y, Plu I, Sec I, Delabarde T, Taccoen M, Tracqui A, et al. Terrorist attacks: cutaneous patterns of gunshot and secondary blast injuries. Forensic Sci Res [Internet]. 2020;5(3):208–13. Available from: https://www.scopus.com/inward/record.uri?eid=2-s2.0-85087616542&doi=10.1080%2f20961790.2020.1771859&partnerID=40&md5=a68c25a5e94892bfe736bff34afa9232

29.	Dootz F, von Stackelberg OM, Abaya J, Jacobi C, Mohs C, Craemer EM, et al. Better be prepared: the spectrum of neuropsychiatric impairment among Libyan war victims transferred to Germany for trauma rehabilitation. Neurol Res Pract [Internet]. 2021;3(1). Available from: https://www.scopus.com/inward/record.uri?eid=2-s2.0-85132670198&doi=10.1186%2fs42466-021-00134-z&partnerID=40&md5=d11010649ed54fd6658cb3e9d305274b

30.	Doucet JJ, Galarneau MR, Potenza BM, Bansal V, Lee JG, Schwartz AK, et al. Combat Versus Civilian Open Tibia Fractures: The Effect of Blast Mechanism on Limb Salvage. J Trauma Inj Infect Crit Care [Internet]. 2011 May [cited 2025 Apr 4];70(5):1241–7. Available from: https://journals.lww.com/00005373-201105000-00032

31.	Dunn JC, Eckhoff MD, Nicholson TC, Campbell W, Kenney K, Smith J, et al. Combat-Sustained Peripheral Nerve Injuries in the United States Military. J Hand Surg [Internet]. 2021;46(2):148.e1-148.e8. Available from: https://www.scopus.com/inward/record.uri?eid=2-s2.0-85092010338&doi=10.1016%2fj.jhsa.2020.08.004&partnerID=40&md5=4f8e0f871862b13cd8d7a7e6f943cb08

32.	Dunn JC, Kusnezov N, Schoenfeld AJ, Orr JD, Cook PJ, Belmont PJ. Vascular Injuries in Combat-Specific Soldiers during Operation Iraqi Freedom and Operation Enduring Freedom. Ann Vasc Surg [Internet]. 2016;35:30–7. Available from: https://www.scopus.com/inward/record.uri?eid=2-s2.0-84992437549&doi=10.1016%2fj.avsg.2016.01.040&partnerID=40&md5=fb47ce49a4befaaf446c6456837d03a7

33.	Dussault MC, Smith M, Hanson I. Evaluation of trauma patterns in blast injuries using multiple correspondence analysis. Forensic Sci Int [Internet]. 2016;267:66–72. Available from: https://www.scopus.com/inward/record.uri?eid=2-s2.0-84983591306&doi=10.1016%2fj.forsciint.2016.08.004&partnerID=40&md5=6abb81310c27c0181baaadc1fb7a3145

34.	Dussault MC, Smith M, Osselton D. Blast injury and the human skeleton: An important emerging aspect of conflict-related trauma. J Forensic Sci [Internet]. 2014;59(3):606–12. Available from: https://www.scopus.com/inward/record.uri?eid=2-s2.0-84899121743&doi=10.1111%2f1556-4029.12361&partnerID=40&md5=9ac09e018628afc33cd95980db29aeb4

35.	Eardley WGP, Bonner TJ, Gibb IE, Clasper JC. Spinal fractures in current military deployments. J R Army Med Corps [Internet]. 2012;158(2):101–5. Available from: https://www.scopus.com/inward/record.uri?eid=2-s2.0-84866909757&doi=10.1136%2fjramc-158-02-06&partnerID=40&md5=0c4b02431dc86a57d4516ca6db738b43

36.	Eastridge BJ. Injuries to the Abdomen from Explosion. Curr Trauma Rep [Internet]. 2017;3(1):69–74. Available from: https://www.scopus.com/inward/record.uri?eid=2-s2.0-85081712652&doi=10.1007%2fs40719-017-0079-x&partnerID=40&md5=19e0c2fd076e67895317b664e0a41e1d

37.	Edirisinghe A, Samarasekera A. Injury, Fatal and Nonfatal: Explosive Injuries. In: Encyclopedia of Forensic and Legal Medicine: Second Edition [Internet]. 2015. p. 182–202. Available from: https://www.scopus.com/inward/record.uri?eid=2-s2.0-85043252405&doi=10.1016%2fB978-0-12-800034-2.00221-4&partnerID=40&md5=db96c401e894a46329fcdaf30769c9b4

38.	Farhat T, Nahouli H, Hajjar M, Abdul-Sater Z, Kobeissi E, Menassa M, et al. Characteristics of injuries during the 2006 Lebanon conflict: a three-center retrospective study of survivors, 16 years after the conflict. Front Public Health. 2024;12:1382514.

39.	Feenstra O. [Various forms of explosion injuries]. Z Rechtsmed. 1986;96(1):27–30.

40.	Fogel I, Balziano S, Tunik M, Prat D, Barzilay R, Greenstein N. Efficient evacuation - enhanced survival: Insights from Gaza conflict trauma care. J Trauma Acute Care Surg. 2025;

41.	Fox CJ, Gillespie DL, Weber MA, Cox MW, Hawksworth JS, Cryer CM, et al. Delayed evaluation of combat-related penetrating neck trauma. J Vasc Surg [Internet]. 2006;44(1):86–93. Available from: https://www.scopus.com/inward/record.uri?eid=2-s2.0-33745676516&doi=10.1016%2fj.jvs.2006.02.058&partnerID=40&md5=eeaa9d527105466432abc57ed30f39d1

42.	Gataa IS, Muassa QH. Patterns of maxillofacial injuries caused by terrorist attacks in Iraq: Retrospective study. Int J Oral Maxillofac Surg [Internet]. 2011;40(1):65–70. Available from: https://www.scopus.com/inward/record.uri?eid=2-s2.0-79251650366&doi=10.1016%2fj.ijom.2010.07.005&partnerID=40&md5=6a8811d82f46dca12f0d9fb163d7d6fb

43.	Giessler GA, Leopold A, Germann G, Heitmann C. Blast injuries of the hands. Patterns of trauma and plastic surgical treatment. Unfallchirurg [Internet]. 2006;109(11):956–63. Available from: https://www.scopus.com/inward/record.uri?eid=2-s2.0-33845448290&doi=10.1007%2fs00113-006-1139-9&partnerID=40&md5=4a0f1825e14dc27f55f49e495ae43a67

44.	Goksel T. Improvised explosive devices and the Oral and Maxillofacial Surgeon. Oral Maxillofac Surg Clin N Am [Internet]. 2005;17(3 SPEC. ISS.):281–7. Available from: https://www.scopus.com/inward/record.uri?eid=2-s2.0-22644433239&doi=10.1016%2fj.coms.2005.05.002&partnerID=40&md5=28baebf145e05d0c6af5771584d48fac

45.	Gordon W, Talbot M, Fleming M, Shero J, Potter B, Stockinger ZT. High bilateral amputations and dismounted complex blast injury (DCBI). Mil Med [Internet]. 2018;183:118–22. Available from: https://www.scopus.com/inward/record.uri?eid=2-s2.0-85055797555&doi=10.1093%2fmilmed%2fusy082&partnerID=40&md5=845e9ba7a5bdf5cabfcad8e2b62cb586

46.	Gurney JM. Case Study from Afghanistan: Dismounted Complex Blast Injury. In: Operational and Medical Management of Explosive and Blast Incidents [Internet]. 2020. p. 559–66. Available from: https://www.scopus.com/inward/record.uri?eid=2-s2.0-85151207999&doi=10.1007%2f978-3-030-40655-4_41&partnerID=40&md5=0a593a305d8b699fafe462abccb39cdc

47.	Hahn P, Brederlau J, Krimmer H, Lanz U. Explosion injuries of the hand: Spatial relationship and injury pattern. J Hand Surg Eur Vol [Internet]. 1996;21(6):785–7. Available from: https://www.scopus.com/inward/record.uri?eid=2-s2.0-0029773287&doi=10.1016%2fS0266-7681%2896%2980191-4&partnerID=40&md5=bc749370686bf83c3cc2cae519998f1c

48.	Hazani R, Buntic RF, Brooks D. Patterns in blast injuries to the hand. Hand [Internet]. 2009;4(1):44–9. Available from: https://www.scopus.com/inward/record.uri?eid=2-s2.0-62749110719&doi=10.1007%2fs11552-008-9125-z&partnerID=40&md5=4413a44c3c3707d7f6bb4b248408de5b

49.	Helling ER. Otologic blast injuries due to the Kenya Embassy bombing. Mil Med [Internet]. 2004;169(11):872–6. Available from: https://www.scopus.com/inward/record.uri?eid=2-s2.0-16644387780&doi=10.7205%2fMILMED.169.11.872&partnerID=40&md5=b8be59dbfd6eb9625e9d60036890f1ef

50.	Helou M, Weinstein ES, Kalaji J, Chaaban T, Yammine K. Pager Explosion in Beirut: An Unprecedented Event. Disaster Med Public Health Prep. 2024;18:e215.

51.	Hesling JD, Paulson MW, McKay JT, Bebarta VS, Flarity K, Keenan S, et al. Characterizing pediatric supermassive transfusion and the contributing injury patterns in the combat environment. Am J Emerg Med [Internet]. 2022;51:139–43. Available from: https://www.scopus.com/inward/record.uri?eid=2-s2.0-85118489118&doi=10.1016%2fj.ajem.2021.10.032&partnerID=40&md5=621a4993024d64f745010552c942620b

52.	Hodge N. US focuses study on blast injuries. Janes Def Wkly [Internet]. 2008;(MAY). Available from: https://www.scopus.com/inward/record.uri?eid=2-s2.0-80053171199&partnerID=40&md5=7c23eedbe6c82c98cafbf2e472bab7b4

53.	Hoencamp R, Idenburg FJ, Hamming JF, Tan ECTH. Incidence and epidemiology of casualties treated at the dutch role 2 enhanced medical treatment facility at multi national base Tarin Kowt, Afghanistan in the period 2006-2010. World J Surg [Internet]. 2014;38(7):1713–8. Available from: https://www.scopus.com/inward/record.uri?eid=2-s2.0-84905085072&doi=10.1007%2fs00268-014-2462-x&partnerID=40&md5=926264a2e15a60e7c3cc795075e14ba2

54.	Hoencamp R, Huizinga EP, van Dongen TT, Idenburg FJ, Ramasamy A, Leenen LP, et al. Impact of explosive devices in modern armed conflicts: in-depth analysis of Dutch battle casualties in southern Afghanistan. World J Surg. 2014;38(10):2551–7.

55.	Hoencamp R, Huizinga EP, Van Dongen TTCF, Idenburg FJ, Ramasamy A, Leenen LPH, et al. Impact of explosive devices in modern armed conflicts: In-depth analysis of dutch battle casualties in southern afghanistan. World J Surg [Internet]. 2015;38(10):2551–7. Available from: https://www.scopus.com/inward/record.uri?eid=2-s2.0-84939883695&doi=10.1007%2fs00268-014-2645-5&partnerID=40&md5=5d7e5df6fff2ac3e623ffc94c300a7d0

56.	Hudak SJ, Morey AF, Rozanski TA, Fox Jr. CW. Battlefield urogenital injuries: Changing patterns during the past century. Urology [Internet]. 2005;65(6):1041–6. Available from: https://www.scopus.com/inward/record.uri?eid=2-s2.0-20444449762&doi=10.1016%2fj.urology.2004.11.031&partnerID=40&md5=6677c662e8dfe415917cb56a756c428a

57.	Hull JB. Blast: injury patterns and their recording. J Audiov Media Med [Internet]. 1992 Jan [cited 2024 Apr 5];15(3):121–7. Available from: http://www.tandfonline.com/doi/full/10.3109/17453059209018366

58.	Ianov IK, Gofman VR, Glaznikov LA, Maksimova TG. Clinical and statistical patterns of the hearing system lesions caused by explosive mine trauma. Voen Med Zh [Internet]. 2001;322(9):32–7, 96. Available from: https://www.scopus.com/inward/record.uri?eid=2-s2.0-18344410686&partnerID=40&md5=7c27bce6fdddafba0bb8992c3268100e

59.	Islinger RB, Kuklo TR, McHale KA. A review of orthopedic injuries in three recent U.S. military conflicts. Mil Med [Internet]. 2000;165(6):463–5. Available from: https://www.scopus.com/inward/record.uri?eid=2-s2.0-0034095559&doi=10.1093%2fmilmed%2f165.6.463&partnerID=40&md5=ae3bb44603ff88fd915fb6203d2a8189

60.	Jacobs N, Taylor DM, Parker PJ. Changes in surgical workload at the JF Med Gp Role 3 Hospital, Camp Bastion, Afghanistan, November 2008-November 2010. Injury [Internet]. 2012;43(7):1037–40. Available from: https://www.scopus.com/inward/record.uri?eid=2-s2.0-84862889736&doi=10.1016%2fj.injury.2011.12.017&partnerID=40&md5=a293c63ac0098f03de64bdc7b7ea66fe

61.	Jahangiri K, Ghodsi H, Khodadadizadeh A, Yousef Nezhad S. Pattern and nature of Neyshabur train explosion blast injuries. World J Emerg Surg [Internet]. 2018;13(1). Available from: https://www.scopus.com/inward/record.uri?eid=2-s2.0-85040720365&doi=10.1186%2fs13017-018-0164-7&partnerID=40&md5=a083a3a99324f80a219f347316448468

62.	Karmy-Jones R, Kissinger D, Golocovsky M, Jordan M, Champion HR. Bomb-related injuries. Mil Med. 1994 Jul;159(7):536–9.

63.	Khosravi MH, Akhavan A. Facial injuries in Iranian veterans during the Iraq-Iran war (1980-88): Differences from recent conflicts. Br J Oral Maxillofac Surg [Internet]. 2015;53(10):949–52. Available from: https://www.scopus.com/inward/record.uri?eid=2-s2.0-84960412296&doi=10.1016%2fj.bjoms.2015.06.021&partnerID=40&md5=b1647f1cf07b2aa6befad5ad54c02e72

64.	Khurana P, Dalal JS. Bomb blast injuries. J Punjab Acad Forensic Med Toxicol [Internet]. 2011;11(1):37–9. Available from: https://www.scopus.com/inward/record.uri?eid=2-s2.0-80053167741&partnerID=40&md5=e704e0825fdfc7ba873d303351547917

65.	Khurana P, Dalal JS. Bomb blast injuries-an update for forensic pathologist and clinicians. Indian J Forensic Med Toxicol [Internet]. 2012;6(1):64–7. Available from: https://www.scopus.com/inward/record.uri?eid=2-s2.0-84861936192&partnerID=40&md5=e4b49ed48c5db0cd43ac9b559c011459

66.	Kittle CP, Verrett AJ, Wu J, Mellus DE, Hale RG, Chan RK. Characterization of midface fractures incurred in recent wars. J Craniofac Surg [Internet]. 2012;23(6):1587–91. Available from: https://www.scopus.com/inward/record.uri?eid=2-s2.0-84873292010&doi=10.1097%2fSCS.0b013e318256514a&partnerID=40&md5=b351a7058d876a9d437a63a20b5b6d7d

67.	Kluger Y, Peleg K, Daniel-Aharonson L, Mayo A. The special injury pattern in terrorist bombings. J Am Coll Surg [Internet]. 2004;199(6):875–9. Available from: https://www.scopus.com/inward/record.uri?eid=2-s2.0-8844250845&doi=10.1016%2fj.jamcollsurg.2004.09.003&partnerID=40&md5=06d785fbfc7aa11fd5f418acda2585d4

68.	Kluger Y, Soffer D, Sorkine P, Aladgem D. Blast injury: detonation and injury pattern. Harefuah [Internet]. 1997;132(8):591–3. Available from: https://www.scopus.com/inward/record.uri?eid=2-s2.0-0031569534&partnerID=40&md5=fa5ac14cc3f1323c5763e2c14edce941

69.	Kosashvili Y, Loebenberg MI, Lin G, Peleg K, Zvi F, Kluger Y, et al. Medical consequences of suicide bombing mass casualty incidents: The impact of explosion setting on injury patterns. Injury [Internet]. 2009;40(7):698–702. Available from: https://www.scopus.com/inward/record.uri?eid=2-s2.0-67349147511&doi=10.1016%2fj.injury.2008.06.037&partnerID=40&md5=8d1d713a7e69473cf45c6c75360e77d8

70.	Krishtafor DA, Klygunenko OM, Krishtafor AA. Comparative characteristics of civilian and military multiple trauma in a level III hospital. Emerg Med Ukr [Internet]. 2019;3(98):127–33. Available from: https://www.scopus.com/inward/record.uri?eid=2-s2.0-85099826824&doi=10.22141%2f2224-0586.3.98.2019.165490&partnerID=40&md5=e757e6b5c72f0a739e385a582a4aaf19

71.	Kuckelman J, Cuadrado D, Martin M. Thoracic Trauma: a Combat and Military Perspective. Curr Trauma Rep [Internet]. 2018;4(1):77–87. Available from: https://www.scopus.com/inward/record.uri?eid=2-s2.0-85079442405&doi=10.1007%2fs40719-018-0112-8&partnerID=40&md5=608b1280f3671172bf05a402e7b4b7ab

72.	Lafta R, Al-Shatari S, Cherewick M, Galway L, Mock C, Hagopian A, et al. Injuries, death, and disability associated with 11 years of conflict in Baghdad, Iraq: A randomized household cluster survey. PLoS ONE [Internet]. 2015;10(8). Available from: https://www.scopus.com/inward/record.uri?eid=2-s2.0-84942134282&doi=10.1371%2fjournal.pone.0131834&partnerID=40&md5=dc746d844c98e710c48a3dec8f8786fd

73.	Langworthy MJ. Pattern of injuries after terrorist blast attack. Folia Traumatol Belg. 2002;82:77–8.

74.	Lawry LL, Mani V, Hamm TE, Janvrin M, Juman L, Korona-Bailey J, et al. Qualitative assessment of combat-related injury patterns and injury prevention in Ukraine since the Russian invasion. BMJ Mil Health. 2025;

75.	Lechner R, Achatz G, Hauer T, Palm HG, Lieber A, Willy C. Patterns and causes of injuries in a contemporary combat environment. Unfallchirurg [Internet]. 2010;113(2):106–13. Available from: https://www.scopus.com/inward/record.uri?eid=2-s2.0-77149166007&doi=10.1007%2fs00113-009-1732-9&partnerID=40&md5=f120bb5b298699f7125f3cedde355583

76.	Machaku D, Chambo M, Nkoronko M, Shadrack M, Sadiq A, Msuya D. Ocular, scrotal and abdominal trauma in a secondary blast injury. J Surg Case Rep. 2023;2023(3):rjad126.

77.	Malie PM, Sonnier Jr. MQ, Berkowitz MR. Understanding blast-induced traumatic brain injury (bTBI) through mechanisms and patterns of injury. AAO J [Internet]. 2012;22(2):10–6. Available from: https://www.scopus.com/inward/record.uri?eid=2-s2.0-84865251533&partnerID=40&md5=7b10a54e3181d360af0470f04cabd0c7

78.	Mamczak CN, Malish D, Boonstra O. A rare case of paediatric pelvic ring injury with lower urinary tract obstruction secondary to a combat blast mechanism. Injury [Internet]. 2013;44(7):983–6. Available from: https://www.scopus.com/inward/record.uri?eid=2-s2.0-84878781423&doi=10.1016%2fj.injury.2013.04.013&partnerID=40&md5=9e6a02eab7f4d7489e9f560963003720

79.	Maqsood R, Rasikh A, Abbasi T, Shukr I. PATTERN OF INJURIES SEEN IN MASS CASUALTIES IN TERRORIST ATTACKS IN BALUCHISTAN, PAKISTAN--A THREE YEARS EXPERIENCE. J Ayub Med Coll Abbottabad JAMC [Internet]. 2015;27(4):858–60. Available from: https://www.scopus.com/inward/record.uri?eid=2-s2.0-84979855833&partnerID=40&md5=1ac6c7251b7a2c77bfd860574e57e67a

80.	Master PB, Sekhar VC, Rangaiah YKC. Bomb blast: pattern and nature of injuries. J Evid Based Med Healthc. 2015;2(2):165–71.

81.	Mayo A, Kluger Y. Terrorist bombing. World J Emerg Surg [Internet]. 2006;1(1). Available from: https://www.scopus.com/inward/record.uri?eid=2-s2.0-34248151587&doi=10.1186%2f1749-7922-1-33&partnerID=40&md5=33eeda309ccf85b9f4c26210f02eb350

82.	McLean C, Hull J. Missile and explosive wounds. Surgery [Internet]. 2006;24(6):194–7. Available from: https://www.scopus.com/inward/record.uri?eid=2-s2.0-33845266137&doi=10.1383%2fsurg.2006.24.6.194&partnerID=40&md5=4b7e9f7ae4254a97a02c13fad777074a

83.	McMenemy L, Mondini V, Roberts DC, Kedgley A, Clasper JC, Stapley SA. Pattern of upper limb amputation associated with lower limb amputation: The UK military experience from Iraq and Afghanistan. BMJ Mil Health [Internet]. 2021; Available from: https://www.scopus.com/inward/record.uri?eid=2-s2.0-85105339537&doi=10.1136%2fbmjmilitary-2021-001783&partnerID=40&md5=1f6ea0a1c28b3a1a7552a0f219f7ea98

84.	Mohammad NK, Al-Mahdi AH, Mohammed OM. Ophthalmic injuries related to maxillofacial trauma due to urban warfare. J Craniofac Surg [Internet]. 2018;29(7):1804–8. Available from: https://www.scopus.com/inward/record.uri?eid=2-s2.0-85054430522&doi=10.1097%2fSCS.0000000000004963&partnerID=40&md5=bf51933f5392c421a00d57fba0341529

85.	Molaie SH, Mahmoudi S, Goodarzi H, Danial Z, Farajzadeh MA, Pakravesh M, et al. Assessment of injuries following terrorist attacks: A narrative review. Trauma Mon [Internet]. 2020;25(1):8–13. Available from: https://www.scopus.com/inward/record.uri?eid=2-s2.0-85090516372&doi=10.30491%2fTM.2020.105838&partnerID=40&md5=b85f64488ed5a5fb162044f632175f9c

86.	Morley B, Leslie GD. Terrorist bombings: Motives, methods and patterns of injuries. Australas Emerg Nurs J [Internet]. 2007;10(1):5–12. Available from: https://www.scopus.com/inward/record.uri?eid=2-s2.0-33847169348&doi=10.1016%2fj.aenj.2006.07.001&partnerID=40&md5=88b7c0511e60ab1c341b1568611ab1a0

87.	Mühlmeier G, Tisch M. [Blast and explosion traumas-effects on the middle and inner ear based on the example of Bundeswehr foreign missions]. HNO. 2023;71(1):48–56.

88.	Nor MA, Sonmez RE, Hassan MY, Ozsoy MS, Alimoglu O. Patterns of injury and death in mass casualty incidents from 2013 to 2018 in Mogadishu, Somalia Mass casualty incidents in Mogadishu, Somalia. Bangladesh J Med Sci [Internet]. 2023;22(2):323–8. Available from: https://www.scopus.com/inward/record.uri?eid=2-s2.0-85158084688&doi=10.3329%2fbjms.v22i2.64989&partnerID=40&md5=a868c71c2e11bf82abd37ff932250dba

89.	Nunziato C, Williams J, Williams R. Synthetic Bone Graft Substitute for Treatment of Unicameral Bone Cysts. J Pediatr Orthop. 2021;41(1):e60–6.

90.	Ostfeld I, Kachel E, Shafir O, Gal I, Cohen Y, Kugel C, et al. The October 7, 2023, Attack on Israel Indicates a Change in Trauma Patterns of Terror Victims, due to the Use of Thermobaric Weapons. Mil Med. 2024;

91.	Parker W, Despain RW, Bailey J, Elster E, Rodriguez CJ, Bradley M. Military experience in the management of pelvic fractures from OIF/OEF. BMJ Mil Health. 2023;169(2):108–11.

92.	Paul AO, Kay MV, Seitz S, Kanz KG. Injury patterns in bombings. MMW-Fortschritte Med [Internet]. 2008;150(9):42–3. Available from: https://www.scopus.com/inward/record.uri?eid=2-s2.0-40649100089&doi=10.1007%2fbf03370170&partnerID=40&md5=2c73c946978df9cc6081dcb176b84997

93.	Paydar S, Sharifian M, Parvaz S, Abbasi H, Moradian M, Roozbeh J, et al. Explosive attack: Lessons learned in Seyed Al Shohada mosque attack, April 2008, Shiraz, Iran. J Emerg Trauma Shock [Internet]. 2012 [cited 2025 Apr 4];5(4):296. Available from: https://journals.lww.com/10.4103/0974-2700.102363

94.	Peleg K, Aharonson-Daniel L, Michael M, Shapira SC. Patterns of injury in hospitalized terrorist victims. Am J Emerg Med. 2003;21(4):258–62.

95.	Peramaki ER. Pictorial review of radiographic patterns of injury in modern warfare: Imaging the conflict in Afghanistan. Can Assoc Radiol J [Internet]. 2011;62(2):90–106. Available from: https://www.scopus.com/inward/record.uri?eid=2-s2.0-79955020111&doi=10.1016%2fj.carj.2010.03.005&partnerID=40&md5=23558167d5ce0ec6b90f3c01a3d6da61

96.	Pillgram-Larsen J, Mellesmo S, Peck R. Injuries from mines. Tidsskr Nor Laegeforen [Internet]. 1992;112(17):2183–7. Available from: https://www.scopus.com/inward/record.uri?eid=2-s2.0-0027129341&partnerID=40&md5=98529c88624d9f7c1295f30ba8164427

97.	Poon H, Morrison JJ, Apodaca AN, Khan MA, Garner JP. The UK military experience of thoracic injury in the wars in Iraq and Afghanistan. Injury [Internet]. 2013 Sep [cited 2024 Apr 5];44(9):1165–70. Available from: https://linkinghub.elsevier.com/retrieve/pii/S0020138313000739

98.	Powers DB, Rodriguez ED. Characteristics of Ballistic and Blast Injuries. In: Facial Trauma Surgery: From Primary Repair to Reconstruction [Internet]. 2019. p. 261–72. Available from: https://www.scopus.com/inward/record.uri?eid=2-s2.0-85124860775&doi=10.1016%2fB978-0-323-49755-8.00028-1&partnerID=40&md5=494164bed228df730e63223e7f2b3c80

99.	Powers DB, Delo RI. Characteristics of ballistic and blast injuries. Atlas Oral Maxillofac Surg Clin North Am. 2013;21(1):15–24.

100.	Pronk D. Prehospital recognition of wounding patterns in blast injuries. J Emerg Med Serv.

101.	Rafaels KA, Gillich PJ, Ehlers RZ, Duvall PS. Lower leg injuries in dismounted military personnel from buried explosives. In 2013. p. 171–82. Available from: https://www.scopus.com/inward/record.uri?eid=2-s2.0-84896614207&partnerID=40&md5=4a111f7840cc731bfb702b5d4f5ec22c

102.	Razaq S, Yasmeen R, Butt AW, Akhtar N, Mansoor SN. The pattern of peripheral nerve injuries among Pakistani soldiers in the war against terror. J Coll Physicians Surg Pak [Internet]. 2015;25(5):363–6. Available from: https://www.scopus.com/inward/record.uri?eid=2-s2.0-84929669243&partnerID=40&md5=8c187210c4468b15d2de3ae528b0679b

103.	Rice D, Heck J. Terrorist bombings: Ballistics, patterns of blast injury and tactical emergency care. Tactical Edge J. 2000;53–5.

104.	Ruwanpura R, Kumara KS, Jayawardane H, de Alwis LB. Suicidal bomb explosions in Sri Lanka. Am J Disaster Med [Internet]. 2008;3(1):47–51. Available from: https://www.scopus.com/inward/record.uri?eid=2-s2.0-46449133675&doi=10.5055%2fajdm.2008.0006&partnerID=40&md5=27653ac4338ea4152b7c7ba3c398f4bc

105.	Saravanapavananthan N. Injuries caused by home-made explosives. Forensic Sci Int [Internet]. 1978;12(2):131–6. Available from: https://www.scopus.com/inward/record.uri?eid=2-s2.0-0018227119&doi=10.1016%2f0379-0738%2878%2990021-X&partnerID=40&md5=fbb2803087a356c12ca1e090d2eeabae

106.	Schmidbauer W, Lenz S, Bubser F, Kerner T. Blast injury. Anasthesiol Intensivmed Notfallmed Schmerzther [Internet]. 2010;45(6):372–7. Available from: https://www.scopus.com/inward/record.uri?eid=2-s2.0-77953668379&doi=10.1055%2fs-0030-1255342&partnerID=40&md5=8afb72e620de42580bbdbf956b1e2936

107.	Schoenfeld AJ, Newcomb RL, Pallis MP, Cleveland AW, Serrano JA, Bader JO, et al. Characterization of spinal injuries sustained by American service members killed in Iraq and Afghanistan: A study of 2,089 instances of spine trauma. J Trauma Acute Care Surg [Internet]. 2013;74(4):1112–8. Available from: https://www.scopus.com/inward/record.uri?eid=2-s2.0-84876162618&doi=10.1097%2fTA.0b013e31828273be&partnerID=40&md5=843fc358e19a4e0cc48c9c80892ba73b

108.	Schwartz D, Glassberg E, Nadler R, Hirschhorn G, Marom OC, Aharonson-Daniel L. Injury patterns of soldiers in the second Lebanon war. J Trauma Acute Care Surg [Internet]. 2014;76(1):160–6. Available from: https://www.scopus.com/inward/record.uri?eid=2-s2.0-84892158856&doi=10.1097%2fTA.0b013e3182a9680e&partnerID=40&md5=5119fb3aabb79a8a7fea7c1f27f2f734

109.	Shankar YU. Injuries due to explosion – A case report. Indian J Forensic Med Toxicol [Internet]. 2016;10(2):94–8. Available from: https://www.scopus.com/inward/record.uri?eid=2-s2.0-84983284940&doi=10.5958%2f0973-9130.2016.00070.0&partnerID=40&md5=86abeda2d64b5a78debde5c798111019

110.	Singh AK, Ditkofsky NG, York JD, Abujudeh HH, Avery LA, Brunner JF, et al. Blast injuries: From improvised explosive device blasts to the Boston Marathon bombing. Radiographics [Internet]. 2016;36(1):295–307. Available from: https://www.scopus.com/inward/record.uri?eid=2-s2.0-84955444182&doi=10.1148%2frg.2016150114&partnerID=40&md5=cab555984a8dc0fc1a2d3c76180108bc

111.	Sirko A, Berlin C, Tsang S, Naik BI, Armonda R. Wartime penetrating traumatic brain injury of the anterior skull base involving the paranasal sinuses: a single-center, first-year experience from Dnipro, Ukraine. J Neurosurg. 2025;142(3):829–38.

112.	Sorkin P, Nimrod A, Biderman P. The quinary (Vth) injury pattern of blast. J Trauma. 2004;56.

113.	Stemper BD, Yoganandan N, Paskoff GR, Fijalkowski RJ, Storvik SG, Baisden JL, et al. Thoracolumbar spine trauma in military environments. Minerva Ortop E Traumatol [Internet]. 2011;62(5):397–412. Available from: https://www.scopus.com/inward/record.uri?eid=2-s2.0-82955217181&partnerID=40&md5=1f5235d94f5a2d3463aa1c872de92121

114.	Stevenson C. Evolving mechanisms and patterns of blast injury and the challenges for military first responders. J Mil Veteran Health. 2009;17:17–21.

115.	Tekade P, Parakh P, Ughade J, Chahankar SJ, Tekade CR, Kardile PB. Study of Blast Injuries in Tribal Region of Bastar: A Five Year Study. Medico-Leg Update [Internet]. 2017 [cited 2024 Sep 4];17(2):101. Available from: http://www.indianjournals.com/ijor.aspx?target=ijor:mlu&volume=17&issue=2&article=023

116.	Tin D, Galehan J, Markovic V, Ciottone GR. Suicide bombing terrorism. Prehospital Disaster Med [Internet]. 2021;36(6):664–8. Available from: https://www.scopus.com/inward/record.uri?eid=2-s2.0-85118309579&doi=10.1017%2fS1049023X21001151&partnerID=40&md5=12ca5306e6c6279c21d4ed8026287961

117.	Tintle SM, Keeling JJ, Shawen SB. Combat foot and ankle trauma. J Surg Orthop Adv [Internet]. 2010;19(1):70–6. Available from: https://www.scopus.com/inward/record.uri?eid=2-s2.0-77951894973&partnerID=40&md5=d2515c8f44b4450f4532feb87a3eee6f

118.	Tovar MA, Bell RS, Neal CJ. Epidemiology of Blast Neurotrauma: A Meta-analysis of Blast Injury Patterns in the Military and Civilian Populations. World Neurosurg [Internet]. 2021;146:308-314.e3. Available from: https://www.scopus.com/inward/record.uri?eid=2-s2.0-85097910952&doi=10.1016%2fj.wneu.2020.11.093&partnerID=40&md5=b2c0b2f26730ad48de0926f1fb5e2717

119.	Turégano-Fuentes F, Pérez-Diaz D, Sanz-Sánchez M, Alfici R, Ashkenazi I. Abdominal blast injuries: Different patterns, severity, management, and prognosis according to the main mechanism of injury. Eur J Trauma Emerg Surg [Internet]. 2014;40(4):451–60. Available from: https://www.scopus.com/inward/record.uri?eid=2-s2.0-84906307664&doi=10.1007%2fs00068-014-0397-4&partnerID=40&md5=af3f890679b7f378e6a2c169953b522b

120.	Vander Linden L, Bronselaer K. Blast injury. Tijdschr Voor Geneeskd [Internet]. 2017;73(23):1445–54. Available from: https://www.scopus.com/inward/record.uri?eid=2-s2.0-85037606905&doi=10.2143%2fTVG.73.23.2002474&partnerID=40&md5=358c5786db63fcd46f7e370921b0e3bf

121.	Webster C, Masouros S, Gibb I, Clasper J. Fracture patterns in pelvic blast injury: A retrospective analysis and implications for future preventative strategies. Orthop Proc. 2015;97.

122.	Wightman JM, Gladish SL. Explosions and blast injuries. Ann Emerg Med [Internet]. 2001;37(6):664–78. Available from: https://www.scopus.com/inward/record.uri?eid=2-s2.0-0034998821&doi=10.1067%2fmem.2001.114906&partnerID=40&md5=a45da281cf6ed35f1b91af74eb38b30a

123.	Willits NA, Hefner JT, Tersigni-Tarrant MA. Case studies in skeletal blast trauma. In: Skeletal Trauma Analysis: Case Studies in Context [Internet]. 2015. p. 177–88. Available from: https://www.scopus.com/inward/record.uri?eid=2-s2.0-85016374725&doi=10.1002%2f9781118384213.ch14&partnerID=40&md5=b1f796a9ef19a0e458623651f86e8560

124.	Wold AM, Justin GA, Hobbs SD, Baker KM, Brady DP, Aden JK, et al. POSTERIOR SEGMENT INJURIES IN OPERATION IRAQI FREEDOM AND OPERATION ENDURING FREEDOM: 2001 to 2011. Retina. 2021;41(12):2564–70.

125.	Yunusa B, Aji S, Mashi SA, Abdullahi M, Abubakar MK, Sheshe AA, et al. Experiences and challenges of managing mass casualty during industrial action in Aminu Kano Teaching Hospital, Kano. Niger J Basic Clin Sci [Internet]. 2018;15(2):132–7. Available from: https://www.scopus.com/inward/record.uri?eid=2-s2.0-85168095669&doi=10.4103%2fnjbcs.njbcs_7_18&partnerID=40&md5=c7e1c748a50c8b49694551e897f3f4f5

126.	Zhang J, Wang Z, Leng H, Yang Z. Studies on lung injuries caused by blast underpressure. J Trauma - Inj Infect Crit Care [Internet]. 1996;40(3 SUPPL.):S77–80. Available from: https://www.scopus.com/inward/record.uri?eid=2-s2.0-0038211973&partnerID=40&md5=a4895208f211c16664a1f99d040ae953
