# Supplementary material for: Pattern of blast injuries. A Systematic Review: Part 1–Improvised, multiple and unspecified explosive devices
Source: Eur J Trauma Emerg Surg. 2026 Aug 24;52(1):266. doi: 10.1007/s00068-026-03261-x (PMC13503492; doi:10.1007/s00068-026-03261-x)
Supplement: Supplementary file 3 [file 68_2026_3261_MOESM3_ESM.docx]

**Additional file 4 - Risk of bias assessment**

|  | multiple & unspecified explosive devices (% positive vote) | IED (% positive vote) | landmines & UXO (% positive vote) | Terror including suicide bombing |
| --- | --- | --- | --- | --- |
| 1. | 94.5946 | 96.7742 | 83.3333 | 93.3333 |
| 2. | 89.1892 | 100 | 83.3333 | 95.5556 |
| 3. | 86.4865 | 96.7742 | 75 | 97.7778 |
| 4. | 97.2973 | 96.7742 | 91.6667 | 91.1111 |
| 5. | 97.2973 | 100 | 91.6667 | 91.1111 |
| 6. | 64.8649 | 54.8387 | 83.3333 | 66.6667 |
| 7. | 100 | 100 | 100 | 97.7778 |
| 8. | 100 | 100 | 100 | 100 |
| 9. | 86.4865 | 77.4194 | 91.6667 | 100 |
| 10. | 89.1892 | 100 | 83.3333 | 84.4444 |

1. Were there clear criteria for inclusion in the case series?
2. Were the blast injuries measured in a standard, reliable way for all participants included in the case series?
3. Were valid methods used for identification of the blast injuries for all participants included in the case series?
4. Did the case series have consecutive inclusion of participants?
5. Did the case series have complete inclusion of participants?
6. Was there clear reporting of the demographics of the participants in the study?
7. Was there clear reporting of clinical information of the participants?
8. Were the outcomes or follow up results of cases clearly reported?
9. Was there clear reporting of the presenting site(s)/clinic(s) demographic information?
10. Was statistical analysis appropriate?
