# Supplementary material for: Pattern of blast injuries. A Systematic Review: Part 1–Improvised, multiple and unspecified explosive devices
Source: Eur J Trauma Emerg Surg. 2026 Aug 24;52(1):266. doi: 10.1007/s00068-026-03261-x (PMC13503492; doi:10.1007/s00068-026-03261-x)
Supplement: Supplementary file 4 [file 68_2026_3261_MOESM4_ESM.docx]

# Additional file 5 – IED additional information

## A) Overview general injury pattern

| **Study ID** | **Demographic characteristics** | **Country**    **Time frame**    **Explosives** | **Severity of injuries** | **Injury by anatomical region** | | **Injuries reported**    Extremity injuries in detail:  **– nr. of persons with injury (%)** |
| --- | --- | --- | --- | --- | --- | --- |
| **Abdullahi 2024**    Retrospective hospital data    Children & Adults    Civilians & Military | **N=** 14    **Age:** ∅ 29.5 (±8.8; 14-43)  **Sex:** M 11 / F 3    **Multiple injuries:** 7 (50%)    **Mortality:**  **KIA: -**  **DOW:** 1 (7%) | Nigeria    **Time frame:**  2016-2022 | **Unknown** | **Nr. of injuries**    **Head:**  **Face:**  **Abdomen:**  **Thorax:**  **Extremity:**  **Spine:**  **Pelvis:**  **External incl. burns:**  **Urogenital:** | 73 (100%)    1 (1.2%)  31 (43%)  -  -  -  -  -    41 (56%)  - | ⵁ |
| **Ashworth 2023**    JTTR UK    Adults    Military | **N=** 46    **Age: -**  **Sex:** -    **Multiple injuries:**  46 (100%)    **Mortality:**  **KIA:**  **-**  **DOW:**  46 (100%) | Afghanistan & Iraq    **Time frame:**  2007-2013 | **Unknown** | **Nr. of injured persons:**    **Head:**  **Face:**  **Abdomen:**  **Thorax:**  **Extremity:**  **Spine:**  **Pelvis:**  **External:**  **Urogenital:** | 46 (100%)      46 (100%)  -  5 (11%)  26 (57%)  3 (6%)  17(37%)  -  -  -  - | - |
| **Ashworth 2024**    JTTR UK    Adults    Military | **N=** 71    **Age: -**  **Sex: -**    **Multiple injuries:**  55 (77%) of those with facial fractures    **Mortality:**  71 (100%)  **KIA:** -  **DOW: -** | Afghanistan & Iraq    **Time frame:**  2007-2013 | **Unknown** | **Nr. of reported injuries:**    **Head** (AIS ≥2)**:**  **Face:**  **Thorax:**  **Abdomen:**  **Extremity:**  **Spine:**  **Pelvis:**  **External:**  **Urogenital:** | 305 (100%)      173 (57%)  5 (1.6%)  41 (13%)  32 (10%)  -  54 (18%)  -  -  - | - |
| **Commandeur 2012**    Case series    Adults    Military | **N=** 8    **Age:-**  **Sex:** M 8 /F 0    **Multiple injuries:**  0 (0%)    **Mortality:**  **KIA:** -  **DOW: -** | Afghanistan    **Time frame:**  November 2008  –  January 2009 | **AIS ≥ 3** | **Nr. of injured persons:**    **Head/neck:**  **Face:**  **Thorax:**  **Abdomen:**  **Extremities:**  **Spine:**  **Pelvis:**  **External:**  **Urogenital:** | 8 (100%)      0 (0%)  0 (0%)  0 (0%)  0 (0%)  4 (50%)  2 (25%)  0 (0%)  0 (0%)  0 (0%) | **Upper extremity injuries:**  0 (0%)  **Lower extremity injuries:**  4 (50 %)    **Total nr. of amputations:**  0 (0%) |
|  |  |  | **AIS < 3** | **Nr. of injured persons:**    **Head/neck:**  **Face:**  **Thorax:**  **Abdomen:**  **Extremities:**  **Spine:**  **Pelvis:**  **External:**  **Urogenital:** | 8 (100%)      0 (0%)  0 (0%)  0 (0%)  0 (0%)  2 (25%)  0 (0%)  0 (0%)  2 (25%)  0 (0%) | **Upper extremity injuries:**  2 (25 %)    **Lower extremity injuries:**  0 (0%)    **Total nr. of amputations:**  0 (0%) |
| **Cross 2014**    Retrospective hospital data    Adults    Civilians & Military | **N=** 77    **Age: -**  **Sex:**  M 77 /F 0  **Multiple injuries:** 49 (64%)    **Mortality:**  **KIA: -**  **DOW:** 13 (17%) | Afghanistan    **Time frame:**  2009 - 2010 | **Unknown** | **Nr. of injured persons:**    **Head/neck:**  **Face:**  **Thorax:**  **Abdomen:**  **Extremities:**  **Spine:**  **Pelvis:**  **External:**  **Urogenital:** | 77 (100%)      -  -  -  -  77 (100%)  -  14 (18%)  -  - | **Nr. of injuries:**  123 (100%)    **Upper extremity injuries:**  -    **Lower extremity injuries:**  123 (100%)    **Total nr. of amputations:**  123 (100%) |
| **Dupaix 2018**    JTTR UK /US    Adults    Military | **N=** 499    **Age:**  ∅ 23.4 (±3.8)  **Sex:**  M 499 / F 0    **Multiple injuries:** -    **Mortality:**  **KIA: -**  **DOW:** - | Afghanistan    **Time frame:**  2009 - 2012 | **AIS ≥ 3** | **Nr. of injured persons:**    **Head:**  **Face:**  **Thorax:**  **Abdomen:**  **Extremities:**  **Spine:**  **Pelvis:**  **External:**  **Urogenital:** | 499 (100%)    -  -  -  -  295 (59%)  17 (3%)  39 (8%)  49 (10%)  - | **Nr. of injured persons:**  295 (100%)    **Nr. of persons with upper extremity injuries:**  23 (8%)    **Nr. of persons with lower extremity injuries:**  295 (100%)    **Total nr. of persons with amputations:**  201 (68%)  **Nr. of persons with bilateral lower limb amputations**:  140 (47%) |
| **Freedman 2014**    DoDTR/ JTTR    Adults    Military | **N=** 65    **Age:** ∅ 29.7 (±8.2)  **Sex:** M 63 / F 2    **Multiple injuries:** -    **Mortality:**  **KIA:** -  **DOW:** - | Afghanistan & Iraq    **Time frame:**  2007-2010 | **Unknown** | **Nr. of injured persons:**    **Head:**  **Face:**  **Thorax:**  **Abdomen:**  **Extremities:**  **Spine:**  **Pelvis:**  **External:**  **Urogenital:** | 65 (100%)    -  -  -  -  18 (28%)  65 (100%)  12 (19%)  -  - | **Nr. of injured persons:**  18 (100%)    **Nr of persons with upper extremity injuries:**  -    **Nr of persons with lower extremity injuries:**  18 (100%) |
| **Jacobs 2014**    Prospective cohort study    Adults    Civilian & military | **N**= 103    **Age:** -  **Sex:** -    **Multiple injuries:** -    **Mortality:**  **KIA:** -  **DOW:** 1 (1%) | Afghanistan    **Time frame:**  2010 - 2011 | **Unknown** | **Nr. of injured persons:**    **Head/neck:**  **Face:**  **Thorax:**  **Abdomen:**  **Upper Extremities**  **Lower**  **Extremities:**  **Spine:**  **Pelvis:**  **External:**  **Urogenital:** | 103 (100%)  -  -  -  11 (11%)    66 (64%)  103 (100%)  -  9 (9%)  -  41 (40%) | **Nr. of injuries:**  245 (100%)    **Lower extremity injuries:**  179 (73%)    **Total nr. of amputations:**  123 (50%) |
| **Mehta 2007**    Retrospective hospital data    Adults    Civilian | **N**= 16    **Age:** ∅ 35.3  (range: 22 - 52)  **Sex:** M 16 /F 0    **Multiple injuries:** -    **Mortality:**  **KIA: -**  **DOW:** - | India    **Time frame:**  not reported | **Unknown** | **Nr. of injured persons:**    **Head/neck:**  **Face:**  **Thorax:**  **Abdomen:**  **Extremities:**  **Spine:**  **Pelvis:**  **External incl. Burns:**  **Urogenital:** | 16 (100%)    -  16 (100%)  -  -  -  -  -  7 (44%)  - | - |
| **Miller 2022**    ITR & Israeli police database    Children & adults    Civilian | **N=** 74    **Age:** Mdn 32 (IQR 24, 42)  **Sex:** M 71 / F 3    **Multiple injuries:**  29 (39%)    **Mortality:**  **KIA: -**  **DOW:** 4 (5%) | Israel    **Time frame:**  January 2006 - July 2020 | **Unknown** | **Nr. of injured persons:**    **Head:**  **Neck:**  **Face:**  **Thorax:**  **Abdomen:**  **Upper Extremities:**  **Lower Extremities:**  **Spine:**  **Pelvis:**  **External incl. Burns:**  **Urogenital:** | 74 (100%)    13 (18%)  6 (8%)  25 (35%)  16 (23%)  21 (30%)  30 (42%)  42 (57%)  1 (1,4%)  -  22(31%)  - | **Nr. of injuries:**  72 (100%)    **Upper extremity injuries:**  30 (42%)    **Lower extremity injuries:**  42 (58%)    **Total nr. of amputations:**  27 (38%)   - 4 upper extremities - 23 lower extremities |
| **Minhas 2014**    Retrospective hospital data    Adult    Civilians | **N**= 70    **Age:**  20 – 32 years  **Sex:** 70 M/ F 0      **Multiple injuries:**  13 (19%)    **Mortality:**  **KIA:** 11 (16%)  **DOW:** 2 (3%) | Pakistan    **Time frame:**  February 13, 2014    **Explosive devices:**  IED | **Unknown** | **Nr. of injured persons:**    **Head/neck:**  **Face:**  **Thorax:**  **Abdomen:**  **Extremities:**  **Spine:**  **Pelvis:**  **External:**  **Urogenital:** | 70 (100%)    5 (7%)  4 (6%)  9 (13%)  6 (9%)  10 (14%)  -  -  25 (36%)  - | **Nr. of injuries:**  14 (100%)    **Upper extremity injuries:**  1 (7%)    **Lower extremity injuries:**  12 (86%)    **Total nr. of amputations:**  1 (7%) |
| **Morrison 2012**    JTTR UK    Adults    military | **N**= 169    **Age**: ∅ 24.0 (± 7)  **Sex:** M 168 / F 1    **Multiple injuries:**  -    **Mortality**  **KIA:** 69 (40%)  **DOW:** 16 (9%) | Afghanistan    **Time frame:**  2007 - 2010 | **AIS ≥ 3** | **Nr. of injured persons:**    **Head:**  **Face:**  **Thorax:**  **Abdomen:**  **Extremities:**  **Spine:**  **Pelvis:**  **External:**  **Urogenital:** | 169 (100%)    37 (22%)  -  49 (29%)  70 (41%)  169 (100%)  -  52 (31%)  -  - | **Nr of injuries:**  278 (100%)    **Nr. of upper extremity injuries:**  -    **Nr. of lower extremity injuries:**  278 (100%)    **Total nr. of amputations:**  278 (100%) |
| **Mossadegh 2012**    JTTR UK    Adults    Military | **N=** 118    **Age:** median 25 (IQR 21-29)  **Sex:** -    **Multiple injuries:** -    **Mortality:**  **KIA:** 35 (excluded from analysis)  **DOW:** 21 (18%) | Afghanistan    **Time frame:**  2003 -2010 | **Unknown** | **Nr. of injured persons:**  **Head:**  **Face:**  **Thorax:**  **Abdomen:**  **Extremities:**  **Spine:**  **Pelvis:**  **External:**  **Urogenital:** | 118 (100%)    -  -  -  -  -  -  62 (53%)  -  85 (72%) | - |
| **Nelson 2007**    Case reports    Adults    Military | **N=** 18    **Age: -**  **Sex: -**      **Multiple injuries:**  15 (83%)    **Mortality:**  **KIA:** 4 (22%)  **DOW:** 5 (28%) | Iraq    **Time frame:**  August 2004  &  September 2004 | **Unknown** | **Nr. of injured persons:**    **Head/neck:**  **Face:**  **Thorax:**  **Abdomen:**  **Extremities:**  **Spine:**  **Pelvis:**  **External:**  **Urogenital:** | 18 (100%)    8 (44%)  1 (6%)  2 (11%)  5 (28%)  9 (50%)  2 (11%)  1 (6%)  6 (33%)  - | **Nr. of injuries:**  18 (100%)    **Upper extremity injuries:**  6 (33 %)    **Lower extremity injuries:**  11 (61%)    **Total nr. of amputations:**  1 (6%) |
| **Oh 2016**    JTTR US & UK    Adults    Military | **N=** 457    **Age:** Median 23 (IQR 21- 26)  **Sex:** M 456 / F 1    **Multiple injuries:** 116 (25%)    **Mortality:**  **KIA: -**  **DOW:** 8 (2%) | Afghanistan    **Time frame:**  2009 -2012 | **AIS ≥ 3** | **Nr. of injured persons:**    **Head:**  **Face:**  **Thorax:**  **Abdomen:**  **Extremities:**  **Spine:**  **Pelvis:**  **External:**  **Urogenital:** | 457 (100%)    23 (5%)  -  10 (2%)  21 (5%)  151 (35%)  -  34 (7%)  4 (1%)  - | **Nr. of injuries:**  303 (100%)    **Upper extremity injuries:**  11 (4%)    **Lower extremity injuries:**  292 (96%)    **Total nr. of amputations:**  303 (100%) |
| **Pearce 2017**    JTTR UK    Adults    Military | **N=** 426    **Age:** ∅ 24 (21–29)  **Sex:**  M 419 / F7    **Multiple injuries:** -    **Mortality:**  129 (30%) | Afghanistan & Iraq    **Time frame:**  2003-2014 | **AIS ≥ 3** | **Nr. of injuries:**    **Head:**  **Face:**  **Thorax:**  **Abdomen:**  **Extremities:**  **Spine:**  **Pelvis:**  **External:**  **Urogenital:** | 657 (100%)    124 (19%)  -  306 (47%)  227 (35%)  -  -  -  -  - | - |
| **Penn-Barwell 2014**    JTTR UK    Adults    Military | **N=** 43    **Age:** median 25  (IQR 22–28)  **Sex:** M 43 / F 0    **Multiple injuries:** 43 (100%)    **Mortality:**  **KIA:** -  **DOW:** - | Afghanistan    **Time frame:**  March 2004 –  March 2010 | **Unknown** | **Nr. of injured persons:**    **Head:**  **Face:**  **Thorax:**  **Abdomen:**  **Extremities:**  **Spine:**  **Pelvis:**  **External:**  **Urogenital:** | 43 (100%)  -  -  -  -  43 (100%)  -  6 (14%)  -  19 (44%) | **Nr. of injuries:**  95 (100%)    **Upper extremity injuries:**  9 (10 %)    **Lower extremity injuries:**  86 (91%)    **Total nr. of amputations:**  95 (100%) |
| **Ragel 2009**    Retrospective hospital data    Adults    Military | **N=** 19    **Age:** ∅ 30.7 (20-43)  **Sex:** M 19 / F 0    **Multiple injuries:**  9 (47%)    **Mortality:**  **KIA: -**  **DOW:** - | Afghanistan    **Time frame:**  Jan – May 2008 | **Unknown** | **Nr. of injured persons:**    **Head:**  **Face:**  **Thorax:**  **Abdomen:**  **Extremities:**  **Spine:**  **Pelvis:**  **External:**  **Urogenital:** | 19 (100%)    -  1 (5%)  5 (26%)  3 (15%)  16 (84%)  12 (63%)  2 (10%)  -  - | **Nr. of injuries:**  27 (100%)    **Upper extremity injuries:**  5 (19%)    **Lower extremity injuries:**  22 (81%)    **Total nr. of amputations:**  2 (7%) |
| **Ramasamy 2008**    Retrospective hospital data  Civil and military | **N=** 53    **Age:** -  **Sex:** -    **Multiple injuries:**  -    **Mortality:**  **KIA:** 11 (21%)  **DOW:** 1 (2%) | Afghanistan & Iraq    **Time frame:**  January 2006- October 2006 | **Unknown** | **Nr. of injuries:**    **Head/neck:**  **Face:**  **Thorax/Back:**  **Abdomen:**  **Extremities:**  **Spine:**  **Pelvis:**  **External incl.**  **burns:**  **Urogenital:** | 171 (100%)    28 (16%)  19 (11%)  15 (9%)  5 (3%)  89 (54%)  -  7 (4%)    8 (5%)  - | **Nr. of injuries:**  89 (100%)    **Upper extremity injuries:**  46 (51%)    **Lower extremity injuries:**  43 (48%)    **Total nr. of amputations:**  9 (10%) |
| **Ramasamy 2012**    JTTR UK    Adults    Military | **N= 29**    **Age:** ∅ 25.5 (18-32) (survivors)  **Sex:** M 89 / F 0    **Multiple injuries:** 88 (99%)    **Mortality:**  60 (67%) | Afghanistan & Iraq    **Time frame:**  2008 -2010 | **Unknown** | **Nr. of injuries:**    **Head/Face:**  **Thorax:**  **Abdomen:**  **Extremities:**  **Spine:**  **Pelvis:**  **External:**  **Urogenital:**  **Vascular pelvic region:** | 267 (100%)    14 (5%)  16 (6%)  19 (7%)  81 (30%)  16 (6%)  29 (10%)  76 (29%)  11 (4%)    6 (2%) | **Nr. of injuries:**  81 (100%)    **Upper extremity injuries:**  22 (27%)    **Lower extremity injuries:**  59 (73%)    **Total nr. of amputations:**  10 (12%) |
| **Rankin 2020**    JTTR UK    Adults    Military and civilian | **N=** 307    **Age: -**  **Sex:** -    **Multiple injuries: -**    **Mortality:**  **KIA:** 36 (23%)  **DOW:** 22 (14%) | Afghanistan & Iraq    **Time frame:**  2003 - 2014 | **AIS >3**  **(only the most severe injury region reported)** | **Nr. of injured persons:**    **Head:**    **Face:**  **Thorax:**  **Abdomen:**  **Extremities:**  **Spine:**  **Pelvis:**    **External:**  **Urogenital:** | 307 (100%)    75 (47%)  -  27 (17%)  17 (11%)  108 (68%)  13 (8%)  159 (100%)  -  - | **Nr. of injured persons:**  108 (100%)      **Total nr. of persons with amputations:**  108 (100%) |
| **Salinas 2011**    Retrospective hospital data    Adults    Military | **N=** 104    **Age:** ∅ 26.8  **Sex:** -    **Multiple injuries: -**    **Mortality:**  **KIA: -**  **DOW:** - | Iraq    **Time frame:**  2004 - 2007 | **Unknown** | **Nr. of injured persons:**    **Head:**  **Face:**  **Thorax:**  **Abdomen:**  **Extremities:**  **Spine:**  **Pelvis:**  **External:**  **Urogenital:** | 104 (100%)    35 (34%)  104 (100%)  -  -  -  -  -  -  - | - |
| **Singleton 2013**    JTTR UK    Adults    Military | **N=** 121    **Age:** median 25 (IQR 21–29)  **Sex:** M 120 /F 1    **Multiple injuries:**  121 100%    **Mortality:**  **KIA:** 108 (89%)  **DOW:** 13 (11%) | Afghanistan& Iraq    **Time frame:**  November 2007  -  August 2010 | **Unknown** | **Nr. of injuries:**    **Head/neck:**  **Face:**  **Thorax:**  **Abdomen:**  **Extremities:**  **Spine:**  **Pelvis:**  **External:**  **Burns:**  **Urogenital:** | 354 (100%)    119 (34%)  2 (0.5%)  47 (13%)  41 (12%)  128 (36%)  12 (3%)  -  -  -  - | **Nr. of injuries:**  128 (100%)    **Upper extremity injuries:**  8 (6.25%)    **Lower extremity injuries:**  120 (93.75%)    **Total nr. of amputations:**  - |
| **Smith 2017**    Prospective cohort study    Children & Adults    Military & civilian | **N=** 100    **Age:**  ∅ 25 (range 6 – 44)  **Sex:** M 100 / F 0    **Multiple injuries:** 81 (81%)    **Mortality:**  **KIA:** 11 (11%)  **DOW:** 8 (8%) | Afghanistan    **Time frame:**  2010 – 2011 | **Unknown** | **Nr. of injured persons:**    **Head:**  **Face:**  **Thorax:**  **Abdomen:**  **Extremities:**  **Spine:**  **Pelvis:**  **External:**  **Urogenital:** | 100 (100%)    12 (12%)  24 (24%)  -  -  100 (100%)  -  21 (21%)  -  46 (46%) | **Nr. of injuries:**  207 (100%)    **Upper extremity injuries:**  43 (21%)    **Lower extremity injuries:**  130 (63%)    **Total nr. of amputations:**  207 (100%) |
| **Spurrier 2015**    JTTR UK    Adults    Military | **N=** 78    **Age:** ∅ 26.8  (range 18–55)  **Sex:** -    **Multiple injuries:** -    **Mortality:**  **KIA:** 13 (17%)  **DOW**:12 (15%) | Afghanistan & Iraq    **Time frame:**  2008 - 2013 | **Unknown** | **Nr of injured persons:**    **Head:**  **Face:**  **Thorax:**  **Abdomen:**  **Extremities:**  **Spine:**  **Pelvis:**  **External:**  **Urogenital:** | 78 (100%)    -  -  -  -  -  78 (100%)  -  -  - | - |
| **Staruch 2017**    JTTR UK    Adults    Military | **N= 380**    **Age: -**  **Sex:** -    **Multiple injuries: -**    **Mortality:**  **KIA: -**  **DOW: -** | Afghanistan    **Time frame:**  2006 - 2013 | **Unknown** | **Nr. of injured persons:**    **Head:**  **Face:**  **Thorax:**  **Abdomen:**  **Extremities:**  **Spine:**  **Pelvis:**  **External:**  **Urogenital:** | 380 (100%)    -  -  -  -  380 (100%)  -  -  -  - | **Nr. of injured persons:**  380 (100%)    **Nr. of persons**  **with upper extremity injuries:**  380 (100%)    **Nr. of persons with lower extremity injuries:**  -    **Total nr. of amputations:**  114 (30%) |
| **Stewart 2018**    JTTR UK    Adults    Military | **N=** 94    **Age:** ∅ 25  **Sex:** M 92 / F 2    **Multiple injuries:**  62 (71,3%)    **Mortality:**  94 (100%) | Afghanistan & Iraq    **Time frame:**  2003 - 2014 | **Unknown** | **Nr. of injuries:**    **Head:**  **Face:**  **Thorax:**  **Abdomen:**  **Extremities:**  **Spine:**  **Pelvis:**  **External:**  **Urogenital:** | 197 (100%)    181 (92%)  -  -  -  -  16 (8%)  -  -  - | - |
| **Taddeo 2015**    JTTR UK + KAF patient tracking data base    Adults    Military | **N=** 326    **Age:** -  **Sex:** -    **Multiple injuries:** 22 (7%)    **Mortality:**  **KIA:** -  **DOW:** - | Afghanistan    **Time frame:**  2008 -2011 | **Unknown** | **Nr. of injured persons:**    **Head/neck:**  **Face:**  **Thorax:**  **Abdomen:**  **Extremities:**  **Spine:**  **Pelvis:**  **External:**  **Burns:**  **Urogenital:** | 326 (100%)    66 (20%)  4 (1.2%)  -  -  96 (29%)  19 (20%)  -  -  -  - | **Nr. of injured persons:**  96 (100%)    **Nr. of persons**  **with upper extremity injuries:**  12 (12.5%)    **Nr. of persons with lower extremity injuries:**  92 (96%)    **Total nr. of amputations:**  96 (100%) |
| **Tahtabasi 2021 (b)**    Retrospective hospital data    Adults    Civilian | **N**= 101    **Age:**  ∅ 32.2 (±12.7)  **Sex:** M 76 / F 25    **Multiple injuries:**  33 (32.7%)    **Mortality:**  **KIA: -**  **DOW:** 13 (13%) | Somalia    **Time frame:**  January 2019  -  December 2019 | **Unknown** | **Nr. of injured persons:**    **Head/neck:**  **Face:**  **Thorax:**  **Abdomen:**  **Extremities/ Pelvis:**  **Spine:**  **External:**  **Vascular:**  **Urogenital:** | 101 (100%)    12 (12%)  -  17 (17%)  17 (17%)  76 (75%)  -  25 (25%)  11 (11%)  - | **Nr. of injuries:**  103 (100%)    **Upper extremity injuries:**  42 (41%)    **Lower extremity injuries:**  59 (57%)    **Total nr. of amputations:**  7 (7%) |
| **Ünlü 2015**    Retrospective hospital data    Adults    Civilians & military | **N**= 133    **Age:** -  **Sex:** M 133 / F 0    **Multiple injuries:**  56 (42%)    **Mortality:**  **KIA:** -  **DOW:** - | Iraqi border of Turkey    **Time frame:**  July 2005  –  June 2007 | **Unknown** | **Nr. of injured persons:**    **Head/neck:**  **Face:**  **Thorax:**  **Abdomen:**  **Extremities:**  **Spine:**  **Pelvis:**  **External incl. burns:**  **Urogenital:** | 133 (100%)    6 (5%)  20 (15%)  5 (4%)  14 (11%)  33 (24%)  -  -  56 (42%)  13 (10%) | **Nr. of injuries:**  33 (100%)    **Upper extremity injuries:**  6 (19%)    **Lower extremity injuries:**  8 (24%)    **Total nr. of amputations:**  19 (57%) |
| **Webster 2018**    JTTR UK    Adults    Military & civilian | **N=** 342    **Age:** Mdn 29 (range 18–51)  **Sex:** M 338 / F 4    **Multiple injuries:** -    **Mortality:**  **KIA: -**  **DOW:** 165 (48%) | Afghanistan & Iraq    **Time frame:**  2003 - 2014 | **Unknown**  **severity** | **Nr. of injured persons:**    **Head/Neck/Face:**  **Thorax:**  **Abdomen:**  **Extremities:**  **Spine:**  **Pelvis:**  **External:**  **Urogenital:** | 342 (100%)    66 (19%)  36 (11%)  18 (5%)  205 (60%)  98 (29%)  325 (95%)  -  - | **Nr. of injuries:**  205 (100%)    **Upper extremity injuries:**  2 (1%)    **Lower extremity injuries:**  203 (99%)    **Total nr. of amputations:**  185 (90%) |

*Legend: DOW = died of wounds; F = Female; KIA = killed in action; M = male; N = sample size; nr. = number; y= years; - = not reported; ∅ = mean; ± = standard deviation; Color indicates the same data source: JTTR UK/USA*

## B) Focus on specific body parts

Extremity injuries including amputations were the focus of the eight of the included studies (extremities: Staruch 2917, Jacobs 2014, Smith 2017, amputations: Cross 2014, Dupaix 2018, Oh 2016, Morrison 2012, Penn-Barrell 2014).

All but one study on amputations is based on JTTR data from the UK and/or the US. Their populations due to overlap in definition of injuries and time frame are probably overlapping as well as all investigating military personnel. Only Cross (2014) investigates hospital data but also their data derive from a UK military hospital. Hence, their data probably overlaps with those of the JTTR register. They only differ in focus – only lower extremity, only bilateral amputations etc. The studies found a

Cross (2014) found a high incidence of concurrent pelvic fractures in traumatic amputees, with an even higher incidence in those with bilateral amputations above the knee. Those with pelvic injuries mostly suffered a combined mechanism of antero-posterior compression and vertical shear.

Dupaix (2018) on the other hand reports on an association of multiple extremity injuries with penetrating pelvic and abdominal trauma, urogenital injuries and upper extremity injuries in dismounted military patrols in Afghanistan. They generally noted a high number of multiple extremity injuries and discuss that this could be related to the population of mainly marine patrols they treated who are mainly injured by a dismounted injury mechanism. Further, they demonstrated an association between single lower leg amputation with upper extremity injuries (DUPAIX). Oh (2016) found in their cohort of military personnel who were injured by dismounted IEDs, an association between mortality and the presence of two or more limb amputations and the presence of pelvic fractures but they did not include those who were KIA nor those you died in Role 2. Most of their patients suffered extremity and/or pelvic injuries. In relation to the pelvic fractures – most of their patients had unstable (Tile b or C) open fractures. Morrison (2011) add that in their cohort especially those patients with hindquarter amputations (n=39) had a high risk of mortality. Penn-Barrell (2014) reported exclusively on bilateral and triple amputations and showed that in their cohort of 43 Uk servicemen nine also sustained open pelvic fracture. Additionally, 19 patients had an injury to their urogenital tract. 9 patients suffered a triple amputation of two lower extremities and one upper extremity (trans-radial or trans-humeral). No patient with four limb amputations survived in their cohort (Penn-Barrel 2014).

The three studies on pelvic fractures show a wide range of different pelvic fractures (Antero-posterior compression, lateral compression, vertical shear as well as other fractures) – also combinations were reported. Also in these studies, populations are probably overlapping due to the use of UK JTTR register as basis of all three studies with the widest time frame by Webster (2019) who investigated the years 2004 until 2014 with a military and civil population in Afghanistan (+/- Iraq). The other two studies only investigated military personnel. Mossadegh (2012) supports the studies on extremity injuries that in Iraq and Afghanistan high numbers of combined injuries of high transfemoral amputations, pelvic injuries and (maiming) perineal (urogenital) injuries occurred. Webster (2019) shows that dismounted casualties are most often suffer unstable pelvic fractures in combination with traumatic amputations of which they often die due to exsanguination. The focus should, therefore, be on immediate hemorrhage control of the lower extremities and pelvic. Wounds of dismounted casualties are often highly contaminated with fragments and soil. They propose three possible mechanisms (1) axial load via femoral head from directly standing on the IED, (2) flail of the lower limb, and (3) blast wind and fragmentation.

Four studies investigated mounted casualties and one study on dismounted casualties with spinal injuries. Spurrier and colleagues (2015) investigate several different types of spinal fractures of the entire spine including burst and Chance fractures. These two different types were analyzed separately by Freedman (2014) and Ragel (2009), respectively for thoracolumbar fractures. Spurrier (2015) reports that thoracic fractures often appear at T4 to T6 whereas lumbar fractures are often seen at L1, but in general lumbar fractures were very common in their cohort (47% of all spinal fractures).

Freedman (2014) reported on the increase in thoracolumbar burst fractures with/without spinal cord injuries, especially in relation to IED incidents in up-armored military vehicles. The authors state that these injuries often occur in combination with other combat injuries. Ragel and colleagues (2009) report on the incidence of thoracolumbar Chance fractures (flexion distraction fractures) due to hyperflexion of the thoracolumbar spine of vehicle occupants during an IED explosion. Two different injury mechanisms are proposed: (1) sudden acceleration from below (like in fighter jet ejection) and (2) sudden deceleration (like in a helicopter crash).

Spurrier (2015) add cervical fractures are possible due to a head strike to the inside of the vehicle or due to personal equipment being pushed upwards and strikes the head. They add that this is in line with the increasing risk for head injuries in those with C1 to C7 fractures. However, other scenarios are likely, too, e.g. cervical spine buckling under axial load or a combination of buckling of the cervical spine and head struck (favored by Spurrier 2015). Additional Taddeo (2015) showed that cervical fractures are rare in dismounted trauma.

In addition to these findings, two studies exclusively investigated head and neck / spinal injuries due to a mounted injury mechanism (underbody blast) in fatalities of military personnel deployed in Afghanistan and Iraq (20,42). Both use the UK JTTR as data source. Due to the overlap in investigated years, both analyze the same or very similar population. Stewart (2019) is only focusing on head and neck injuries (42) whereas Ashworth (2023) is also including all other spinal injuries (20). The former found that skull, neck and spine are vulnerable to tertiary blast forces which are related to injuries caused by flexion/extension, rotation, and shear. Ashworth (2023) further found eggshell skull fracture related to high energy direct impact on the skull. Stewards (2019) found that high energy head injuries are most common in fatalities who suffered a mounted injury mechanism, but these fatal head injuries seem not to be mechanically related to the observed cervical spine injuries (42). Authors of both studies state that vehicle design and protection equipment such as helmet design could protect from several of these injuries.

Two studies investigated injuries to the face. Salinas focuses on IED incidents with massive facial injuries treated in an Airforce hospital in Iraq during 2004 and 2007. They found that massive facial injuries are more common at forehead, right and left cheeks, nose, and lips/mouth and that these injuries may be related to a higher ISS and a higher mortality (SALINAS).

The findings of Mehta (2007) differ from those of Salinas (2011) due to the special environment, commuter train in India, in which der IED detonated. They report rather minor injuries that were not vision threatening. The authors state that this is probably due to IED design which mainly caused overpressure, but less shrapnel injuries. Additionally, Abdullahi 2024 report on maxillofacial injuries in Nigeria due to BukoHaram IEDs.

| **Focus** | **Study ID** | **Severity of injuries** | **Specifics of the Injuries**  **(%)** | |
| --- | --- | --- | --- | --- |
| **Head & neck** | **Ashworth 2023** | **Unknown** | **Nr of persons with head injuries**   - subarachnoid - Skull fracture - Contusion - Tightness - Acute subdural - Extradural - diffuse axonal injury - Hypoxia   **Nr of persons with spine injuries**   - spinal fractures of the lumbar - sacral spine region - thoracic spine region - cervical-spine region - atlanto-occipital fractures (C0-C1) | **92**  37 (80%)  29 (63%)  13 (28%)  7 (15%)  3 (7%)  2 (4%)  1 (2%)  0 (0%)    **90**  24 (52%)  23 (50%)  22 (48%)  21 (46%)  14 (30%) |
|  | **Ashworth 2024** | **AIS ≥2** | **Nr. of injured persons with head injuries:**   - Extradural head injury - Acute subdural - Chronic subdural - Subarachnoidal - Contusion - Diffuse axonal injury - Hypoxia - Tightness - Skull fracture | **71**  1 (1%)  5 (7%)  0 (0%)  31 (44%)  13 (18%)  0  0  6 (8%)  59 (83%) |
|  | **Stewart 2019** | **Unknown** | **Nr. of injuries**    **Nr of head injuries**   - skull vault injury - parenchymal brain injury - basal skull fracture - Brain stem injury   **Nr of spine injuries**   - cervical spine fracture | **197**    **94**  48 (55%)  65 (75%)  40 (46%)  28 (32%)    16 (18%) |
| **Facial & head injuries** | **Abdullahi 2024** | **Unknown** | **Nr. of injuries:**  **Soft tissue injuries:**   - Bruns - Abrasion - Contusion - Laceration - Avulsion   **Hard tissue injuries:**   - Dento-alveolar fracture - Maxillary - Mandibular - Zygomatic complex - Naso orbital ethmoidal - Frontal - Pan facial | **73 (100%)**  **41 (56%)**  6 (8%)  9 (12%)  5 (7%)  13 (18%)  8 (11%)  **32 (44%)**  4 (5%)  6 (8%)  4 (5%)  3 (4%)  7 (10%)  5 (7%)  3 (4%) |
|  | **Salinas 2011** | **Massive head & facial trauma** | **Nr. of injured persons with massive face injury:**    **Nr. of injuries**   - Brain injuries - Eye injuries - Forehead - Right Cheek - Left Cheek - Nose - Lip/ mouth - Chin - Right Ear - Left Ear - Right globe - Left globe - Frontal Sinus - Maxilla - Zygoma - Mandible - Orbit - Nasal bones | **29 (28%)**    **109**  14 (13%)  16 (15%)  8 (7%)  8 (7%)  7 (6%)  7 (6%)  6 (6%)  6 (6%)  3 (3%)  3 (3%)  5 (5%)  6 (6%)  2 (2%)  6 (6%)  2 (2%)  8 (7%)  5 (5%)  2 (2%) |
|  |  | **Simple head & facial trauma** | **Nr of injured persons with**   - **Simple face injuries** - **Head injuries**   **Nr. of injuries**   - Brain injury - Eye injuries - Forehead - Right Cheek - Left Cheek - Nose - Lip/ mouth - Chin - Right Ear - Left Ear - Right globe - Left globe - Frontal Sinus - Maxilla - Zygoma - Mandible - Orbit - Nasal bones | **75** (72%)  **35** (34%)    **116**  21 (18%)  20 (17%)  4 (3%)  7 (6%)  5 (4%)  4 (3%)  4 (3%)  5 (4%)  2 (2%)  2 (2%)  9 (8%)  6 (5%)  2 (2%)  8 (7%)  1 (1%)  10 (9%)  5 (4%)  1 (1%) |
| **Eye injuries** | **Mehta 2007** | **AIS >3** | **Nr. of injured persons**  **Face:** | **16** (89%) |
|  |  |  | **Total nr. of injuries:**    **Total nr of injured eyes**   - Periorbital haemorrhage - Lid burns - Corneal injuries - Corneoscleral tears - Traumatic optic neuropathy | **27**    **22 (6 bilateral)**  9 (41%)  7 (32%)  8 (36%)  2 (9%)  1 (5%) |
| **Thorax & Abdomen** | **Pearce 2017** | **AIS >3** | **Total nr. of thorax injuries**   - Cardiac injury - Thoracic aorta Injury - Pulmonary artery Injury - Pulmonary vein Injury - SVC injury - Hemothorax - Pneumothorax - Lung contusion - Blast lung - Lung laceration - Other thoracic vascular injury - Rib/Sternal Fractures     **Total nr of abdomen injuries**   - Liver injury - Splenic injury - Kidney injury - Abdominal aorta Injury - IVC Injury - Other abdominal vascular injury - GI Injury | **222**  32 (14%)  17 (8%)  1 (0.5%)  4 (2%)  2 (1%)  40 (18%)  13 (6%)  54 (24%)  23 (10%)  26 (12%)  5 (2%)  5 (2%)    **318**  59 (19%)  63 (20%)  38 (12%)  2 (1%)  2 (1%)  22 (7%)  99 (31%) |
| **Upper extremity injuries** | **Staruch 2017** | **Unknown** | **Total Nr. of injured persons with upper extremity injury**  **Hand:**   - Metacarpal, phalanges or digital amputation - Injuries on both hands | **380**    103 (27%)  18 (5%) |
|  |  |  | **Nr. hand of injuries**   - metacarpal (MC) fractures - phalangeal fractures - phalangeal amputation | **380**  72 (19%)  56 (15%)  114 (30%) |
| **Lower extremity injuries** | **Cross 2014** | **Unknown** | **Nr. of injured persons: with lower extremity injuries**   - Pelvis - At least 2 limb amputations - unilateral BKA - Unilateral AKA - bilateral AKA - bilateral either BKA, or mixed AKA/BKA   **Total nr of extremity amputation** | **77**  17 (22%)  46 (60%)  17 (22%)  14 (18%)  28 (36%)  18 (23%)  **123** |
|  | **Jacobs 2014** | **Unknown** | **Nr of injured persons with**   - Upper extremity injury - Pelvis - Urogenital - Bilateral lower limb injury - Bilateral lower limb amputation   **Nr. of injuries:**   - Extremity injuries - Lower extremity injury   Traumatic Amputations (lower extremity) | **103**  66 (64%)  9 (9%)  41 (40%)  76 (74%)  50 (49%)    245  179    123 |
|  | **Smith 2017** | **Unknown** | **Nr of injured persons with**   - Pelvis injuries - Urogenital injuries     **Total nr. patients with amputations**   - Multiple amputations      - At least one AKA - At least one BKA - Bilateral lower extremity amputations - At least one upper extremity amputation   **Nr. of injuries**   - Total nr. of amputations - Number of upper extremity injuries | **100**  9 (9%)  46 (46%)    **100**  70 (70%)    41 (41%)  56 (56%)  65 (65%)  37 (37%)    207  43 |
| **Amputations** | **Dupaix 2018** | **AIS >3** | **Nr of injured persons**   - Upper extremity - Lower Extremity - Traumatic lower extremity injury - Bilateral extremity amputation - Single leg amputation | **499**  23 (5%)  295 (59%)  201 (40%)  140 (28%)  61 (12%) |
|  |  |  | **Nr. of injuries**   - Femur - Tibial fractures - Ankle Fractures - Hindfoot fractures - Midfoot fractures - Forefoot fractures - Hindfoot level - Trough ankle - BKA - Trough the knee amputation - AKA | **245**  7 (3%)  17 (7%)  42 (17%)  60 (24%)  37 (15%)  38 (16%)  4 (2%)  2 (1%)  29 (12%)  2 (1%)  7 (3%) |
|  | **Oh 2016** | **AIS >3** | **Nr of injured persons**  Pelvis  Limb amputation    **Number of injuries**   - Upper extremity - Extremity amputation - Upper extremity amputation - Lower extremity amputation | **457**  34 (7%)  172 (38%)    **647**  41 (6%)  303 (47%)  11 (2%)  292 (45%) |
|  | **Morrison 2012** | **AIS >3** | **Nr. of injuries**   - **traumatic lower extremity amputation**   **Nr. of injured persons:**  **Bilateral amputation**   - Hindquarter & Hindquarter - Hindquarter & AKA - Hindquarter & BKA - Hindquarter  AKA & AKA - AKA & BKA - AKA - BKA & BKA - BKA | **278**  **169**  **115**  12 (4%)  14 (5%)  7 (3%)  6 (2%)  40 (14%)  20 (7%)  20 (7%)  22 (8%)  28 (10 %) |
|  | **Penn-Barwell 2014** | **Unknown** | **Lower bilateral extremity amputations:**   - bilateral trans-femoral amputation - bilateral trans-tibial amputations transtibial/knee articulation - transtibial and trans femoral - knee disarticulation and trans-femoral   **including those with triple amputation including upper extremity amputations:**  - trans-radial amputation  - trans-humeral amputation | **43**  25 (29%)  4 (5%)  1 (1%)  4 (5%)    **9**  3 (33%)  6 (66%) |
| **Pelvic injuries** | **Mossadegh 2012** | **Unknown** | **Nr of patients with pelvic injuries**  - LC-III  - APC grade I  - APC grade II  - APC grade III  - VS  - CM  - other | **62**  1 (2%)  3 (5%)  19 (31%)  15 (24%)  4 (6%)  3 (5%)  17 (27%) |
|  | **Ramasamy 2012** | **Unknown** | **Nr of patients with pelvic fractures**  **-** Tile A  - Tile B  - Tile C  - unclassified | **28**  7 (25%)  7 (25%)  6 (21%)  9 (32%) |
|  | **Webster 2019** | **Unknown** | **Nr of pelvic injuries**  - pubic symphysis disruption  - sacroiliac joint disruption  - pubic ramus fracture  - sacral fracture  - acetabular fracture | **708**  179 (25%)  185 (26%)  138 (20%)  108 (15%)  98 (14%) |
| **Spinal injuries** | **Freedman 2014** | **Unknown** | **Nr of patients with thoracolumbar injuries**   - multi-level burst fractures in TL region - additional low lumbar or thoracic spine injuries | **65**  3 (5%)  5 (8%) |
|  | **Ragel 2009** | **Unknown** | **Nr. of patients with spinal injuries**    **Nr. of injuries in spinal region**  - Nr. of thoracolumbar fractures in 3 persons  - Nr. of cervical fractures | **12**    **17**  16 (94%)  1 (6%) |
|  | **Spurrier 2015** | **Unknown** | **Nr of spine fractures**  - cervical fracture  - thoracic fracture  - lumbar fracture  **Nr. of spinal fractures**  - C1  - C2  - C3  - C4  - C5  - C6  - C7  - T1  - T2  - T3  - T4  - T5  - T6  - T7  - T8  - T9  - T10  - T11  - T12  - L1  - L2  - L3  - L4  - L5 | **78**  21 (27%)  42 (54%)  55 (71%)  277  4 (1%)  6 (2%)  3 (1%)  3 (1%)  3 (1%)  8 (3%)  12 (4%)  8 (3%)  5 (2%)  6 (2%)  10 (4%)  12 (4%)  12 (4%)  9 (3%)  9 (3%)  11 (4%)  11 (4%)  7 (3%)  13 (5%)  32 (12%)  27 (10%)  28 (10%)  22 (8%)  16 (6%) |
|  | **Taddeo 2015** | **Unknown** | **Nr of patients with C-spine fractures**  - unstable cervical fractures  - pedicle fracture  - multiple, 3 column fracture  - body fracture  - stable cervical fractures  - transverse process  - laminar fractures  - spinous process  - anterior chip  - endplate  - facet  - peripheral fracture | **19**    2 (11%)  1 (5%)  1 (5%)  5 (26%)  3 (16%)  2 (11%)  1 (5%)  1 (5%)  1 (5%)  1 (5%) |
| **Vascular injuries** | **Rankin 2020** | **Unknown** | **Nr of pelvic vascular injuries**  - venous  - arterial | **103**  55 (53%)  48 (46%) |

*Legend: AKA = Above knee amputation; APC = antero-posterior compression; BKA = below knee amputation; C = cervical; CM = combined mechanism; GI = gastro-intestinal LC = lateral compression; L = lumbar; nr. = number; T = thoracal; TL region = thoracic-lumbar region; VS = vertical shear; Color indicates the same data source: JTTR UK/USA*
