# Supplementary material for: Pattern of blast injuries. A Systematic Review: Part 1–Improvised, multiple and unspecified explosive devices
Source: Eur J Trauma Emerg Surg. 2026 Aug 24;52(1):266. doi: 10.1007/s00068-026-03261-x (PMC13503492; doi:10.1007/s00068-026-03261-x)
Supplement: Supplementary file 5 [file 68_2026_3261_MOESM5_ESM.docx]

# Appendix X – Additional information multiple and unspecified explosive devices

## A) Overview general injury pattern

| **Study ID** | **Demographic characteristics** | **Country**    **Time frame**    **Explosives** | **Severity of injuries** | **Injury by anatomical region** | | **Injuries reported**    Extremity injuries in detail: |
| --- | --- | --- | --- | --- | --- | --- |
| **Bashir 2013**    Retrospective  hospital data    Children & adults    Civilians | **N**= 16    **Age:** ⵁ 29  (range 4-58)  **Sex:** M 14 / F 2    **Multiple injuries:** -    **Mortality:**  **KIA:** 0 (0%)  **DOW:** 0 (0%) | Pakistan    **Time frame**  5 years    **Explosives**  suicidal blasts & implanted devices | **Unknown severity** | **Nr. of injured persons:**    **Head**  **Face**  **Thorax**  **Abdomen**  **Extremities**  **Spine**  **Pelvis**  **External**  **Urogenital** | 16 (100%)  16 (100%)  -  -  -  -  -  -  -  - | - |
| **Belmont 2011**    JTTR US    Adults    Military | **N**= 142    **Age:** -  **Sex:** -    **Nr. of multiple injured:**  11 (0.2%)    **Mortality**  **KIA:** -  **DOW:** 2 | Iraq    **Time frame:**  January 2007 to July 2008    **Explosive devices:**  multiple | **Unknown severity** | **Nr. of injuries:**    **Head**  **Face**  **Thorax**  **Abdomen**  **Extremities**  **Spine/ Pelvis**  **External**  **Neurovascular Injuries** | 199 (100%)    -  -  -  -  48 (24%)  10 (5%)  133 (67%)    8 (4%) | **Nr. of injuries:**  48 (100%)    **Upper extremity injuries:**  18 (38%)    **Lower extremity injuries:**  13 (27%)    **Total nr. of amputations:**  14 (31%) |
| **Belmont 2013**    JTTR US    Adults    Military | **N**= 4,563    **Age:** -  **Sex:** -    **Multiple injuries:** -    **Mortality:**  KIA: -  DOW: - | Iraq & Afghanistan    **Time frame:**  January 2005  -  December 2009    **Explosive devices:**  unspecified | **Unknown** | **Nr. of injuries**    **Head/neck**  **Face**  **Thorax**  **Abdomen**  **Extremities**  **Spine**  **Pelvis**  **External**  **Peripheral nerve injuries** | 14,158 (100%)    -  -  -  -  5,798 (41%)  713 (5%)  246 (2%)  6,862 (48%)    539 (4%) | **Nr. of fractures**  5,798(100%)    **Upper extremity injuries:**  2,312 (40%)    **Lower extremity injuries:**  3,486 (60%)    **Total nr. of amputations:**  976 (17%) |
| **Bilukha 2011**    UNICEF & INSEC database    Children & adults    Civilian | **N=** 307    **Age:** 55% < 18  **Sex:** M 213 / F 94    **Nr. of multiple injured:**  79 (25.7%) injured the upper & lower body    **Mortality:**  **KIA:** 35 (11.4%)  **DOW:** 7 (2.3%) | Nepal    **Time frame:**  2006-2010    **Explosive devices:**  Socket bomb, sutali bomb, other IED, landmine, detonator, hand grenade, UXO, unknown | **Unknown**  **severity** | **Nr. of injured persons:**    **Upper body**  **Lower body**  **Upper & lower body**  **unknown** | 307 (100%)    107 (34.9%)  51 (16.6%)  79 (25.7%)  28 (9.1%) | - |
| **Breeze 2011 (a)**    JTTR UK    Adults    Military | **N**= 60  **Age:** -  **Sex:** -    **Multiple injuries:** -    **Mortality:**  **KIA**: -  **DOW**: 38 (63%) | Afghanistan & Iraq    **Time frame:**  January 2004  -  September 2009    **Explosive devices:**  unspecified | **Unknown** | **Nr. of injured persons:**    **Head/neck**  **Face**  **Thorax**  **Abdomen**  **Extremities**  **Spine**  **Pelvis**  **External** | 60 (100%)    -  60 (100%)  -  -  -  -  -  - | - |
| **Breeze 2011 (b)**    JTTR UK    Adults    Military | **N=** 652    **Age:** -  **Sex:** -    **Multiple injuries:** -    **Mortality:**  KIA: -  DOW: - | Afghanistan & Iraq    **Time frame:**  January  2006  -  December 2009    **Explosive devices:**  unspecified | **Unknown** | **Nr. of injured persons:**    **Head/neck**  **Face**  **Thorax**  **Abdomen**  **Extremities**  **Spine**  **Pelvis**  **External** | 652 (100%)    -  76 (12%)  -  -  -  -  -  - | - |
| **Brewster 2021**    Retrospective hospital data    Adults    Military | **N**= 99    **Age:** -  **Sex:** -    **Nr. of multiple injured:**  53 (54%)    **Mortality:**  **KIA:** -  **DOW:** - | Afghanistan    **Time frame:**  2012    **Explosive devices:**  unspecified | **Unknown** | **Nr. of injured persons:**    **Head/neck**  **Face**  **Thorax & Spine**  **Abdomen & Pelvis**  **Extremities**  **External** | 99 (100%)    21 (21%)  -  11 (12%)  16 (18%)  91 (92%)  - | **Nr. of injuries**  91(100%)    **Single limb injuries:**  40 (44%)    **Two limb injuries:**  23 (25%)    **Three limb injuries:**  17 (19%)    **Four limb injuries:**  11 (12%)    **Total nr. of amputations:**  51 (56%) |
| **Cardi 2019**    Retrospective hospital data    Children & adults    Mainly civilians | **N=** 267    **Age:** ⵁ 19.2 (range 10-28)  **Sex:** M 110/ F 9    **Nr. of multiple injured:**  72 (27%)    **Mortality:**  **KIA:** -  **DOW:** 34 (13%) | Afghanistan    **Time frame:**  2006 - 2016  **Explosive devices:**  shell injury (n=246), mine injury (n=21) | **Unknown severity** | **Nr. of injuries:**    **Head**  **Face**  **Thorax**  **Abdomen**  **Extremities**  **Spine**  **Pelvis**  **External**  **Urogenital** | 348 (100%)    -  -  -  343 (99%)  -  -  -  -  5 (1%) | - |
| **Edwards 2012**    JTTR US    Children & adults    Civilians | **N=** 4,913    **Age:** 63% >21 y  **Sex:** M 4,308 / F 601    **Nr. of multiple injured:**  3,439 (70%)    **Mortality**  **KIA:** -  **DOW:** 335 (6.8%) | Afghanistan & Iraq    **Time frame:**  2002 - 2010    **Explosive devices:**  fragments/ mine/ UXO | **AIS ≥ 3** | **Nr. of injured persons all AIS:**    **Head & cervical spine**  **Face**  **Thorax**  **Abdomen**  **Extremities incl. pelvis**  **External (incl. Burns)** | 4913    1,000 (20%)  188 (4%)  972 (20%)  465 (10%)  1,506 (31%)    187 (4%) | - |
|  |  |  | **AIS < 3** | **Nr. of injured persons all AIS:**    **Head & cervical spine**  **Face**  **Thorax**  **Abdomen**  **Extremities incl. pelvis**  **External**  **(incl. Burns)** | 4913    386 (8%)  1,123 (23%)  89 (2%)  657 (13%)    997 (20%)  3,237 (66%) | - |
| **Fleming 2012**    Combat trauma registry (Navy Registry)    Adults    Military | **N**= 109    **Age:** ⵁ 23.2  (range 18 – 39)  **Sex:** M 109 / F 0    **Nr. of multiple injured:**  Min. 63 (57%) more than one major amputation    **Mortality:**  **KIA:** -  **DOW:** 5 (4%) | Afghanistan & Iraq    **Time frame**  2007 - 2010    **Explosives:**  multiple | **Unknown severity** | **Nr. of injuries:**    **Head**  **Face**  **Thorax**  **Abdomen**  **Extremities**  **Spine**  **Pelvis**  **External**  **Urogenital** | 536 (100%)    -  41 (8%)  15 (3%)  17 (3%)  183 (34%)  13 (2%)  23 (4%)  144 (27%)  100 (19%) | **Total nr. of amputations:**  183 (100%)    **Upper extremity amputations:**  13 (7%)    **Lower extremity amputations:**  167 (91%) |
| **Hull 1992**    Case notes & medical record cards    Adults    Military | **N**= 29    **Age:** -  **Sex:** M 29 / F 0    **Multiple injuries:** -    **Mortality:**  **KIA:** -  **DOW:** 2 (7%) | Falkland Islands    **Time frame**  1979 - 1990    **Explosives**  Air-dropped bomb, missile, mortar, grenade,  land mine,  car bomb,  booby trap, IED,  detonation cord, plastic explosive,  aircraft tire explosion | **Unknown severity** | **Nr. of injured persons:**    **Head**  **Face**  **Thorax**  **Abdomen**  **Extremities**  **Spine**  **Pelvis**  **External**  **Urogenital** | 29 (100%)  2 (7%)  7 (24%)  2 (7%)  4 (14%)  29 (100%)  -  -  12 (41%)  2 (7%) | **Nr. of injuries**  41 (100%)    **Upper extremity injuries:**  9 (22%)    **Lower extremity injuries:**  32 (78%)    **Total nr. of amputations:**  41 (100%) |
| **Hull 1994**    Autopsy & police photographs    Adults    Military & civilian | **N=** 34    **Age:** -  **Sex:** -    **Multiple injuries:** -    **Mortality:**  only fatalities investigated | Northern Ireland    **Time frame**  1987 - 1992    **Explosives:**  multiple | **Unknown severity** | **Nr. of injured persons:**    **Head**  **Face**  **Thorax**  **Abdomen**  **Extremities**  **Spine**  **Pelvis**  **External** | 34 (100%)  15 (44%)  -  14 (41%)  22 (65%)  34 (100%)  -  -  - | **Nr. of injuries**  73 (100%)    **Upper extremity injuries:**  17 (23%)    **Lower extremity injuries:**  56 (77%)    **Total nr. of amputations:**  73 (100%) |
| **Hull 1996**    Review of autopsy reports & police photographs    Adults    Military & civilian | **N**= 100    **Age:** -  **Sex:** -    **Multiple injuries:**  100 (100%)    **Mortality:**  only fatalities investigated | Northern Ireland    **Time frame**  1987 - 1992    **Explosives**  Car bombs, IED, booby traps, lorry or van bombs, culvert bombs | **Unknown severity** | **Nr. of injured persons:**    **Head**  **Face**  **Thorax**  **Abdomen**  **Extremities**  **Spine**  **Pelvis**  **External** | 100    -  -  -  -  34 (34%)  -  -  13 (13%) | **Nr. of amputations**  73 (100%)    **Upper extremities:**  17 (23%)    **Lower extremities:**  56 (77%) |
| **Kamauzaman 2007**    Case reports    Adults    Military | **N= 4**    **Age:** ⵁ 32  (range 30-34)  **Sex:** M 4 / F 0    **Multiple injuries:** -    **Mortality**  **KIA:** 2 (50%)  **DOW**: 1 (25%) | Malaysia    **Time frame:**  2000    **Explosive devices:**  hand grenade | **Unknown severity** | **Nr. of injured persons:**    **Head**  **Face**  **Thorax**  **Abdomen**  **Extremities**  **Spine**  **Pelvis**  **External** | 4  1 (25%)  1 (25%)  1 (25%)  1 (25%)  2 (50%)  0 (0%)  0 (0%)  1 (25%) | **Nr. of injured persons**  2 (100%)    **Upper extremity injuries:**  2 (100%)    **Lower extremity injuries:**  -  **Total nr. of amputations:**  1 (25%) |
| **Kauvar 2006**    Retrospective hospital data    Adults    Military | **N**= 142    **Age:** ⵁ 26 (±7)  **Sex:** -    **Multiple injuries:** -    **Mortality:**  **KIA:** -  **DOW:** 5 (4%) | Afghanistan & Iraq    **Time frame**  2003 - 2005    **Explosives:**  IED, vehicle-borne IED, rocket-propelled grenade, mortar, landmines,  explosive devices & vehicle-borne improvised devices (car  bombs | **Unknown severity** | **Nr. of injured persons:**    **Head**  **Face**  **Thorax**  **Abdomen**  **Extremities**  **Spine**  **Pelvis**  **External incl.**  **Burns**  **Urogenital** | 142 (100%)      -  -  -  -  -  -  -    142 (100%)  - | - |
| **Khan 2015**    Retrospective hospital data    Children & adults    Civilian | **N=** 262    **Age:** ⵁ 31 (±14)  **Sex:** M 220 / F 42    **Multiple injured:** -    **Mortality:**  **KIA:** -  **DOW:** - | Pakistan    **Time frame:**  January 2008  -  December 2012    **Explosive devices:** - | **Unknown** | **Nr. of injured persons:**  **Head**  **Face**  **Thorax**  **Abdomen**  **Extremities**  **Spine**  **Pelvis**  **External** | 262 (100%)  10 (4%)  14 (5%)  25 (10%)  -  65 (25%)  -  -  75 (29%) | **Nr. of injuries**  65 (100%)    **Upper extremity injuries** (amputations):  5 (8%)    **Lower extremity injuries** (amputations): 5 (8%)    **Total nr. of amputations:**  10 (15%) |
| **Khan 2017**    Retrospective hospital data    Children & adults    Civilian | **N**= 56    **Age:** ⵁ 28 (±8.3)  **Sex:** M 51 / F 5    **Multiple injuries:** -    **Mortality**  **KIA:** -  **DOW:** - | Pakistan    **Time frame:**  April 2012  -  June 2015    **Explosive devices:**  unspecified | **Unknown** | **Nr. of injured persons:**    **Head**  **Face**  **Thorax**  **Abdomen**  **Extremities**  **Spine**  **Pelvis**  **External**  **Urogenital** | 56 (100%)  -  -  -  47 (84%)  -  -  -  12 (21%)  5 (9%) | - |
| **Korkmaz 2022**    Retrospective hospital data    Children    Civilian | **N=** 74    **Age:** ⵁ 9.19 (±4.5)  **Sex:** M 51 / F 23    **Nr. of multiple injured:**  33 (45%)    **Mortality:**  **KIA:** -  **DOW:** - | Syria    **Time frame:**  2015-2020    **Explosive devices:**  bomb explosion | **Unknown** | **Nr. of injuries:**    **Head**  **Face**  **Thorax**  **Abdomen**  **Extremities**  **Spine**  **Pelvis**  **External**  **Others** | 152 (100%)    24 (16%)  -  59 (39%)  15 (10%)  45 (29%)  9 (6%)  -  -  - | **Nr. of injuries**  45 (100%)    **Upper extremity:**  14 (31%)    **Lower extremity:** 31 (69%)    **Total nr. of amputations:**  11 (24%) |
| **Korkmaz 2023a**    Retrospective hospital data    Children    Civilian | **N=** 36    **Age: M** ⵁ 8 (±3.95)  **F** ⵁ7.44 ±4.31  **Sex:** M 25 / F 11    **Multiple injured:** -    **Mortality:**  **KIA:** -  **DOW:** 13 (36%) | Syria    **Time frame:**  2011-2020    **Explosive devices:**  bomb explosion | **Unknown** | **Nr. of injured persons:**  **Head**  **Face**  **Thorax**  **Abdomen**  **Extremities**  **Spine**  **Pelvis**  **External**  **Others** | 36 (100%)  14 (9%)  -  36 (100%)  21 (13%)  -  -  -  -  - | - |
| **Korkmaz 2023b**    Retrospective hospital data    Children    Civilian | **N=** 65    **Age:** ⵁ 9.35 (±4.26)  **Sex:** M 46 / F 19    **Multiple injured:** -    **Mortality:**  **KIA:** -  **DOW:** 14 (22%) | Syria    **Time frame:**  2011-2022    **Explosive devices:**  bomb explosion | **Unknown** | **Nr. of injuries:**    **Head**  **Face**  **Thorax**  **Abdomen**  **Extremities**  **Spine**  **Pelvis**  **External**  **Others** | 205 (100%)    24 (12%)  -  22 (10%)  109 (53%)  20 (10%)  6 (3%)  -  20 (10%)  4 (2%) bladder injuries | **Nr. of injuries**  20 (100%)  **Upper extremity:**  5 (25%)    **Lower extremity:**  15 (75%)    **Total nr. of amputations:**  **-** |
| **Luria 2013**    retrospective & prospective hospital Data    Adults    Civilian | **N**= 72    **Age:** ⵁ 27.3 (±12.3)  **Sex:** M 31 / F 41    **Multiple injuries:** -    **Mortality**  **KIA:** -  **DOW:**  4 (6%) | Israel    **Time frame:**  October 2000  -  February  2004    **Explosive devices:**  bomb explosion | **Unknown** | **Nr. of injured persons:**  **Head & Neck**  **Face**  **Thorax**  **Abdomen**  **Extremities**  **Spine**  **Pelvis**  **External** **incl. burns**  **Vascular injuries**  **Nerve injuries** | 72 (100%)  23 (33%)  -  18 (25%)  9 (13%)  72 (100%)  -  -  30 (43%)  7 (10%)  16 (22%) | **Nr. of injuries**  98 (100%)    **Upper extremity:**  98 (100%)    **Lower extremity:**  28 (29%)    **Total nr. of amputations:**  5 (5%) |
| **Mellor 1992**  Retrospective analysis of hostile action casualty system    Military    Adults | **N=** 828  **Age:** -  **Sex:** -  **Nr. of multiple injured:**  103 (12%)    **Mortality**  **KIA:** 177 (21%)  **DOW:** 39 (5%) | North Ireland    **Time frame:**  1970-1984    **Explosive device:**  multiple | **Unknown severity** | **Nr. of injured persons:**  **Head**  **Face**  **Thorax**  **Abdomen**  **Extremities**  **Spine**  **Pelvis**  **External incl. burns** | 828 (100%)  265 (32%)  288 (35%)  4 (0.4%)  1 (0.1%)  298 (36%)  -  -  31 (4%) | **Nr. of injured persons:**  298 (100%)    **Upper extremity injuries:**  89 (30%)    **Lower extremity injuries:**  106 (36%)    **Total nr. of amputations:**  52 (18%) |
| **Owens 2008**    US JTTR    Adults    Military | **N=** 1,146    **Age:** -  **Sex:** -    **Nr. of multiple injured:**  ⵁ 4.2 injuries per person    **Mortality**  **KIA:** -  **DOW:** - | Afghanistan & Iraq    **Time frame:**  2001 - 2005    **Explosive devices:**  Improvised explosive device (IED), landmine, mortar or shrapnel, bomb, grenade | **Unknown severity** | **Nr. of injured persons:**  **Head/neck/**  **face/**  **cervical spine**  **Thorax/**  **thoracic spine**  **Abdomen/**  **Pelvis/lumbar spine**  **Extremities**  **External** | 1,146 (100%)  1,008 (88%)  893 (78%)  928 (81%)  928 (81%)  - | - |
| **Peleg 2004**    ITR    Adults    Civilians & military | **N**= 623    **Age:** 15-29: 297 (49%)  **Sex:** M 392 / F 231    **Nr. of multiple injured:**  597 (96%)    **Mortality:**  **KIA:** -  **DOW:** 33 (5%) | Israel    **Time frame:**  October 2000 to  June 2002    **Explosive devices:**  unknown | **Unknown** | **Nr. of injuries:**  **Head**  **Face**  **Thorax**  **Abdomen**  **Extremities**  **Spine**  **Pelvis**  **External incl. burns** | 1,691 (100%)  450 (27%)  -  135 (8%)  75 (4%)  476 (28%)  30 (2%)  104 (6%)  421 (25%) | **Nr. of injuries**  476 (100%)    **Nr. of fractures**  193 (41%)    **Upper extremity injuries:**  242 (51%)    **Lower extremity injuries:**  234 (49%) |
| **Popivanov 2014**    Retrospective hospital data    Children & adults    Mainly civilians | **N=** 55    **Age:** ⵁ 23.6  (range 5-70)  **Sex:** M 51 / F 4    **Nr. of multiple injured:**  40 (73%)    **Mortality:**  **KIA:** -  **DOW:** - | Afghanistan    **Time frame:**  September 2010 to December 2010 & January 2010 to July 2010    **Explosive devices:**  unspecified | **Unknown** | **Nr. of injured persons:**    **Head**  **Neck**  **Face**  **Thorax**  **Abdomen**  **Extremities**  **Spine**  **Pelvis**  **External incl burns** | 55 (100%)  15 (27%)  6 (11%)  11 (20%)  21 (38%)  14 (25%)  47 (85%)  -  -  7 (13%) | **Nr. of injuries:**  47 (100%)    **Upper extremity injuries:**  18 (38%)    **Lower extremity injuries:**  29 (53%)    **Fractures:**  7 (15%) |
| **Ramasamy**  **2009**    Retrospective hospital data    Adults    Civilian & Military | **N**= 62    **Age:** -  **Sex:** -    **Nr. of multiple injured:**  52 (84%)    **Mortality:**  **KIA:** -  **DOW: 2** (3%) | Iraq    **Time frame**  January 2006- October 2006    **Explosives**  unspecific | **Unknown severity** | **Nr. of injuries:**  **Head/neck**  **Face**  **Thorax**  **Abdomen**  **Extremities**  **Spine**  **Pelvis**  **External** | 192 (100%)  18 (10%)  25 (11%)  6 (4%)  1 (1%)  142 (70%)  -  6 (4%)  - | **Nr. of injuries**  142 (100%)    **Upper extremity injuries:**  54 (38%)    **Lower extremity injuries:**  88 (62%)    **Total nr. of amputations:**  4 (3%) |
| **Ramasamy 2011**    UK JTTR    Adults    Military | **N**= 30    **Age:** ⵁ 26.2 (±5.7)  **Sex:** M 29 / F 1    **Nr. of multiple injured:**  27 (90%)    **Mortality:**  **KIA:** -  **DOW:** - | Afghanistan & Iraq    **Time frame**  2006 - 2008    **Explosives**  Vehicle explosion | **Unknown severity** | **Nr. of injured persons:**  **Head/Face**  **Thorax**  **Abdomen**  **Extremities**  **Spine**  **Pelvis**  **External incl. burns** | 30 (100%)  7 (23%)  1 (3%)  -  30 (100%)  9 (30%)  1 (3%)  3 (10%) | **Nr. of injuries**  87 (100%)    **Upper extremity injuries:**  3 (3%)    **Lower extremity injuries:**  66 (76%)    **Total nr. of amputations:**  18 (21%) |
| **Ritenour 2010**    JTTR US    Adults    Military | **N**= 4,765    **Age:** ⵁ 26 (±7)  **Sex:** M 4,622 / F 143    **Multiple injuries:** -    **Mortality**  **KIA**: -  **DOW:** 67 (1%) | Worldwide    **Time frame:**  2003-2006    **Explosive device:**  IED; rocket-propelled grenades & grenades; mortars; landmines; other | **Unknown severity** | **Nr. of injured persons (only primary blast injured):**  **Head**  **Face**  **Thorax**  **Abdomen**  **Extremities**  **Spine**  **Pelvis**  **External** | 582 (100%)  -  425 (77%)  172 (30%)  5 (1%)  -  -  -  - | - |
| **Rozenfeld 2023**    ITR    Children & adults    Civilians & military | **N**= 2,533    **Age:** 60% 15-29y  **Sex:** M 1,997 / F 536    **Multiple injuries:** -    **Mortality**  **KIA**: -  **DOW:** 123 (5%) | Israel    **Time frame:**  January 1997 - December 2018    **Explosive device:**  terror-related events (all terror-related explosions -mainly suicide bombers and car bombs)  war-related events (all non-civilian explosive events defined as war) | **AIS>3** | **Nr. of injuries:**  **Head**  **Face**  **Thorax**  **Abdomen**  **Extremities**  **Spine**  **Pelvis**  **External** | 438(100%)  205 (47%)  -  139 (32%)  94 (21%)  -  -  -  - | **Total nr. of extremity injuries:**  1956 (100%)  **Upper extremities:**  888 (45%)    **Lower extremity:**  1068 (55%)    **Total amputations:** 89 (5%)   - Lower extremity: 40 - upper extremity: 49 |
|  |  |  | **Unknown severity** | **Nr. of injuries:**  **Head**  **Face**  **Thorax**  **Abdomen**  **Extremities**  **Spine**  **Pelvis**  **External**  **Burns** | 6,005 (100%)  776 (13%)  1,102 (18%)  725 (12%)  606 (10%)  1,956 (33%)  83 (1%)  -  469 (8%)  288 (5%) |  |
| **Sargent 2024**    UK military radiological database & JTTR UK    Children    Civilian | **N**= 100    **Age:** ⵁ 10 (range 6-12)  **Sex:** -    **Nr. of multiple injured:**  24 (24%)    **Mortality**  **KIA**: -  **DOW:** - | Afghanistan    **Time frame:**  2011    **Explosive device:** - | **AIS ≥ 3** | **Nr. of injured persons:**  **Head**  **Face**  **Thorax**  **Abdomen**  **Extremities**  **Spine**  **Pelvis**  **External** | 100 (100%)  14 (14%)  -  15 (15%)  20 (20%)  33 (33%)  -  -  - | - |
| **Sayer 2008**    Retrospective hospital data    Adults    Military | **N**= 106    **Age:** -  **Sex:** -    **Multiple injuries:**  Mdn 5 injuries per person (Range 1-9)    **Mortality**  **KIA:** -  **DOW:** 3 (3%) | Afghanistan & Iraq    **Time frame:**  2001-2006    **Explosive devices:**  IEDs, RPGs, mortar, hand grenades, bomb, landmines, & other explosions | **Unknown severity** | **Nr. of injuries**    **Head**  **Face**  **Thorax**  **Abdomen**  **Extremities**  **Spine**  **Pelvis**  **External incl.**  **Burns**  **Urogenital** | 486 (100%)    127 (26%)  153 (31%)  41 (8%)  13 (3%)  59 (12%)  3 (1%)  -    90 (19%)  0 (0%) | **Nr. of injuries**  59 (100%)    **Fractures:**  48 (81%)    **Total nr. of amputations:**  10 (17%) |
| **Shakargy 2022**    Israel defense forces postmortem (IDF-PM) registry    Adults    Military | **N=** 222    **Age:** Mdn 22  (IQR 20.26)  **Sex:** M 217 / F 5    **Multiple injuries:**  Mdn nr of injured body region per casualty: 4 (IQR 2.6)    **Mortality**  222 (100%)  **KIA:** -  **DOW:** - | Israel    **Time frame:**  1982 - 2021    **Explosive devices:**  unspecified | **AIS ≥ 1 incl. AIS ≥ 3** | **Nr. of injured body region**  **Head**  **Face**  **Thorax**  **Abdomen**  **Upper Extremities**  **Lower Extremities**  **Spine**  **Pelvis**  **External** | 596 (100%)  91 (15%)  75 (13%)  93 (16%)  61 (10%)  135 (23%)  141 (24%)  12 (2%)  -  63 (11%) | **Nr. of injuries**  275 (100%)    **Upper extremity injuries:**  134 (49%)    **Lower extremity injuries:**  141 (51%)    **Total nr. of amputations:**  - |
|  |  |  | **Only**  **AIS ≥ 3** | **Nr. of injured body regions:**  **Head**  **Face**  **Thorax**  **Abdomen**  **Upper extremities**  **Lower extremities**  **Spine**  **Pelvis**  **External** | 107 (100%)  30 (11%)  1 (1%)  27 (10%)  11 (4%)  11 (12%)  21 (20%)  3 (3%)  -  3 (3%) |  |
| **Surani 2015**    Retrospective hospital data    Children & adults    Civilians | **N**= 481    **Age:** ⵁ 31.4 (±12.3)  **Sex:** M 463 / F 18    **Nr. of multiple injured:**  275 (57%)    **Mortality:**  **KIA:** -  **DOW:** - | Pakistan    **Time frame:**  2000-2007    **Explosive devices:**  Suicide bomber, remote explosive devices, combination remote & suicide bomber | **Unknown severity** | **Nr. of injuries:**    **Head/neck**  **Face**  **Thorax**  **Abdomen**  **Extremities**  **Spine**  **Pelvis**  **External incl.**  **burns** | 896 (100%)    147 (16%)  -  -  -  518 (58%)  -  -  809 (90%) | **Nr. of injuries**  518 (100%)    **Upper extremity injuries:**  170 (33%)    **Lower extremity injuries:**  348 (67%)    **Total nr. of amputations:**  4 (0.7%) |
| **Thompson 2017**    UK JTTR    Children    Civilians | **N=** 295    **Age:** Mdn M 12 / F 8  **Sex:** M 225 / F 70    **Multiple injuries:**  Mdn 2 injuries (Range 1-8) per person    **Mortality:**  **KIA:** -  **DOW:** 54 (19%) | Afghanistan    **Time frame:**  2006-2013      **Explosive devices:**  IED, mortar, rocket, UXO, bombs, mine, rocket-propelled grenades, grenades,  other,  unspecified | **AIS ≥ 1 (incl. AIS ≥3)** | **Nr of injuries:**    **Head/Neck**  **Face:**  **Thorax:**  **Abdomen:**  **Extremities**  **Spine**  **Pelvis:**  **External:** | 1572 (100%)    172 (11%)  186 (12%)  165 (10%)  227 (14%)  723 (46%)  22 (1%)  -  36 (2%) | **Nr. of injuries:**  **723 (100%)**  **Upper extremity injuries:**  237 (33%)  **Lower extremity injuries:**  486 (67%) |
|  |  |  | **AIS ≥3** | **Nr. of injured persons:**    **Head**  **Neck**  **Face**  **Thorax**  **Abdomen**  **Extremities**  **Spine**  **Pelvis**  **External** | 281 (100%)    63 (22%)  3 (1%)  0 (0%)  52 (19%)  44 (16%)  103 (45%)  4 (1%)  -  9 (3%) | **Upper extremity injuries:**  15 (5%)    **Lower extremity injuries:**  88 (31%)    **Total nr. of amputations:**  25 (12%) |
| **Wani 2009**  Retrospective hospital data    Civilians    - | **N=** 154    **Age:** -  **Sex:** M 124 / F 30    **Multiple injuries:** -    **Mortality**  KIA: -  DOW: 6 (4%) | Kashmir, Pakistan    **Time frame:**  1998-2008    **Explosive device:**  multiple | **Unknown severity** | **Nr. of injured persons:**    **Head**  **Face**  **Thorax**  **Abdomen**  **Extremities**  **Spine**  **Pelvis**  **External**  **Urogenital** | 154 (100%)  -  -  -  154 (100%)  -  -  -  -  1 (0.6%) | - |
| **Weil 2011**    ITR    Children & adults    Civilians | **N**= 694    **Age:** 53% 15-29  **Sex:** M 457 / F 237    **Nr. of multiple injured:**  375 (54%)    **Mortality:**  **KIA:** -  **DOW:** 32 (5%) | Israel    **Time frame:**  October 2000  -  December 2005    **Explosive devices:** - | **Unknown** | **Nr. of injuries:**    **Head/Neck**  **Face**  **Thorax**  **Abdomen**  **Extremities**  **Spine**  **Pelvis**  **External** | 875 (100%)    351 (40%)  -  188 (21%)  163 (19%)  -  -  -  173 (20%) | - |
| **Wordsworth 2017**    JTTR UK    Adults    Civilians & military | **N**= 504    **Age:** ⵁ 26  **Sex:** -    **Multiple injuries:**  Mdn nr. of injuries 8 (range 1-57)    **Mortality:**  **KIA:** 112 (22%)  **DOW:** 16 (3%) | Afghanistan    **Time frame:**  April 1, 2006  -  March 01, 2013    **Explosive devices:**  multiple | **Unknown** | **Nr. of injured persons:**    **Head/neck**  **Face**  **Thorax**  **Abdomen**  **Extremities**  **Spine**  **Pelvis**  **External incl.**  **burns** | 504 (100%)  215 (43%)  504 (100%)  -  -  336 (67%)  -  -  14 (4%) | **Lower extremity injuries:**  336 |

Legend: DOW = died of wounds; F = Female; IED = improvised explosive device; INSEC = Informal Sector Service Center; IRT = Israel National Trauma Registry; JTTR = Joint Theatre Trauma Registry; KIA = killed in action; M = male; Mdn = median; N = sample size; nr. = number; RPG = rocket-propelled grenade; UK = United Kingdom; UNICEF =; US = United States of America; UXO = Unexploded Ordnance

- = not reported; ⵁ = mean; ± = standard deviation;

Color indicates the same data source: JTTR UK/USA; ITR; Northern Ireland conflict

## B) Overview specific body parts

| **Body part** | **Study ID** | **Severity of injuries** | **Specifics of the Injuries**  **– nr. of injuries (%)** | |
| --- | --- | --- | --- | --- |
| **Head injuries** | **Bashir 2013** | **Unknown** | **Nr. of persons with head injuries:**   - skull fractures - basilar fractures - penetrating injuries to the head (shrapnel or bone fragments) - contusion - oedema - subdural hematoma - intraventricular hemorrhage   subarachnoid hemorrhage | **16**  13 (81%)  3 (19%)    13 (81%)  14 (88%)  7 (44%)  2 (13%)  1 (6%)  1 (6%) |
| **facial trauma** | **Wordsworth 2017** | **Unknown** | **Total nr. facial injuries**   - isolated face injuries - orbit fractures      - Soft tissue injuries   - mid-facial   - upper face   - lower third of the face   - burns   - facial nerve injuries | **504**  36 (7%)  34 (7%)    193 (38%)  39 (8%)  142 (28%)  14 (3%)  4 (0.7%) |
| **mandibular fractures** | **Breeze 2011 (a)** | **Unknown** | **Total nr. of mandibular fractures**   - simple fractures   - blast wave   - fragmentation      - comminuted fractures   - blast wave   - fragmentation | **74**  **43 (58%)**  37 (50%)  6 (8%)    **31 (42%)**  10 (14%)  21 (28%) |
| **ear injuries** | **Breeze 2011 (b)** | **Unknown** | **Total nr. of ear injuries**   - External ear injuries - tympanic membrane injuries - ossicular injuries | **76**  18 (24%)  52 (68%)  6 (8%) |
| **Extremity injuries/ musculoskeletal injuries** | **Belmont 2011** | **Unknown** | **Total nr. of upper extremity injuries**   - **Nr. of humerus fractures**   - open   - closed      - **nr. of radius/ulna fractures**   - open   - closed      - **nr. of carpal fractures**   - open   - closed      - **nr. of phalange fractures**   - open   - closed   **Total nr. of lower extremity injuries:**     - **Nr. of femur fractures**   - open   - closed      - **nr. of tibia fractures**   - open   - closed      - **nr. of fibular fractures**   - open   - closed      - **nr. of tarsal/metatarsal fractures**   - open   - closed | **17**  **2 (12%)**  2 (12%)  -  **3 (18%)**  2 (12%)  1(6%)    **7 (41%)**  2 (12%)  5 (28%)    **6 (35%)**  3 (18%)  3 (18%)    **13 (%)**    **1 (8%)**  1 (8%)  0 (0%)    **6 (46%)**  2 (15%)  4 (31%)    **3 (23%)**  1 (8%)  2 (15%)  **3 (23%)**  2 (15%)  1 (8%) |
|  | **Belmont 2013** | **Unknown** | **Total nr. of injuries**    **Total nr. of fractures**  **Total Nr. of axial skeleton fractures**   - Spine fracture - Pelvis/acetabulum fracture     **Total Nr. of upper extremity fracture**   - Shoulder girdle - Humerus fracture - Radius/ulna fracture (forearm) - Hand     **Total Nr. of lower extremity fracture**   - Femur fracture - Patella fracture - Tibia/fibula fracture - Ankle fracture - Foot     **Total Nr. of Amputations**   - Arm amputations - Leg amputations - Foot amputations - Finger amputations - Toe amputations     **Total Nr. of neurologic injuries**   - Spinal cord injuries - Nerve root injuries - Peripheral nerve injuries     **Total Nr. of joint dislocations**   - Shoulder - Elbow - Wrist - Finger - Hip - Knee/patella - Ankle - Foot - Spine     **Total Nr. of soft tissue injuries**   - Disc displacement - Neck/spine soft tissue injuries - Buttock soft tissue injuries - Arm - Forearm/hand - Thigh hip soft tissue injuries - Leg | **14,158**    **5,420 (38%)**  **841 (6%)**  595 (4%)  246 (2%)    **1,917 (14%)**  166 (1%)  316 (2%)  601 (4%)  834 (6%)    **2,662 (19%)**  455 (3%)  114 (1%)  1,003 (7%)  356 (2%)  712 (5%)    **976 (7%)**  125 (1%)  532 (4%)  95 (1%)  180 (1%)  44 (0.3%)    **596 (4%)**  45 (0.3%)  12 (0.1%)  539 (4%)    **304 (2%)**  23 (0.2%)  26 (0.2%)  17 (0.1%)  24 (0.2 %)  17 (0.1%)  29 (0.2%)  69 (0.5%)  38 (0.3%)  61 (0.4%)    **6,862 (48%)**  21 (0.2%)  86 (0.6%)  169 (1%)  1,350 (10%)  1,760 (12%)  1,183 (8%)  2,293 (16%) |
|  | **Luria 2013** | **Unknown** | **Total nr. of injuries**  **Shoulder**   - Amputation - Open fracture - Fracture - Soft tissue injury alone - Burn - Foreign body - Tendon injury     **Arm**   - amputation - open fracture - fracture - soft tissue injury alone - burn - Foreign body - tendon injury     **Elbow**   - amputation - open fracture - fracture - soft tissue injury alone - burn - Foreign body - tendon injury     **Forearm**   - amputation - open fracture - fracture - soft tissue injury alone - burn - Foreign body - tendon injury     **Wrist**   - amputation - open fracture - fracture - soft tissue injury alone - burn - Foreign body - tendon injury     **Hand**   - amputation - open fracture - fracture - soft tissue injury alone - burn - Foreign body - tendon injury   **not specified**   - burn - foreign body | **126**  **16 (13%)**  0  4  1  5  0  6  0    **18 (14%)**  1  4  0  4  3  6  0    **12 (10%)**  0  2  1  7  1  1  0    **25 (20%)**  1  10  1  3  2  8  0    **8 (6%)**  0  0  1  0  1  4  2    **39 (31%)**  3 (fingers)  5  1  8  17  2  3    **8 (6%)**  7  1 |
|  | **Ramasamy 2011** | **Unknown** | **Total nr. of injuries**    **Nr. of upper extremity injuries**:    **Nr. of lower extremity injuries**   - Femur - Tibia/ Fibula - Calcaneus   **Total nr. of Extremity amputations**   - Field amputation - Early amputation - Delayed amputation | -    **3**    **63**  3 (5%)  20 (32%)  40 (63%)    **18**  11 (61%)  3 (17%)  4 (22%) |
| **amputations** | **Fleming 2012** | **Unknown** | **Total nr. of amputations**   - number of patients with at least 2 amputations - upper extremity amputations - lower extremity amputations | **183**  63 (34%)  16 (9%)  167 (91%) |
|  | **Hull 1992** | **Unknown** | **Total nr. of injuries**    **Nr. of upper extremities injuries**  - Amputations  - Hand fractures  - Fragment wounds to the arms or hands- partial amputation    **Nr. of lower extremity injuries**  - Above knee amputations  - Upper third of the tibia  - Fragment wounds to the legs    **Nr. of extremity amputations**  Number of patients with at least 2 limb amputations | -    **18**  9 (50%)  4 (22%)  4 (22%)  1 (6%)    **41**  14 (34%)  16 (39%)  9 (22%)    **41**  17 (41%) |
|  | **Hull 1994** | **Unknown** | **Nr. of traumatic extremity amputation**    **Upper extremity injuries/ amputations**   - clavicle - shoulder - upper arm   - proximal third   - middle third   - distal third - Elbow - forearm   - proximal third   - middle third   - distal third - wrist     **Lower extremity injuries/ amputations**   - pelvis - hip - thigh   - proximal third   - middle third   - distal third - knee - lower leg   - proximal third   - middle third   - distal third - ankle | **73**    **17 (23%)**  3  0  4  2  0  0    4  2  2  0  **56 (77%)**  6  0  6  8  6  1  19  6  4  0 |
|  | **Hull 1996** | **Unknown** | **Nr. of persons with severe traumatic extremity injuries**    **Total nr of Traumatic extremity amputations**   - Upper extremity injuries/ Amputations - Lower extremity injuries/ Amputations | **34**    **73**  17 (23%)  56 (77%) |
| **abdominal injuries** | **Khan 2017** | **Unknown** | **Total nr. of abdominal injuries**    **hollow viscus injuries**   - Stomach - Duodenum - Jejunum - Ileum - Colon - Rectum - Bladder /Ureter     **Solid organ injuries**   - Liver - Spleen - Pancreas - Mesentery - Kidney - Testis | **130**    **65 (50%)**  11 (8%)  3 (2%)  8 (6%)  23 (18%)  14 (11%)  2 (2%)  4 (3%)    **65 (50%)**  21 (16%)  14 (11%)  3 (2%)  19 (15%)  6 (5%)  2 (2%) |
|  | **Cardi 2019** | **Unknown** | **Nr. of shell & mine injuries total**   - Bowel & Colon - Liver - Stomach - Kidney - Spleen - Diaphragm - Rectum - Duodenum - Bladder | **387**  238 (62%)  49 (13%)  35 (9%)  10 (3%)  13 (3%)  19 (5%)  9 (2%)  9 (2%)  5 (1%) |
|  |  |  | **Nr. of shell injuries**   - Bowel & Colon - Liver - Stomach - Kidney - Spleen - Diaphragm - Rectum - Duodenum - Bladder   **Nr. of mine injuries**   - Bowel & colon - Liver - Stomach - Kidney - Spleen - Diaphragm - Rectum - Duodenum - Bladder | **348**  214 (61%)  40 (11%)  32 (9%)  9 (3%)  12 (3%)  19 (5%)  8 (2%)  9 (3%)  5 (1%)  **39**  24 (62%)  9 (23%)  3 (8%)  1 (3%)  1 (3%)  0 (0%)  1 (3%)  0 (0%)  0 (0%) |
|  | **Wani 2009** | **Unknown** | **Nr. of persons with abdominal injuries**   - **Intestinal perforation & small gut injuries**   - Small intestinal injury   - Large gut injuries   - Ileum injuries   - Jejunal perforations   - Multiple small gut perforations   - Ileal perforations   - Duodenal injury      - **Large gut injuries**   - Transverse colon perforations   - Caecal perforation   - Transaction of an appendix      - **Other abdominal injuries**   - Liver perforation   - Splenic laceration   - Mesenteric tear   - Gastric, duodenal and pancreatic injury   - Individual liver laceration   - Isolated splenic trauma   - Retroperitoneal hematoma | **154**  **58 (38%)**  48 (31%)  10 (7%)  40 (26%)  8 (5%)  37 (24%)  27 (19%)  6 (4%)  3 (%)  **12 (8%)**  6 (4%)  4 (3%)  2 (1%)  **61 (40%)**  4 (3%)  2 (1%)  2 (1%)  1 (1%)  17 (11%)  25 (16%)  10 (7%) |
| **Burn injuries** | **Kauvar 2006** | **Unknown** | **Nr. of burn injuries:**   - Hand - Head - Forearm - Upper arm - Thigh - Leg - Torso - Neck - Foot - Genitals | **556**  114 (21%)  109 (20%)  71 (13%)  54 (10%)  51 (9%)  51 (9%)  51 (9%)  40 (7%)  9 (2%)  6 (1%) |
